# Phylogenetic Analysis of South African Bovine Leukaemia Virus (BLV) Isolates

**DOI:** 10.3390/v12080898

**Published:** 2020-08-17

**Authors:** Akiko Suzuki, Rosamund Chapman, Nicola Douglass, Olivia Carulei, Johan van Rensburg, Anna-Lise Williamson

**Affiliations:** 1 Division of Medical Virology, Department of Pathology, Faculty of Health Sciences, University of Cape Town, Cape Town 7925, South Africa; szkaki001@myuct.ac.za (A.S.); niki.douglass@uct.ac.za (N.D.); olivia.carulei@uct.ac.za (O.C.); anna-lise.williamson@uct.ac.za (A.-L.W.); 2 Institute of Infectious Disease and Molecular Medicine, University of Cape Town, Cape Town 7925, South Africa; 3 Afrivet, Johannesburg 0081, South Africa; johan.vanrensburg@afrivet.co.za

**Keywords:** bovine leukaemia virus, phylogenetic analysis, BLV envelope, BLV *gag*, South African BLV genotypes

## Abstract

Bovine leukaemia virus (BLV) causes chronic lymphoproliferative disorder and fatal lymphosarcoma in cattle, leading to significant economic losses in the beef and dairy industries. BLV is endemic globally and eleven genotypes have been identified. To date, only Zambian isolates have been genotyped from Africa. Although high BLV prevalence has been reported in South Africa, there has been no molecular characterisation of South African BLV isolates. To characterise BLV isolates in South Africa for the first time, we investigated the phylogenetic relationships and compared the genetic variability of eight South African BLV isolates with BLV isolates representing the eleven known genotypes from different geographical regions worldwide. Phylogenetic analyses based on full-length and partial *env* sequences as well as full-length *gag* sequences revealed that at least two genotypes, genotypes 1 (G1) and 4 (G4), are present in cattle in South Africa, which is consistent with studies from Zambia. However, our analysis revealed that the G1 South African isolate is more similar to other G1 isolates than the G1 Zambian isolates whereas, the G4 South African isolates are more divergent from other G4 isolates but closely related to the G4 Zambian isolate. Lastly, amino acid sequence alignment identified genotype-specific as well as novel amino acid substitutions in the South African isolates. The detection of two genotypes (G1 and G4) in southern Africa highlights the urgent need for disease management and the development of an efficacious vaccine against local strains.

## 1. Introduction

Bovine leukosis is the most common neoplastic disease in cattle and consists of two types of leukosis: enzootic bovine leukosis (EBL) and sporadic bovine leukosis (SBL). The occurrence of EBL is more frequent than that of SBL [1,2]. SBL is not transmissible and the causative agent of SBL remains unknown. The aetiological agent of EBL is bovine leukaemia virus (BLV), which is a B-lymphotropic oncovirus, belonging to the *Deltaretrovirus* genus of the *Retroviridae* family [3]. BLV naturally infects cattle, water buffalo and zebu [4]. The majority of transmission is mediated by direct transfer of infected body fluids during iatrogenic procedures such as ear tattooing, dehorning, re-use of needles gloves and plastic sleeves for veterinary procedures, including rectal palpation for the detection of pregnancy and artificial insemination for embryo transfer [5,6,7,8,9], which are common practices in the management of dairy and beef cattle.

EBL is a chronic infection characterised by abnormal proliferation of non-neoplastic B cells during the persistent lymphocytosis (PL) stage culminating in the development of malignant neoplastic lymphosarcoma in various organs [10,11]. The lymphosarcoma stage is accompanied by increased monoclonal populations of neoplastic B lymphocytes and eventually leads to death [12].

The complete BLV RNA genome consists of 8714 bp. As a complex retrovirus, the BLV genome has a conserved modular genomic organisation with a set of structural genes (*gag,* protease (*pro*), polymerase (*pol*) and *env*) at the 5′ end of the genome, followed by accessory genes (*G4*, *R3* and microRNAs) and regulatory genes (*tax*, *rex*) at the 3′ end. Two identical long terminal repeats (LTRs) are present at the 5′ and 3′ ends of the genome. The BLV envelope (Env) proteins are glycoproteins presented on the surface of the virion as a trimer of surface (gp51) and transmembrane protein (gp30) heterodimers. The *env* gene is 1548bp in size and is translated first into the Env precursor (pr72), which is subsequently cleaved by subtilisin/kexin-like convertases (e.g., furin) into the gp51 and gp30 glycoproteins [13]. The gp51 surface protein is rich in binding domains and epitopes such as putative receptor binding domain (RBD) [14,15], antibody epitopes (conformational and linear epitopes) [16,17,18,19,20,21], T-cell epitopes [22,23], neutralising domains [19,24,25,26] and Zn^2+^-binding site [15]. These domains and epitopes are important in cell fusion and entry during natural infection as well as immunogenicity. The gp30 transmembrane protein contains a fusion peptide at the N-terminus, which is important for cell fusion and entry [27,28], and a cytoplasmic tail at the C-terminus, which is essential for viral entry and immune evasion [29,30,31]. Unlike gp51 [32,33], gp30 is not rich in domains and epitopes and is poorly immunogenic [34].

Gag is another structural protein essential for BLV virion formation. The *gag* gene is 1182 bp in size and encodes the Gag-Pro precursor (pr66) from the Gag-Pro-Pol mRNA [35,36]. The Gag-Pro precursor is then, proteolytically cleaved by the viral protease into the Gag precursor (pr45) [37]. The Gag pr45 is further processed by the viral protease into the matrix (p15), capsid (p24) and nucleocapsid (p12) proteins [37]. The capsid is another major target of the host immune response but unlike anti-gp51 antibodies, which are persistently detectable in leukemic and aleukemic animals with low proviral loads, the anti-p24 antibodies are undetectable in aleukemic animals with low proviral loads [38].

Currently, BLV is endemic globally and is detected on all continents, except Antarctica [39]. However, twenty-two European countries had achieved EBL-free status by 2017 and EBL remains present in only seven countries in Europe (Bulgaria, Croatia, Greece, Hungary, Malta, Portugal and Romania) [40]. As of 2019, BLV isolates have been classified into eleven genotypes (G1 to G11). In 2009, Rodríguez et al. [41] conducted a seminal study by comparing the BLV full-length *env* sequences of 28 Argentine isolates and 46 sequences that had been previously described in various studies but had no standardised classification amongst different studies. Using neighbour-joining, Bayesian, maximum-likelihood and parsimony methods, they re-classified these isolates into seven genotypes (G1 to G7). In 2012, genotype 8 (G8) was identified in Croatia [42] and subsequently in Ukraine and Russia in 2013 [43]. In 2016, using BLV whole-genome sequences of G1, G2, G4 and G6 strains as well as 25 newly identified isolates from Peru, Bolivia and Paraguay, G9 was identified from Bolivian isolates by Polat et al. [44]. Interestingly, in the last several years, new genotypes have been identified in Asia. G10 was identified in Thailand in 2016 [45], Myanmar in 2017 [46] and China in 2018 [47]. Furthermore, G11 was identified in China in 2019 [48]. BLV whole-genome sequences are available from G1, G2, G4, G6, G9 and G10.

In addition to the global distribution of eleven genotypes, multiple genotypes are circulating in Western Europe, Asia and South America. Some countries in these regions with multiple detected genotypes such as America, Bulgaria, Japan and Argentina have a long history of BLV prevalence or high BLV prevalence rates [40,49,50,51,52,53,54,55], indicating that not only may the prolonged or delayed eradication process have resulted in the persistence of BLV but also allowed the introduction of new and multiple genotypic strains. This is also a serious concern with respect to the development of an efficacious BLV vaccine.

To date, only Zambian BLV isolates have been genotyped from Africa. Although high BLV prevalence has been reported in South Africa (Afrivet, 2018), there has been no molecular characterisation of South African BLV isolates. Therefore, this study was aimed to sequence BLV *env* and *gag* genes from South African isolates in order to investigate phylogenetic relationships and compare genetic variability of the South African isolates with other BLV isolates representing the eleven known genotypes from different geographical regions worldwide. Our analyses revealed that at least two genotypes, G1 and G4, are currently circulating in South Africa. Pairwise comparison and intragenotype evolutionary distances of full-length Env nucleotide and amino acid sequences showed that the G4 South African isolates are closely related to each other and their sequences are highly conserved. Furthermore, our analyses using full-length Gag nucleotide and amino acid sequences indicated that Gag sequences are generally more variable than Env sequences. Lastly, the alignment of BLV Env and Gag amino acid sequences identified genotype-specific as well as novel substitutions in the South African isolates.

## 2. Materials and Methods

### 2.1. Blood Samples and Extraction of Total Bovine Genomic DNA

Whole blood samples were obtained from a single herd of dairy cows in Port Elizabeth in South Africa in 2018. The blood samples were delivered to the University of Cape Town, South Africa for further analyses. Genomic DNA was extracted from whole blood samples (n = 8) using New MagNA Pure Compact Nucleic Acid Isolation Kits on the MagNA Pure Compact Instrument (Roche, Switzerland) as per the manufacturer’s instructions. The extracted DNA was stored at −20 °C until needed. The eight blood samples were designated sample IDs: K1170, K1194, M1878, M2746, P591, P2152, P2677 and L3401.

### 2.2. Amplification of BLV env Gene by Nested Polymerase Chain Reaction (nPCR) and Sanger Sequencing of Full-Length env Gene

**Figure 1:**
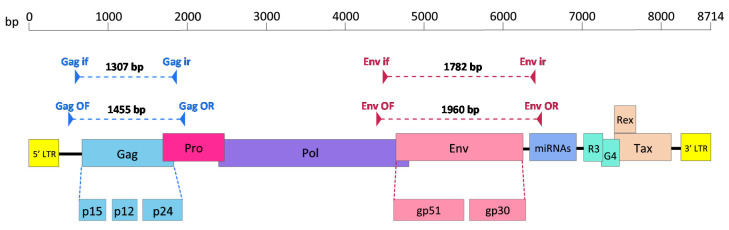
Schematic diagram showing open reading frames of the bovine leukaemia virus (BLV) genome and *gag* and *env* regions amplified using nested PCR (nPCR). Triangles denote primers and dotted lines denote amplified regions. Gag if, Gag inner forward primer; Gag ir, Gag inner reverse primer; Gag OF, Gag outer forward primer; Gag OR, Gag outer reverse primer; Env if, Env inner forward primer; Env ir, Env inner reverse primer; Env OF, Env outer forward primer; Env OR, Env outer reverse primer; Pro, protease; Pol, polymerase; Env, envelope; miRNAs, microRNAs; p15, matrix protein; p12, nucleocapsid protein; p24, capsid protein; gp51, surface protein; gp30, transmembrane protein. Adapted from Polat et al. (2017) [56].

A 1782 bp fragment containing the full-length BLV *env* gene was amplified from total bovine DNA extracted from the eight blood samples by nPCR using the following primers (Figure 1): Env OF (*O*uter *F*orward) (5′-CCTCCTACCAATTCTAAAGACC-3′), Env OR (*O*uter *R*everse) (5′-CACGCAGAAGCGACAATCTC-3′), Env if (*I*nner *F*orward) (5′-GGGCGGAGAAACACCYAAGG-3′) and Env ir (*I*nner *R*everse) (5′-CACTGACTATTCCACTAAGCC-3′). Both rounds of nPCR were performed in a 20 µL reaction mixture containing 1 µL of total genomic DNA or PCR product from the first round of nPCR, 1 µL of 2x KAPA 2G Robust HotStart ReadyMix (KAPA BIOSYSTEMS, Wilmington, MA, USA), 1 µL of each primer (10 µM each) and high-performance liquid chromatography (HPLC) water (Sigma-Aldrich, St Louis, MO, USA). The following thermocycling parameters were used: initial denaturation at 95 °C for 3 min followed by 25 cycles (30 cycles for the second round) of denaturation at 95 °C for 15 s, annealing at 55 °C (61 °C for the second round) for 15 s, extension at 72 °C for 2 min and final extension at 72 °C for 4 min.

PCR products were gel purified from 0.8% agarose TBE gels using the Zymoclean Gel DNA Recovery Kit (Zymo Research, Irvine, CA, USA) and sent to the Central Analytical Facility at the University of Stellenbosch (South Africa) for Sanger sequencing. The following primers were used: Env fwd1 (5′-AGGCGCTCTCCTGGCTAC-3′), Env fwd2 (5′-CARGTCTCCCAGATACAC-3′), Env fwd3 (5′-TGGGGATATGATCCCCTG-3′), Env fwd4 (5′-AATGTTTCTCAAGGCAAC-3′), Env fwd5 (5′-CCCAGAACCGACGGGGGC-3′), Env fwd6 (5′-AATGCTTGACCTCTCGCC-3′), Env rev1 (5′-ATCTGCCCCCACATAAGG-3′), Env rev2 (5′-GATGGTTTTGTTATATAC-3′), Env rev3 (5′-AGTCTCTGATGGCTAAGG-3′), Env rev4 (5′-GACTCTTTGCGAGAGAGG-3′) and Env rev5 (5′-ACAGCCTGGGGGTGCGTG-3′). The BLV *env* sequences from samples K1170, K1194, L3401, M1878, M2746, P2152, P2677 and P591 were deposited in the GenBank sequence database under the respective accession numbers MN966688, MN966689, MN966690, MN966691, MN966692, MN966693, MN966694, MN966695 (Supplementary Table S1).

### 2.3. Amplification of BLV gag Gene by nPCR and Sanger Sequencing of Full-Length gag Gene

A 1307 bp fragment containing the full-length BLV *gag* gene was amplified from the total bovine DNA extracted from the eight blood samples by nPCR using following primers (Figure 1): Gag OF (5′-GATTGATCACCCCGGAACCC-3′), Gag OR (5′-GTATTTTCAGCCCCGGTGTCC-3′), Gag if (5′-CTCTGGACCCACCCCCTCG-3′) and Gag ir (5′-CATTCTARTTCGGCCTCACTAAG-3′). Both rounds of nPCR were conducted in a 20 µL reaction mixture containing 5 µL of total genomic DNA or 1µL of the PCR product from the first round of nPCR, 1 µL of 2x KAPA 2G Robust HotStart ReadyMix (KAPA BIOSYSTEMS, Wilmington, MA, USA), 1 µL of each primer (10µM each) and high-performance liquid chromatography (HPLC) water (Sigma-Aldrich, St Louis, MO, USA). The following thermocycling parameters were used: initial denaturation at 95 °C for 3 min followed by 40 cycles (35 cycles for the second round) of denaturation at 95 °C for 15 s, annealing at 56 °C (61 °C for the second round) for 15 s, extension at 72 °C for 2 min and final extension at 72 °C for 4 min

PCR products were gel purified and sequenced as described earlier. The following primers were used: Gag fwd1 (5′-CCCTCGGCGGCRTTTTGG-3′), Gag fwd2 (5′-CCTYTGGCTTCAGGCCTG-3′), Gag fwd3 (5′-GACCATGCTATCGATGCC-3′), Gag rev1 (5′-GGGTTGTTCYTCKGGGGC-3′), Gag rev2 (5′-GRTTAAAACCCTGGAGGG-3′), Gag rev3 (5′-YCCCACCGGGGCGGCCAC-3′), Gag rev4 (5′-CCTCACTAAGRGRATCTG-3′). The BLV *gag* sequences from the samples K1170, K1194, L3401, M1878, M2746, P2152, P2677 and P591 were deposited in the GenBank sequence database under the respective accession numbers MN966696, MN966697, MN966698, MN966699, MN966700, MN966701, MN966702 and MN966703 (Supplementary Table S1).

### 2.4. Phylogenetic Analysis of BLV Full-Length and Partial env and Full-Length gag Nucleotide Sequences

The BLV *env* and *gag* nucleotide sequences of the eight South African isolates were assembled and aligned using CLC Main Workbench 7.9.3 (QIAGEN Bioinformatics, Copenhagen, Denmark). To select reference BLV nucleotide sequences for downstream analyses, 203 BLV full-length (1548 bp) *env*, 564 partial (444 bp) *env* and 113 full-length (1182 bp) *gag* nucleotide sequences with known genotypes were obtained from the Basic Local Alignment Search Tool (BLAST) database [57] using eight South African *env* and *gag* nucleotide sequences as query sequences; identical and highly similar sequences were removed using 98.5% sequence similarity as a threshold value in VSEARCH [58]. The selected sequences were further adjusted so that they represented isolates from a wide range of countries (Supplementary Table S2). The final sequences were aligned with the eight South African isolates using MUSCLE [59]. Model testing was performed to select the best evolutionary model based on the Bayesian information criterion (BIC) [60].

Neighbour-joining (NJ) and Maximum-likelihood (ML) trees based on the BLV full-length and 444 bp partial *env* nucleotide sequences were constructed using the Tamura-Nei substitution model with the Gamma distribution (TN83 + G) [61] and the Kimura-2-parameter model with the Gamma distribution (K2P +G) [62], respectively. NJ and ML trees based on the BLV full-length *gag* nucleotide sequences were constructed using the TN83 + G [61] model with the Gamma distribution (HKY + G) [63]. Nonparametric bootstrap analysis with 1000 iterations [64] was used to evaluate the robustness of evolutionary relationships. Alignment, model testing and tree building were performed using MEGA 7 [59].

### 2.5. Pairwise Comparison of BLV Env and Gag Nucleotide and Amino Acid Sequence

Pairwise evolutionary distances were estimated using the TN83 + G model for the BLV full-length *env* and *gag* nucleotide sequences and the K2P + G model for the partial *env* nucleotide sequences using MEGA7 [59]. Nonparametric bootstrap analysis with 1000 iterations [64] was used to evaluate the robustness of evolutionary relationships. For the full-length Env and partial Env sequences, the analysis was conducted only between South African isolates and selected G1 and G4 isolates from other geographic regions worldwide. 

To evaluate the nucleotide and amino acid sequence percent identity and differences, pairwise comparison of the BLV full-length Env, partial Env and full-length Gag sequences between the South African isolates and selected isolates from other geographic regions (46, 20 and 29 sequences, respectively) was performed using CLC Main Workbench 7.9.3 (QIAGEN Bioinformatics, Copenhagen, Denmark). For the partial Env sequences, sequence comparison was made only between South African isolates and selected G1 and G4 isolates from other geographic regions worldwide. 

### 2.6. Intragenotype and Intergenotype Evolutionary Distances of BLV Env Nucleotide and Amino Acid Sequences 

Two hundred and three full-length *env* nucleotide sequences of BLV isolates within nine known genotypes that were obtained from the BLAST database [57] in Section 2.4 were used for this analysis. Translated amino acid sequences were aligned using MUSCLE in MEGA7 [59] Mean intragenotype and intergenotype evolutionary distances of BLV Env nucleotide and amino acid sequences were estimated using the TN83 + G model [45] and the Jones–Taylor–Thornton model with the Gamma distribution (JTT + G) [49], respectively, using MEGA7 [59]. Nonparametric bootstrap analysis with 1000 iterations [64] was used to evaluate the robustness of evolutionary relationships.

### 2.7. Alignment of BLV Env and Gag Amino Acid Sequences 

To identify genotype-specific amino acid substitutions as well as those unique to the South African isolates, the full-length Env and Gag amino acid sequences of the South African isolates were aligned with 851 Env amino acid sequences of BLV isolates with eleven known genotypes and with 113 Gag amino acid sequences with six known genotypes (G1, G2, G4, G6, G9 and G10) obtained from the GenBank sequence database using MUSCLE in MEGA7 [59].

## 3. Results

### 3.1. Phylogenetic Analysis of BLV Full-Length env Nucleotide Sequences

**Figure 2:**
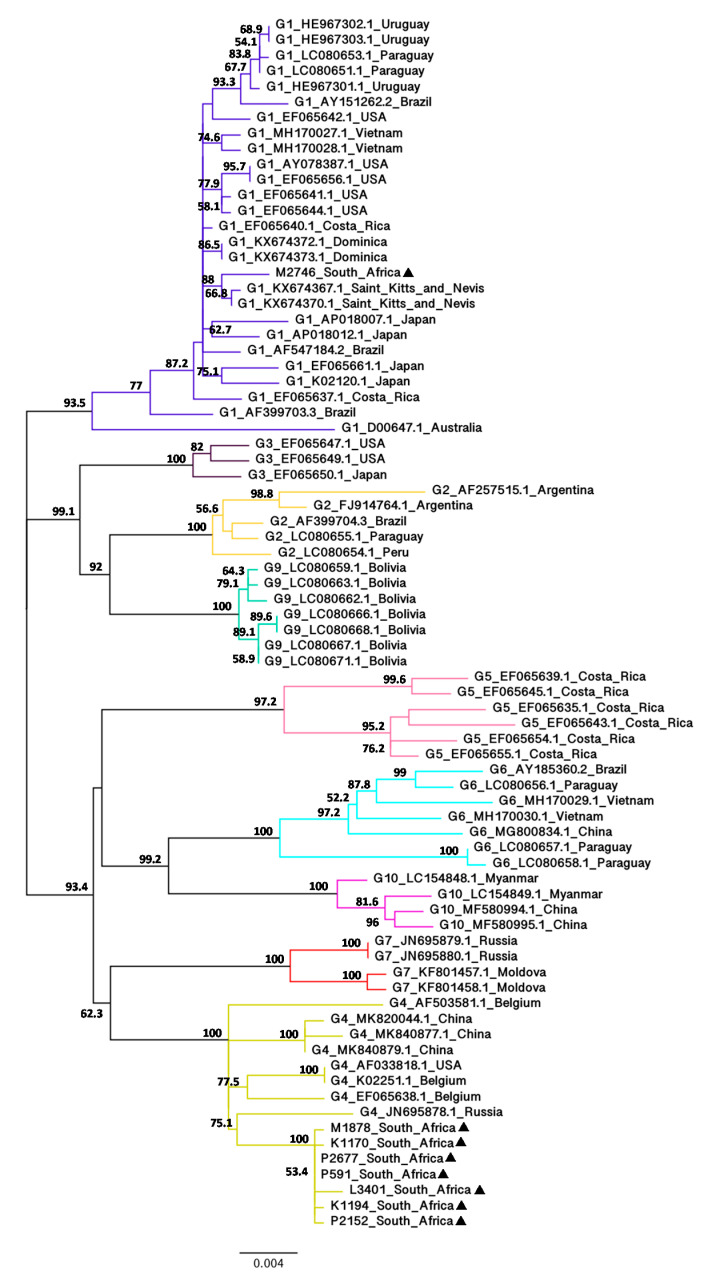
Maximum-likelihood phylogenetic tree based on the alignment of BLV full-length *env* nucleotide sequences (1548 bp) from South Africa and other geographic regions worldwide. The South African BLV isolates identified in this study are indicated by filled triangles (▲) following their sample ID. Other BLV strains are shown by genotype followed by GenBank accession number and country of origin. Numbers at the branches denote bootstrap support (1000 iterations). Bootstrap values of ≥ 50% are shown. The bar at the bottom of the figure denotes genetic distance.

The full-length *env* and *gag* sequences were successfully amplified and sequenced from total bovine DNA extracted from whole blood of eight BLV-infected cattle. To determine evolutionary relationships between the South African and BLV isolates from other geographic regions worldwide, neighbour-joining (NJ) (Supplementary Figure S1) and maximum-likelihood (ML) (Figure 2) phylogenetic trees based on the alignment of full-length (1548 bp) *env* nucleotide sequences were constructed using 70 BLV isolates representing G1 to G7, G9 and G10 from 18 countries. NJ and ML phylogenetic methods were used to validate these two results and both phylogenetic trees showed congruent topologies with high bootstrap values.

The NJ and ML trees revealed that seven of the South African isolates (K1170, K1194, M1878, P591, P2152, P2677 and L3401) are closely related to each other with 99.8% bootstrap value and all belong to G4. One South African isolate (M2746) did not cluster with the seven other South African isolates and grouped with G1. This phylogenetic analysis also indicated that the G4 South African isolates are more divergent from other G4 isolates, whereas the G1 South African isolate is less divergent from other G1 isolates. The G4 South African isolates appeared to be moderately related to a Russian isolate (JN695878.1) with 74.5% (NJ) and 75.1% (ML) bootstrap values and their estimated evolutionary divergences were 0.0132 (P2677 and P591), 0.0139 (K1170, K1194, M1878 and P2152) and 0.0153 (L3401) (Supplementary Figure S2). In contrast, the G1 South African isolate clustered with Saint Kitts and Nevis isolates (KX674367.1 and KX674370.1) with 90.5% (NJ) and 88% (ML) bootstrap values and their estimated evolutionary divergence was 0.0046 (KX674367.1) and 0.0039 (KX674370.1) (Supplementary Figure S2).

### 3.2. Phylogenetic Analysis of BLV Partial env Nucleotide Sequences (444 bp)

**Figure 3:**
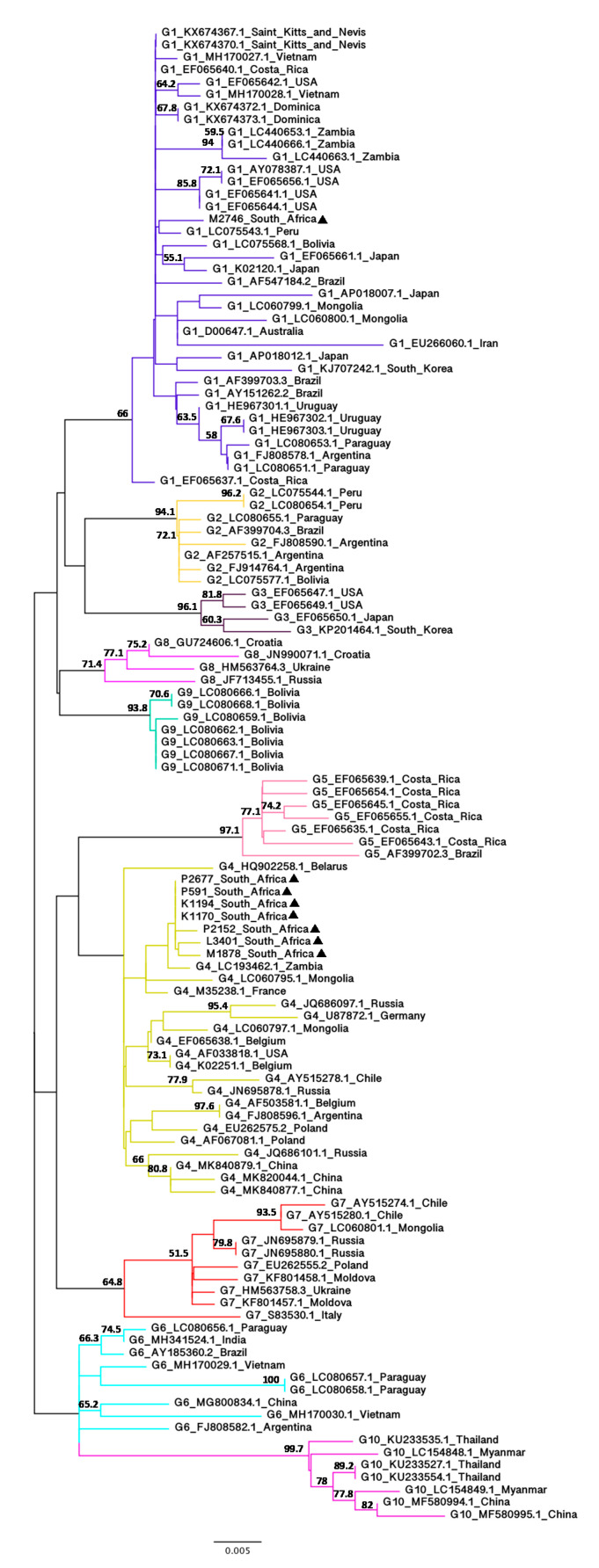
Maximum-likelihood phylogenetic tree based on the alignment of BLV partial *env* nucleotide sequences (444 bp) from South Africa and other geographic regions worldwide. The South African BLV strains identified in this study are indicated by filled triangles (▲) following their sample ID. Other strains are indicated by their genotype, GenBank accession number and country of origin. Numbers at the branches denote bootstrap support (1000 iterations). Bootstrap values of ≥50% are shown. The bar at the bottom of the figure denotes genetic distance.

The only African BLV sequences that have been sequenced are partial (444 bp) sequences of the *gp51* gene from Zambian isolates [65,66]. Furthermore, since the partial *env* sequences have been used for BLV genotyping in various studies [45,67,68,69,70,71,72], they are available from 10 genotypes from various geographic regions. To confirm the results obtained from the phylogenetic analyses based on the full-length *env* nucleotide sequences and to further investigate the phylogenetic relationships between the South African isolates with more diverse BLV isolates from different geographic regions, NJ (Supplementary Figure S3) and ML (Figure 3) trees based on the alignment of partial *env* nucleotide sequences were constructed using 112 BLV isolates from 32 countries. 

Despite the low to moderate bootstrap values, this second analysis was consistent with the first analysis using the full-length *env* nucleotide sequences, showing that seven of the South African isolates belong to G4 and one (M2746) belongs to G1. G2 sequences in the NJ tree clustered differently from the ML tree due to the difference in tree-building methods (distance-based vs. character-based methods) but the ML tree based on the partial *env* nucleotide sequences was congruent with the phylogenetic tree based on the full-length *env* nucleotide sequences. The seven G4 South African isolates clustered within the G4 group with 44% (NJ) and 44.2% (ML) bootstrap values whilst the South African isolate (M2746) clustered with other G1 isolates with 70.6% (NJ) and 66% (ML) bootstrap value. The G1 South African isolate did not cluster with three selected G1 Zambian isolates (LC440653.1, LC440663.1 and LC440666.1), which form a distinct monophyletic group as in Phiri’s study [66]. In contrast, despite low bootstrap values (32.5% for NJ and 36.4% for ML), the G4 South African isolates are more closely related to a G4 Zambian isolate (LC193462.1) than other G4 isolates. The estimated evolutionary distance between the seven G4 South African isolates and the G4 Zambian isolate was 0.0023 (K1170, K1194, P2677 and P591) and 0.0045 (L3401, M1878 and P2152) (Supplementary Figure S4).

### 3.3. Phylogenetic Analysis of BLV Full-Length gag Nucleotide Sequences

**Figure 4:**
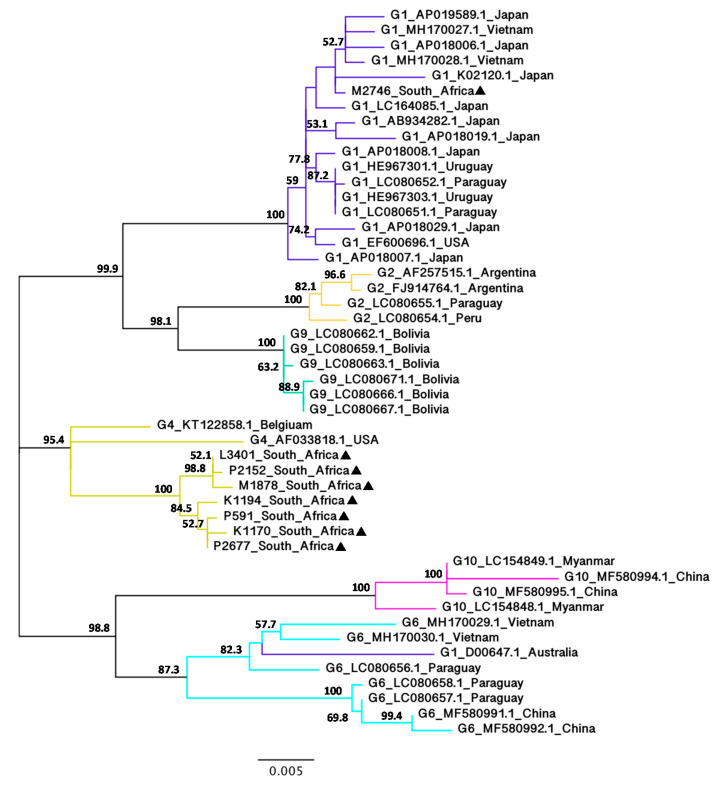
Maximum-likelihood phylogenetic tree based on the alignment of BLV full-length *gag* nucleotide sequences (1182 bp) from South Africa and other geographic regions worldwide. The South African BLV strains identified in this study are indicated by filled triangles (▲) following their sample ID. Other strains are indicated by their genotype, GenBank accession number and country of origin. Numbers at the branches denote bootstrap support (1000 iterations). Bootstrap values of ≥50% are shown. The bar at the bottom of the figure denotes genetic distance.

Although the BLV *gag* sequence has rarely been used for phylogenetic analyses, this was included here to add more insight into the genetic variabilities of the BLV isolates and to compare the results with the phylogenetic analyses of the BLV *env* nucleotide sequences. NJ (Supplementary Figure S5) and ML (Figure 4) trees based on the alignment of BLV full-length *gag* nucleotide sequences were constructed using 40 BLV isolates representing G1, G2, G4, G6, G9 and G10 from 11 countries.

Despite the limited number of full-length *gag* nucleotide sequences currently available on the GenBank sequence database, NJ and ML trees showed congruent topologies with the phylogenetic analyses of the BLV full-length *env* nucleotide sequences supported by high bootstrap values. Similar to the BLV *env* phylogenetic trees, the BLV *gag* trees showed that seven of the South African isolates belong to G4 with 90.4% (NJ) and 95.4% (ML) bootstrap values, whereas one South African isolate (M2746) belongs to G1 with 100% bootstrap value (NJ and ML). Interestingly, there appeared to be more genetic variability in the BLV *gag* nucleotide sequences amongst different genotypes as evident from the tree topology, as well as the pairwise evolutionary distance (Supplementary Figure S6). 

### 3.4. Pairwise Comparison of the BLV Full-Length env Sequences

**Figure 5:**
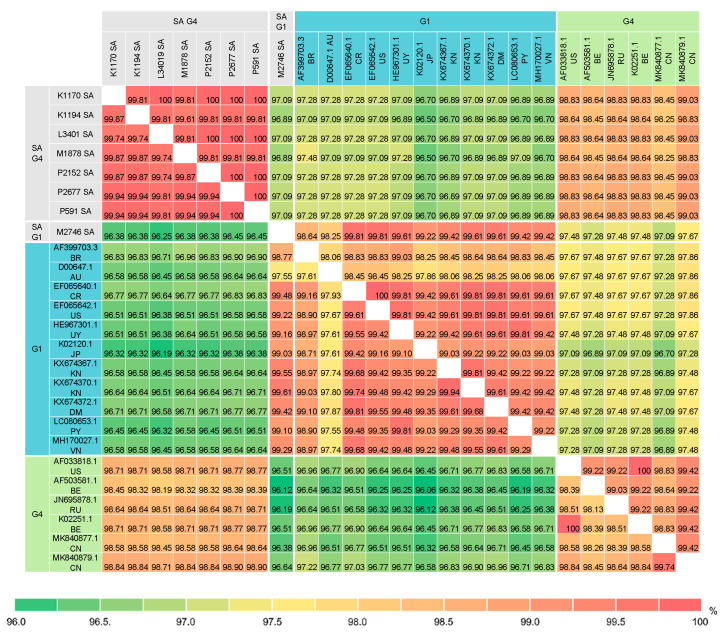
Pairwise percent identity of BLV full-length Env nucleotide (1548 bp) and amino acid (515 amino acids) sequences between G1 and G4 isolates from South Africa and other geographic regions worldwide. The lower matrix shows the percent identity of nucleotide sequences and the upper matrix shows that of amino acid sequences. The country of origin is indicated by 2-letter country codes (see Supplementary Table S2). SA, South Africa.

Pairwise comparison of the BLV full-length Env nucleotide and amino acid sequences between the South African isolates and 46 selected isolates (Supplementary Figures S7 and S8) were performed. Figure 5 shows pairwise percent identity of the BLV full-length Env sequences between the South African isolates and 17 selected G1 and G4 isolates. The analysis revealed that the G4 South African isolates shared significantly high sequence identity with each other: ≥99.74% nucleotide sequence identity (0–4 differences in 1548 nucleotides) and ≥99.81% amino acid sequence identity (0–2 differences in 515 amino acids) (Figure 5 and Supplementary Figure S8). In particular, the nucleotide sequences of the P2677 and P591isolates are identical. Furthermore, this analysis also showed that despite some nucleotide sequence variabilities (99.74–99.94% identity or 1–4 differences in 1548 nucleotides), five South African isolates K1170, P591, P2152, P2677 and L3401 shared 100% amino acid sequence identity, indicating that their nucleotide substitutions were synonymous mutations.

Consistent with the phylogenetic analyses based on the NJ and ML trees, the full-length Env nucleotide and amino acid sequences of the G1 South African isolate showed high sequence identity with the majority of the G1 isolates (Figure 5 and Supplementary Figure S8). The G1 South African full-length Env sequence shared 98.77–99.61% nucleotide sequence identity (6–19 differences in 1548 nucleotides) and 98.64–99.81% amino acid sequence identity (1–7 differences in 515 amino acids) with all G1 isolates except the Australian isolate (D00647.1). In contrast, there is slightly more sequence variability between the G4 South African isolates and other G4 isolates. The G4 South African isolates shared 98.19–98.90% nucleotide sequence identity (18–28 differences in 1548 nucleotides) and 98.25–99.03% amino acid sequence identity (5–9 differences in 515 amino acids) with all other G4 isolates.

### 3.5. Pairwise Comparison of BLV Partial Env Sequences

Pairwise comparison of the BLV partial Env nucleotide and amino acid sequences between the South African isolates and 20 selected G1 and G4 isolates (Supplementary Figures S9 and S10). Furthermore, in order to compare South African and Zambian isolates, all fourteen G1 Zambian sequences currently available on the GenBank sequence database were used in this analysis (Supplementary Tables S3 and S4). Supplementary Figure S11 shows alignment of the deduced amino acid sequences of the 148 amino acid region in the Env gp51 protein between the South African isolates and G1 and G4 isolates.

As opposed to the analyses based on the full-length Env sequences, sequences of the 444 bp region in the *gp51* gene appear to be slightly more variable as this region contains highly polymorphic epitope sequences and neutralising domains. The G1 South African sequence shared moderate sequence identity with the G1 isolates 97.30–99.55% nucleotide sequence identity (2–12 differences in 444 nucleotides) and 95.95–99.32% amino acid sequence identity (1–6 differences in 148 amino acids) (Supplementary Figures S9 and S10). The G4 South African isolates and G4 isolates showed 97.75–99.55% nucleotide sequence identity (2–10 differences in 444 nucleotides) and their amino acid sequences showed 95.95–99.32% (1–6 differences in 148 amino acids).

Comparison of the South African isolates with the Zambian isolates indicated that the G1 South African isolate is more similar to G1 isolates from other parts of the world than to the G1 Zambian isolates. The G1 South African nucleotide sequence is only moderately similar to the fourteen G1 Zambian isolates (Supplementary Table S3), showing 98.20–98.87% sequence identity or 5–8 differences in 444 nucleotides. However, their amino acid sequences shared relatively low sequence identity (96.62–98.65% identity or 2–5 differences in 148 amino acids). In contrast, the G4 South African isolates are more divergent from the other G4 isolates but highly similar to the G4 Zambian isolate. Three G4 South African isolates (M1878, P2677 and L3401) and four other G4 South African isolates (K1170, K1194, P591 and P2152) shared 99.55% (two nucleotide differences) and 99.77% nucleotide sequence identity (one nucleotide differences) with the Zambian isolate (LC193462), respectively (Supplementary Table S4). Furthermore, all G4 South African isolates except the M1878 isolate shared 100% amino acid sequence identity with the G4 Zambian isolate. The M1878 isolate shared 99.32% amino acid sequence identity with the Zambian isolate, which is only one amino acid difference between these isolates.

### 3.6. Pairwise Comparison of BLV Gag Sequences

**Figure 6:**
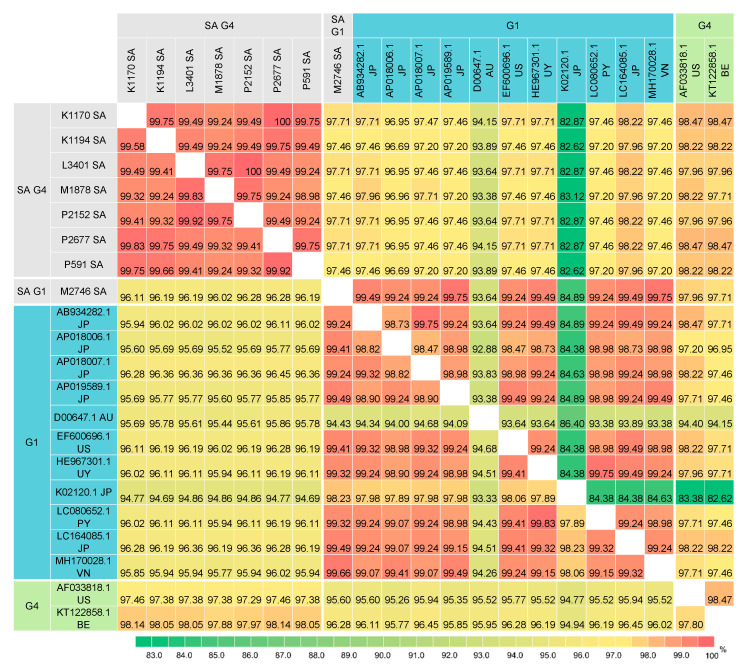
Pairwise percent identity of BLV full-length Gag nucleotide (1182 bp) and amino acid (393 amino acids) sequences between G1 and G4 isolates from South Africa and other geographic regions worldwide. The lower matrix shows the percent identity of nucleotide sequences and the upper matrix shows that of amino acid sequences. The country of origin is indicated by 2-letter country codes (see Supplementary Table S2). SA, South Africa.

Pairwise comparison of the BLV full-length Gag nucleotide and amino acid sequences between the South African isolates and 29 selected isolates from other geographic regions (Figure 6 and Supplementary Figures S12 and S13) showed that Gag sequences from different genotypes appeared to be more variable than Env sequences. Pairwise sequence identities of the full-length *env* nucleotide sequences were ≥94.44% (Figure 5 and Supplementary Figure S7) whereas those of the full-length *gag* nucleotide sequences were ≥92.58% (Figure 6 and Supplementary Figure S12).

Similarly, the Gag sequences amongst the G4 South African isolates appeared to be slightly more variable than their Env sequences. Whilst the full-length Env sequences of the G4 South African isolates shared high nucleotide and amino acid sequence identity (≥99.74% and ≥99.61%, respectively) with each other and Env amino acid sequences of the five G4 South African isolates are identical, their Gag sequences shared ≥99.24% nucleotide sequence identity (1–9 differences in 1182 nucleotides) and ≥98.98 amino acid sequence identity (0–4 differences in 393 amino acid) with each other (Supplementary Figures S12 and S13). The K1170 and P2677 isolates shared 100% Gag amino acid sequence identity, as did P2152 and L3401 isolates.

Only two G4 *gag* sequences have been published and these are from Belgium and America. Consistent with the phylogenetic analyses, this analysis showed that the G4 South African isolates are more divergent from the G4 Belgian (KT122858.1) and American (AF033818) isolates. The G4 South African isolates showed only 97.29–98.14% nucleotide identity and 97.71–98.47% amino acid identity with these two G4 isolates, which corresponds to 22–32 nucleotide differences and 6–9 amino acid differences.

In contrast to the G4 isolates, the G1 South African isolate shared relatively high sequence identity, 99.24–99.66% nucleotide sequence identity (4–9 differences in 1182 nucleotides) and 99.24–99.75% amino acid sequence identity (1–4 differences in 393 amino acids), with all G1 isolates except an Australian isolate (D000647) and a Japanese isolate (K02120.1). It should be noted that the Australian and Japanese isolates have a three-nucleotide deletion, equivalent to one amino acid deletion (which appears to result in an in-frame deletion) and they are more divergent from other G1 isolates.

### 3.7. Genetic Variabilities of BLV Env Sequences 

Using 203 full-length Env sequences with nine known genotypes obtained from the GenBank sequence database and the eight South African Env sequences, mean intragenotype and intergenotype nucleotide and amino acid distances were estimated (Supplementary Table S5). The analysis confirmed that the seven G4 South African isolates and one G1 South African isolate belong to G4 and G1, respectively, showing the lowest mean intergenotype distances. The mean nucleotide and amino acid distances between the G1 South Africa isolate and the G1 isolates were 0.0082 and 0.0063, respectively and those between the G4 South African isolates and the G4 isolates were 0.0138 and 0.0141, respectively. Furthermore, comparison of these mean intergenotype evolutionary distances indicated that the G1 South African isolate is less divergent from other G1 isolates whereas the G4 South African isolates are slightly more divergent from other G4 isolates, which also confirmed the results of the phylogenetic analyses based on the full-length *env* nucleotide sequences.

This analysis was also consistent with the results obtained from pairwise comparison of the full-length Env sequences, showing that the Env sequences of the G4 South African isolates themselves are significantly conserved. The mean intragenotype distances of the G4 South African Env sequences were 0.0133 and 0.0011 for nucleotide and amino acid sequences, respectively.

Furthermore, this analysis revealed that the Env sequences of G6 and G10 isolates are more variable than other genotypes. The G6 and G10 Env sequences showed mean intragenotype nucleotide distances of 0.0267 and 0.0273, respectively, and mean intragenotype amino acid distances of 0.0249 and 0.0320, respectively.

### 3.8. Alignment of BLV Env Amino Acid Sequences

To assess whether the nucleotide substitutions altered amino acid sequences in the South African isolates and to identify the genotype-specific amino acid substitutions previously described [41,44,45,73] as well as amino acid substitutions unique to the South African isolates, the full-length Env and Gag deduced amino acid sequences of the South African isolates were aligned with a total of the 851 Env amino sequences and 113 Gag amino acid sequences available from the GenBank sequence database. Table 1 shows the alignment of Env amino acid sequences of the South African isolates with those of the 43 selected isolates representing eleven genotypes.

Alignment of the BLV Env amino acid sequences confirmed the presence of the G1- and G4-specific amino acid substitutions in the South African isolates at fourteen residues (at positions 29, 48, 56, 73, 74, 82, 121, 132, 134, 144, 254, 479, 480 and 504) in their Env sequences. As some exceptions to these genotype-specific substitutions, a proline-to-alanine substitution at position 73 (P73A) in the L3401 isolate and a D134N substitution in the M1878 isolate were found. Both of these amino acid substitutions appeared to be G1-like and could be random mutations.

Furthermore, three unique amino acid substitutions in the Env sequences (at positions 59, 153 and 476) were found in some South African sequences. An isoleucine-to-leucine substitution at position 59 (I59L) and a histidine-to-glutamine substitution at position 153 (H153Q) were only present in the K1194 and M2746 isolates, respectively and thus, this could be a random mutation. The E476D substitution was consistently found in the seven G4 South African isolates. This substitution was also found in G1 Mexican sequences (KY548770.1, KY548786.1 and KY548787.1; data not shown) and a G9 Bolivian sequence (LC080675.1; data not shown). However, none of the (non-South African) G4 isolates had this substitution, suggesting that this amino acid substitution is unique to the G4 South African isolates.

### 3.9. Alignment of BLV Gag Amino Acid Sequences

Alignment of the Gag amino acid sequences of the South African isolates with 113 Gag amino acid sequences available on the GenBank sequence data also confirmed the presence of G1- and G4-specific amino acid substitutions in the South African isolates. Table 2 shows the alignment of Gag amino acid sequences of the South African isolates with those of the 21 selected isolates representing six genotypes. The analysis showed that the South African isolates contained genotype specific amino acid substitutions at four residues (at positions 63, 69, 88 and 365) in their Gag sequences. Furthermore, seven amino acid substitutions (positions 29, 107, 108, 278, 318, 341 and 343) in the Gag sequences that had not been previously described were detected in the South African isolates and these were mainly found in the G4 South African isolates.

Four of these seven amino acid substitutions were particularly unique to the South African sequences. The amino acid alignment showed that N29D, I278V and P343S substitutions were only found in the G4 South African sequences, though the I278V substitution was also detected in an Iranian isolate (LC193727.1; data not shown), which has not been genotyped. Although previously not described, our analysis indicates that the presence of methionine at position 318 in the M2746 sequence and isoleucine at the same position in the seven G4 South African sequences could be genotype-specific.

A K341Q substitution was only found in the South African P591 isolate amongst 113 isolates studied. In addition, a G1-like K69R substitution was found in the G4 South African M1878 isolate and a V108I substitution was only found in the M1878 isolate but not in other six G4 South African isolates. It is uncertain whether these amino acid substitutions are spontaneous mutations.

A genotype-specific glycine-to-glutamic acid substitution at position 88 (G88E) was previously described [44] and the presence of the glycine residue at this position was identified as G1-specific and the presence of the glutamic acid residue as G4-specific. However, in our study, the G4 South African isolates M1878, P2152 and L3401 had the glycine residue at this position. The A107V substitution in the K1194 isolate and an A107S substitution in the M1878, P2152 and L3401 isolates could be random mutations as these mutations did not appear to be genotype-specific.

## 4. Discussion

This study is the first known phylogenetic analysis of BLV isolates from South Africa. Full-length *env* and *gag* genes from eight South African BLV isolates were sequenced. Phylogenetic analyses based on these genes revealed that at least two genotypes, genotype 1 and genotype 4, are present in South Africa, which is consistent with the Zambian studies [65,66]. Phylogenetic analyses and pairwise comparison of Gag sequences further provided two findings. Firstly, the Env and Gag sequences of the G4 South African isolates are highly conserved and some of these G4 South African isolates encode identical amino acid sequences. Secondly, Env sequences are highly conserved within as well as between genotypes whereas Gag sequences are more variable between genotypes. Lastly, alignment of Env and Gag amino acid sequences identified genotype-specific as well as novel amino acid substitutions in the South African isolates.

In 2016, Pandey et al. identified a G4 isolate from a dairy cow in Zambia by phylogenetic analysis based on the 444 bp region of the *gp51* gene [65]. In 2018, using the same 444 bp region, they identified fourteen G1 isolates from beef cattle [66]. These Zambian studies enabled comparison of the South African and Zambian BLV partial Env sequences. This comparison revealed that the G1 South African isolate is more closely related to the majority of the G1 isolates (not from Zambia) than to the G1 Zambian isolates. This result was not surprising as Phiri et al. found in their 2018 study that the G1 Zambian isolates form a distinct group within G1 [66]. In contrast, the G4 South African isolates appeared to be highly similar to the G4 Zambian isolate. The G4 South African isolates differed from the G4 Zambian isolate only at one or two nucleotide positions; the amino acid sequence identity was 100% for six of the seven G4 South African isolates (K1170, K1194, P591, P2152, P2677 and L3401) and 99.32% for the M1878 isolate. However, we cannot entirely exclude the possibility that there are G1 South African isolates that are closely related to the G1 Zambian isolate as this was a very small study with samples only taken from a single herd. Similarly, only one G4 isolate was sequenced from Zambia and thus, there might be G4 Zambian isolates that are less similar to South African isolates. Furthermore, if future studies could sequence full-length *env* (1548 bp) and *gag* (1182 bp) genes from Zambian isolates as well as South African isolates, it would enable a more comprehensive analysis of the G1 and G4 isolates circulating in these two countries, as these comparisons are only based on a 444 bp region of *env*.

It should be noted that the collection dates are unknown for the majority of the available BLV sequences. Therefore, we could not perform the analysis and interpretation of the BLV evolutionary history for eleven genotypes as well as the G4 South African isolates.

Although *gag* sequences are rarely used for phylogenetic analysis and the number of *gag* sequences available on the GenBank sequence database is limited, we sequenced the full-length *gag* gene to compare the results obtained from phylogenetic analyses as well as sequence comparison and alignment of full-length and partial *env* sequences. Phylogenetic analyses based on the full-length *gag* nucleotide sequences were in agreement with the phylogenetic analyses based on the full-length and partial *env* nucleotide sequences, validating that one South African isolate belongs to G1 and the seven other South African isolates belong to G4. Consistent with the analyses based on the full-length Env sequences, Gag sequences between the G1 South African isolate and G1 isolates from other parts of the world appeared to be more conserved than those between the G4 South African isolates and G4 Belgian and American isolates.

Furthermore, full-length Gag sequences are slightly more variable between different genotypes than full-length Env sequences as evident from the phylogenetic analysis and pairwise sequence comparison. Similarly, whilst five out of the seven G4 South African Env amino acid sequences are identical, only two of their Gag sequences are identical. Since only 113 Gag sequences with known genotypes are available from a limited number of countries, obtaining more Gag sequences is desirable to validate these conclusions and will provide a more comprehensive understanding of evolutionary changes in the Gag sequences.

Amino acid sequence alignment between the South African isolates and global isolates representing all eleven genotypes detected seventeen and eleven amino acid substitutions in the South African Env and Gag sequences, respectively. These substitutions include genotype-specific substitutions as well as those unique to the South African isolates. The amino acid substitution at position E476D in the Env sequences as well as the amino acid substitutions at positions N29D, I278V and P343S in the Gag sequences were consistently detected in all G4 South African isolates. These substitutions are absent in other G4 isolates from other countries as well as any isolates from other genotypes. Importantly, these amino acid substitutions which are unique to the G4 South African isolates appear to be fairly conservative and thus, they are unlikely to cause functional changes to the proteins. No insertions and deletions were detected in the South African sequences.

The genotype-specific amino acid substitutions in the Env sequences detected in this study include those at position 29 in the signal sequence, positions 48, 73, 74 and 82 in the G epitope, position 56 in the H epitope, positions 134 and 144 in the second neutralising domain (ND2), position 254 in the D epitope and position 504 in the PXXP motif. The Env mutations in the South African isolates appeared to be concentrated in the conformational epitopes (F, G and H) and the ND2. These results are consistent with previous studies showing that the G epitopes, particularly residues 48, 74 and 82, are under positive selection and are the most polymorphic sites [51,73,74,75]. Homology modelling of the BLV Env protein has suggested that the conformational epitopes and the neutralising domains are located on the surface of the virus [14,73]. Therefore, it is not surprising that these epitopes and domains are targets of neutralising antibodies (NAbs) [16,18,19,24] and under strong selection pressure [73], leading to the antigenic variations in these regions [19].

There are three types of conformational epitopes (F, G and H) [17,76,77] on the Env gp51 surface protein which are important for cell fusion and syncytium formation during viral dissemination [16]. In our study, four point mutations in the G epitopes (positions 48, 73, 74 and 82) and one point mutation in the H epitope (position 56) were detected from the South African isolates. Since functional studies on the Env protein and Env mutants are scarce, functional consequences of the Env mutations found in our studies are unknown. However, non-conservative mutations in the conformational epitopes and the neutralising domain 2 (ND2) would affect the recognition of NAbs to these epitopes. A previous study which identified the S56F substitution in the H epitope showed that this substitution altered the recognition of a monoclonal antibody to this epitope owing to the drastic change from the small serine residue to the large hydrophobic phenylalanine residue [78]. This effect would also apply to the S82F substitution in the G epitope. Mutations in the conformational epitopes appear to benefit viral persistence through antigenic changes and immune escape. Previous studies demonstrated that BLV natural variants lacking one or two of the conformational epitopes were viable and appear to have evolved these mutations to evade host immune responses by altered antibody recognition [19,78]. However, BLV mutants lacking all conformational epitopes have not been detected and these deletion mutants retained at least one of the three conformational epitopes [19,78]. These observations indicate that the simultaneous loss of these epitopes would be deleterious and possibly cause loss of infectivity to the virus [16].

The BLV Env protein contains three neutralising domains (ND1 to ND3) [24] and these domains also play a role in viral infectivity (cell fusion and syncytium) as well as immunogenicity [15,24,79]. Previous studies demonstrated that the ND2 is particularly prone to multiple point mutations [41,46] but it was also shown that ND2 is under purifying selection [73,75], reflecting its functional significance. Rodríguez et al. [41] identified eleven point mutations within the ND2 from BLV strains from a variety of geographic regions. In contrast, the ND1 and ND3 appear to be less prone to mutations [41,80]. Using homology modelling, Moratorio et al. [79] demonstrated that the D134N substitution in the ND2 changed net charge in a loop of the Env protein, possibly disturbing the immunogenicity and/or fusogenic properties of the ND2. One G4 South African isolate (M1878) had the G1-like D134N substitution in this ND2. It is unclear whether this is a spontaneous mutation.

Eleven amino acid substitutions were found in the South African Gag sequences and the majority of the substitutions (six out of eleven substitutions) occurred in the p15 matrix (MA) protein (positions 26, 63, 69, 88, 107 and 108) whereas only two amino acid substitutions were found in the p24 capsid (CA) protein (positions 278 and 318). Since the CA protein is the second major target for the NAbs next to the gp51 surface protein [32], this protein could be under selection pressure. Yet, it is possible that mutations in the CA could render a fitness cost to the virus and cause deleterious effects to viral replication. A genetic footprinting analysis on the Moloney murine leukaemia virus demonstrated that the majority of the MA regions were tolerant of insertions whereas the N-terminal region of the CA was susceptible to insertions [81]. They further demonstrated that although Gag mutants harbouring mutations in the MA and N-terminal region of the CA all produced virions, CA mutants produced an abundance of immature virions [81]. Furthermore, whilst the MA mutants showed reverse transcriptase (RT) and nuclear transport activities and appeared infectious, the RT and nuclear transport activities were not detected in the CA mutants, suggesting the mutations affect uncoating or viral entry [81].

Three amino acid substitutions were detected in the p12 nucleocapsid (NC) protein (positions 341, 343 and 365). The retroviral NC protein contains conserved zinc-finger motifs [82,83,84,85] and basic residues [86,87,88] for RNA packaging and virion assembly [89,90]. The K341Q substitution in the NC protein was only detected from the South African P591 isolate, not from any other South African or global isolates. This mutation might be a spontaneous mutation. However, this amino acid change may cause functional changes in the virion assembly. Wang et al. [91] performed alanine-scanning mutagenesis study for the basic residues in the BLV NC and demonstrated that although the K341A substitution in the NC did not affect RNA packaging, the K341A mutant had two- to three-fold reduction in virion production. Although lysine can be substituted with other polar amino acids including glutamine, it is unknown whether the loss of charge may also affect virion production.

This study served as the first phylogenetic analysis of the BLV full-length *env* and *gag* sequences from eight South African isolates. Since the sample population was small, and was only collected from a single herd, and there was only one G1 isolate in our study, future studies will be required to obtain more samples from different herds and geographic regions to investigate whether other BLV genotypes are circulating in cattle in South Africa. Needless to say, if more BLV sequences from Africa become available, it would provide greater insight into BLV genotypes currently circulating in Africa. Lastly, the detection of two genotypes (G1 and G4) in southern Africa highlights the urgent need for disease management and the development of an efficacious vaccine against local strains.

## Figures and Tables

**Table 1:** Amino acid substitutions in the BLV Env protein of the South African sequences and 43 selected sequences representing eleven genotypes.

		gp51													gp30			
Epitopes and Motifs		Leader Sequence	G Epitope	H Epitope		G Epitope				ND2				D Epitope				PXXP Motif
Amino acid position		29	48	56	59	73	74	82	121	132	134	144	153	254	476	479	480	504
References		d	a,d	a		a,b	a,d	a,d	a,c	a	a,d	a,d		a,d		d	d	d
SA G1	M2746	R	A	S	I	A	K	S	R	Q	D	I	Q	S	E	L	T	V
G1	AF399703.3 BR	Q	A	S	I	A	K	F	R	Q	N	I	H	S	E	S	T	V
	AP018021.1 JP	R	A	S	I	A	K	S	R	Q	D	I	H	S	E	L	T	V
	EF065641.1 US	R	A	S	I	A	K	S	R	Q	D	I	H	S	E	L	T	V
	EF065646.1 JP	Q	A	S	I	A	K	S	R	Q	D	I	H	S	E	L	T	V
	HE967301.1 UY	R	A	S	I	A	K	S	R	Q	N	I	H	S	E	L	T	V
	KX674639.1 KN	R	A	S	I	A	K	S	R	Q	D	I	H	S	E	L	T	V
	LC440653.1 ZM	−	−	−	−	−	−	−	R	Q	D	I	H	−	−	−	−	−
	LC075558.1 PY	−	−	−	−	−	−	−	R	Q	D	I	H	−	−	−	−	−
G2	AF257515.1 AR	Q	A	S	I	A	K	F	R	Q	D	I	H	L	E	L	A	T
	LC080654.1 PE	Q	V	S	I	A	K	F	R	Q	D	I	H	L	E	F	A	T
	LC080655.1 PY	Q	A	S	I	A	K	F	R	Q	D	I	H	L	E	F	A	T
G3	EF065647.1 US	Q	A	S	I	A	K	F	R	Q	D	I	H	L	E	F	A	T
	EF065650.1 JP	Q	A	S	I	A	K	F	R	Q	D	I	H	L	E	F	A	T
	JQ686091.1 RU	Q	T	S	I	P	R	F	H	Q	D	I	H	L	E	F	P	T
SA G4	K1170	Q	T	F	I	P	R	F	H	R	D	T	H	L	D	F	P	T
	K1194	Q	T	F	L	P	R	F	H	R	D	T	H	L	D	F	P	T
	L3401	Q	T	F	I	A	R	F	H	R	D	T	H	L	D	F	P	T
	M1878	Q	T	F	I	P	R	F	H	R	N	T	H	L	D	F	P	T
	P2152	Q	T	F	I	P	R	F	H	R	D	T	H	L	D	F	P	T
	P2677	Q	T	F	I	P	R	F	H	R	D	T	H	L	D	F	P	T
	P591	Q	T	F	I	P	R	F	H	R	D	T	H	L	D	F	P	T
G4	AF033818.1 US	Q	T	S	I	P	R	F	H	Q	D	I	H	L	E	F	P	T
	AF067081.1 PL	−	−	−	−	−	−	−	H	Q	D	I	H	−	−	−	−	−
	AY515279.1 CL	Q	T	S	I	P	R	F	H	Q	D	I	H	L	E	F	P	S
	JN695878.1 RU	Q	T	S	I	P	R	F	H	Q	D	I	H	L	E	F	P	T
	KF801460.2 MD	Q	T	S	I	P	R	F	H	Q	D	I	H	L	E	F	P	T
	LC193462.1 ZM	−	−	−	−	−	−	−	H	R	D	T	H	−	−	−	−	−
	M35238.1 FR	Q	T	F	I	P	R	F	H	Q	D	T	H	L	E	F	P	T
	M35240.1 BE	Q	T	S	I	P	R	F	H	Q	D	I	H	L	E	F	P	T
	MK820044.1 CN	Q	T	S	I	P	R	F	H	Q	D	I	H	L	E	F	P	T
G5	AF399702.3 BR	Q	T	S	I	A	R	F	R	R	D	I	H	L	−	−	−	−
	EF065635.1 CR	R	T	S	I	A	R	F	R	R	D	I	H	L	E	F	T	T
	EF065636.1 CR	R	T	S	I	A	R	F	R	R	D	I	H	L	E	L	T	V
G6	AY185360.2 BR	Q	T	S	I	A	R	F	R	Q	D	T	H	L	E	F	T	T
	LC075576.1 BO	Q	T	S	I	A	R	F	R	Q	D	T	H	L	E	F	T	T
	LC080656.1 PY	Q	T	S	I	A	R	F	R	Q	D	T	H	L	E	F	T	T
	MF580991.1 CN	Q	T	S	I	A	R	F	R	Q	D	T	Y	L	E	F	T	T
	KU233530.1 TH	−	−	−	−	−	−	−	R	Q	D	T	H	−	−	−	−	−
G7	KF801457.1.MD	Q	I	S	I	A	R	F	R	Q	D	I	H	L	E	F	T	A
	JN695879.1 RU	Q	T	S	I	A	R	F	R	Q	D	I	H	L	E	F	T	A
	S83530.1 IT	−	−	−	−	−	−	−	R	Q	D	I	H	−	−	−	−	−
G8	GU724606.1 HR	−	−	−	−	−	−	−	R	Q	D	I	H	−	−	−	−	−
	JF713455.1 RU	−	−	−	−	−	−	−	R	Q	D	I	H	−	−	−	−	−
	LT970927.1 IT	−	−	−	−	−	−	−	R	Q	D	I	H	−	−	−	−	−
G9	LC080659.1 BO	Q	A	S	I	A	K	L	R	Q	D	I	H	L	E	F	A	T
	LC080664.1 BO	Q	A	S	I	A	K	F	R	Q	D	I	H	L	E	F	A	T
G10	KU233527.1 TH	−	−	−	−	−	−	−	H	Q	D	T	H	−	−	−	−	−
	LC154848.1 MM	Q	T	S	I	A	R	F	R	Q	D	T	H	L	E	F	T	T
	MF580994.1 CN	Q	T	S	I	A	R	F	H	Q	D	T	H	L	E	F	T	T
G11	KU764746.1 CN	−	−	−	−	−	−	−	R	Q	D	I	H	−	−	−	−	−

a, amino acid substitutions described by Rodriguez et al. (2009) [41]; b, genotype-specific amino acid substitution described by Zhao and Buehring (2007) [73]; c, genotype-specific amino acid substitution previously described by Lee et al. (2016) [45]; d, genotype-specific amino acid substitutions described by Polat et al. (2016) [44]. SU, surface protein; TM, transmembrane protein; ND 2, neutralising domain 2; PXXP motif, proline-rich motif for the cell signalling; Dash (–) indicates amino acid residues are not available due to the partial sequences. Amino acid substitutions that were exclusively found in the South African isolates are shown in red font. Each amino acid has been assigned a unique background color. The country of origin is indicated by 2-letter codes (see Supplementary Table S2).

**Table 2:** Amino acid substitutions in the BLV Gag protein of the South African sequences and 21 selected sequences representing six genotypes.

		p15 MA						p24 CA		p12 NC		
Amino Acid Position		29	63	69	88	107	108	278	318	341	343	365
References			a	a	a							a
SA G1	M2746	N	T	K	G	A	V	I	M	K	P	A
G1	AP018021.1 JP	N	T	R	G	A	V	I	M	K	P	A
	D00647.1 AU	N	A	K	E	A	V	I	T	K	P	T
	AB934282.1 JP	N	T	R	G	A	I	I	M	K	P	A
	LC005615.1 JP	N	T	R	G	A	V	I	M	K	P	A
	LC080651.1 PY	N	T	R	G	A	V	I	M	K	P	A
	MH170027.1 VN	N	T	R	G	A	V	I	M	K	P	A
	MH170028.1 VN	N	T	R	G	A	V	I	M	K	P	A
	EF600696.1 US	N	T	R	G	A	V	I	V	K	P	A
	HE967301.1 UY	N	T	R	G	A	V	I	M	K	P	A
G2	FJ914764.1 AR	N	T	R	G	A	V	I	I	K	P	T
	LC080654.1 PE	N	T	R	G	A	V	I	I	K	P	T
	LC080655.1 PY	N	T	R	G	A	V	I	I	K	P	T
SA G4	K1170	D	A	K	E	A	V	V	I	K	S	T
	K1194	D	A	K	E	V	V	V	I	K	S	T
	L3401	D	A	K	G	S	V	V	I	K	S	T
	M1878	D	A	R	G	S	I	V	I	K	S	T
	P2152	D	A	K	G	S	V	V	I	K	S	T
	P2677	D	A	K	E	A	V	V	I	K	S	T
	P591	D	A	K	E	A	V	V	I	Q	S	T
G4	KT122858.1 BE	N	A	K	E	A	V	I	I	K	P	T
	AF033818.1 US	N	A	K	E	A	I	I	V	K	P	T
G6	MF580991.1 CN	N	V	K	E	V	V	I	T	K	P	T
	LC080656.1 PY	N	V	K	E	A	V	I	T	K	P	T
	MH170030.1 VN	N	V	K	E	A	I	I	T	K	P	T
G9	LC080659.1 BO	N	T	R	G	A	V	I	I	K	P	T
	LC080664.1 BO	N	T	R	G	A	V	I	I	K	P	T
G10	MF580994.1 CN	N	V	K	E	A	D	I	V	K	P	A
	LC154848.1 MM	N	V	K	E	A	V	I	V	K	P	A

a, genotype-specific amino acid substitutions previously described by Polat et al. (2016) [56]. Amino acid substitutions that are previously not described are highlighted in blue. G1 and G4 sequences are highlighted in yellow for comparison. MA, matrix; CA, capsid; NC, nucleocapsid. The country of origin is indicated by 2-letter codes Dash (–) indicates amino acid residues are not available due to the partial sequences. Amino acid substitutions that were exclusively found in the South African isolates are shown in red font. Each amino acid has been assigned a unique background color. The country of origin is indicated by 2-letter codes (see Supplementary Table S2).

## Supplementary Materials

The following are available online at https://www.mdpi.com/1999-4915/12/8/898/s1, Figure S1: Neighbour-joining phylogenetic tree based on the alignment of BLV full-length env nucleotide sequences (1548 bp) from South Africa and other geographic regions worldwide. Figure S2: Pairwise evolutionary distance of BLV full-length *env* nucleotide sequences (1548 bp) from the South African isolates and other G1 and G4 isolates from other geographic regions worldwide. Figure S3: Neighbour-joining phylogenetic tree based on the alignment of BLV partial *env* nucleotide sequences (444 bp) from South Africa and other geographic regions worldwide. Figure S4: Pairwise evolutionary distance of BLV partial *env* nucleotide sequences (444 bp) from the South African isolates and other G1 and G4 isolates from other geographic regions worldwide. Figure S5: Neighbour-joining phylogenetic tree based on the alignment of BLV full-length *gag* nucleotide sequences (1182 bp) from South Africa and other geographic regions worldwide. Figure S6: Pairwise evolutionary distance of BLV full-length *gag* nucleotide sequences (1182 bp) from the South African isolates and 29 isolates from other geographic regions worldwide. Figure S7: Pairwise percent identity of BLV full-length Env nucleotide (1548 bp) and amino acid sequences (515 amino acids) between the South African isolates and 46 isolates from other geographic regions worldwide. Figure S8: Pairwise comparison of BLV full-length Env nucleotide (1548 bp) and amino acid (515 amino acids) differences between the South Africa isolates and 46 isolates from other geographic regions worldwide. Figure S9: Pairwise percent identity of the BLV partial Env nucleotide (444 bp) and amino acid (148 amino acids) sequences between the South African isolates and G1 and G4 isolates from other geographic regions worldwide. Figure S10: Pairwise comparison of BLV partial Env nucleotide (444 bp) and amino acid (148 amino acids) differences between the South Africa isolates and G1 and G4 isolates from other geographic regions worldwide. Figure S11: Comparison of 148 amino acid region in the Env gp51 protein between the G1 and G4 South African and Zambian isolates. Figure S12: Pairwise percent identity of BLV full-length Gag nucleotide (1182 bp) and amino acid (393 amino acids) sequences between the South African isolates and 29 isolates from other geographic regions worldwide. Figure S13: Pairwise comparison of BLV full-length Gag nucleotide (1182 bp) and amino acid (393 amino acids) differences between the South Africa isolates and 29 isolates from other geographic regions worldwide. Table S1: GenBank accession numbers, sample IDs and genotypes of the BLV full-length *env* and *gag* nucleotide sequences of the South African isolates used in this study. Table S2: GenBank accession numbers, the country of origin and genotypes of the BLV full-length *env*, partial *env* and full length *gag* nucleotide sequences used in this study. Table S3: Pairwise comparison of BLV partial Env nucleotide and amino acid sequences between G1 South African and Zambian sequences. Table S4: Pairwise comparison of BLV partial Env nucleotide and amino acid sequences between G4 South African and Zambian sequences. Table S5: Mean nucleotide and amino acid distances in the BLV full-length Env sequences within (intragenotype) and between (intergenotype) BLV strains from South Africa and other geographic regions worldwide.
