# Supplementary material for: Phylogenetic Analysis of South African Bovine Leukaemia Virus (BLV) Isolates
Source: Viruses. 2020 Aug 17;12(8):898. doi: 10.3390/v12080898 (PMC7472093; doi:10.3390/v12080898)
Supplement: Supplementary file 1 [file viruses-12-00898-s001.pdf]

Table S1. GenBank accession numbers, sample IDs and genotypes of the BLV full-length *env* and *gag* nucleotide sequences of the South African isolates used in this study.

Gene	GenBank accession number	Sample ID	Genotype
env	MN966688.1	K1170	4
	MN966689.1	K1194	4
	MN966690.1	L3401	4
	MN966691.1	M1878	4
	MN966692.1	M2746	1
	MN966693.1	P2152	4
	MN966694.1	P2677	4
	MN966695.1	P591	4
gag	MN966696.1	K1170	4
	MN966697.1	K1194	4
	MN966698.1	L3401	4
	MN966699.1	M1878	4
	MN966700.1	M2746	1
	MN966701.1	P2152	4
	MN966702.1	P2677	4
	MN966703.1	P591	4

Table S2. GenBank accession numbers, country of origin (2-letter country code), genotypes and phylogenetic analyses conducted for each BLV isolate in this study.

Genotype	GenBank accession number	Country of origin	Phylogenetic analyses	References
1	AB934282.1	Japan (JP)	*gag*	Direct submission by Mekata and Norimine (2014)
	AF399703.3	Brazil (BR)	Full-length *env*, partial *env*	[1]
	AF547184.2	Brazil (BR)	Full-length *env*, partial *env*	[2]
	AP018006.1	Japan (JP)	*gag*	[3]
	AP018007.1	Japan (JP)	Full-length *env*, partial *env*, *gag*	[3]
	AP018008.1	Japan (JP)	gag	[3]
	AP018012.1	Japan (JP)	Full-length *env*, partial *env*	[3]
	AP018019.1	Japan (JP)	*gag*	[3]
	AP018029.1	Japan (JP)	*gag*	[3]
	AP019589.1	Japan (JP)	*gag*	Direct submission by Murakami and Sato (2019)
	AY078387.1	USA (US)	Full-length *env*, partial *env*	[4]
	AY151262.2	Brazil (BR)	Full-length *env*, partial *env*	Direct submission by Stancek et al. (2002)
	D00647.1	Australia (AU)	Full-length *env*, partial *env*, *gag*	[5]
	EF065637.1	Costa Rica (CR)	Full-length *env*, partial *env*	[6]
	EF065640.1	Costa Rica (CR)	Full-length *env*, partial *env*	[6]
	EF065641.1	USA (US)	Full-length *env*, partial *env*	[6]
	EF065642.1	USA (US)	Full-length *env*, partial *env*	[6]
	EF065644.1	USA (US)	Full-length *env*, partial *env*	[6]
	EF065656.1	USA (US)	Full-length *env*, partial *env*	[6]
	EF065661.1	Japan (JP)	Full-length *env*, partial *env*	[6]
	EF600696.1	USA (US)	*gag*	[7]
	EU266060.1	Iran (IR)	Partial *env*	[8]
	FJ808578.1	Argentina (AR)	Partial *env*	[9]
	HE967301.1	Uruguay (UY)	Full-length *env*, partial *env*, *gag*	Direct submission by Cristina (2012)
	HE967302.1	Uruguay (UY)	Full-length *env*	Direct submission by Cristina (2012)
	HE967303.1	Uruguay (UY)	Full-length *env*, partial *env*, *gag*	Direct submission by Cristina (2012)
	K02120.1	Japan (JP)	Full-length *env*, partial *env*, *gag*	[10]
	KJ707242.1	South Korea (KR)	Partial *env*	Direct submission by Kim (2014)
	KX674367.1	Saint Kitts and Nevis (KN)	Full-length *env*, partial *env*	[11]

	KX674370.1	Saint Kitts and Nevis (KN)	Full-length *env*, partial *env*	[11]
	KX674372.1	Dominica (DM)	Full-length *env*, partial *env*	[11]
	KX674373.1	Dominica (DM)	Full-length *env*, partial *env*	[11]
	LC060799.1	Mongolia (MN)	Partial *env*	[12]
	LC060800.1	Mongolia (MN)	Partial *env*	[12]
	LC075543.1	Peru (PE)	Partial *env*	[13]
	LC075568.1	Bolivia (BO)	Partial *env*	[13]
	LC080651.1	Paraguay (PY)	Full-length *env*, partial *env*, *gag*	[13]
	LC080652.1	Paraguay (PY)	*gag*	[13]
	LC080653.1	Paraguay (PY)	Full-length *env*, partial *env*	[13]
	LC164085.1	Japan (JP)	*gag*	[14]
	LC440653.1	Zambia (ZM)	Partial *env*	[15]
	LC440663.1	Zambia (ZM)	Partial *env*	[15]
	LC440666.1	Zambia (ZM)	Partial *env*	[15]
	MH170027.1	Vietnam (VN)	Full-length *env*, partial *env*	[16]
	MH170028.1	Vietnam (VN)	Full-length *env*, partial *env*	[16]
2	AF257515.1	Argentina (AR)	Full-length *env*, partial *env*, *gag*	[17]
	AF399704.3	Brazil (BR)	Full-length *env*, partial *env*	[1]
	FJ808590.1	Argentina (AR)	Partial *env*	[9]
	FJ914764.1	Argentina (AR)	Full-length *env*, partial *env*, *gag*	[18]
	LC075544.1	Peru (PE)	Partial *env*	[13]
	LC075577.1	Bolivia (BO)	Partial *env*	[13]
	LC080654.1	Peru (PE)	Full-length *env*, partial *env*, *gag*	[13]
	LC080655.1	Paraguay (PY)	Full-length *env*, partial *env*, *gag*	[13]
3	EF065647.1	USA (US)	Full-length *env*, partial *env*	[6]
	EF065649.1	USA (US)	Full-length *env*, partial *env*	[6]
	EF065650.1	Japan (JP)	Full-length *env*, partial *env*	[6]
	KP201464.1	South Korea (KR)	Partial *env*	[19]
4	AF033818.1	USA (US)	Full-length *env*, partial *env*, *gag*	Direct submission by Chappey (1997)
	AF067081.1	Poland (PL)	Partial *env*	Direct submission by Mikiewicz et al. (1998)
	AF503581.1	Belgium (BE)	Full-length *env*, partial *env*	[20]
	AY515278.1	Chile (CL)	Partial *env*	[21]
	EF065638.1	Belgium (BE)	Full-length *env*, partial *env*	[6]

	EU262575.2	Poland (PL)	Partial *env*	Direct submission by Kuzmak et al. (2007)
	FJ808596.1	Argentina (AR)	Partial *env*	[9]
	HQ902258.1	Belarus (BY)	Partial *env*	[22]
	JN695878.1	Russia (RU)	Full-length *env*, partial *env*	Direct submission by Lomakina et al. (2011)
	JQ686097.1	Russia (RU)	Partial *env*	Direct submission by Lomakina et al. (2011)
	JQ686101.1	Russia (RU)	Partial *env*	Direct submission by Lomakina et al. (2012)
	K02251.1	Belgium (BE)	Full-length *env*, partial *env*	[23]
	KT122858.1	Belgium (BE)	*gag*	Direct submission by Rosewick et al. (2015)
	LC060795.1	Mongolia (MN)	Partial *env*	[12]
	LC060797.1	Mongolia (MN)	Partial *env*	[12]
	LC193462.1	Zambia (ZM)	Partial *env*	[24]
	M35238.1	France (FR)	Partial *env*	[25]
	MK820044.1	China (CN)	Full-length *env*, partial *env*	[26]
	MK840877.1	China (CN)	Full-length *env*, partial *env*	[26]
	MK840879.1	China (CN)	Full-length *env*, partial *env*	[26]
	U87872.1	Germany (GE)	Partial *env*	[27]
5	AF399702.3	Brazil (BR)	Partial *env*	[1]
	EF065635.1	Costa Rica (CR)	Full-length *env*, partial *env*	[6]
	EF065639.1	Costa Rica (CR)	Full-length *env*, partial *env*	[6]
	EF065643.1	Costa Rica (CR)	Full-length *env*, partial *env*	[6]
	EF065645.1	Costa Rica (CR)	Full-length *env*, partial *env*	[6]
	EF065654.1	Costa Rica (CR)	Full-length *env*, partial *env*	[6]
	EF065655.1	Costa Rica (CR)	Full-length *env*, partial *env*	[6]
6	AY185360.2	Brazil (BR)	Full-length *env*, partial *env*	[2]
	FJ808582.1	Argentina (AR)	Partial *env*	[9]
	LC080656.1	Paraguay (PY)	Full-length *env*, partial *env*, *gag*	[13]
	LC080657.1	Paraguay (PY)	Full-length *env*, partial *env*, *gag*	[13]
	LC080658.1	Paraguay (PY)	Full-length *env*, partial *env*, *gag*	[13]
	MH341524.1	India (IN)	Partial *env*	[28]
	MF580991.1	China (CN)	*gag*	[29]
	MF580992.1	China (CN)	*gag*	[29]
	MG800834.1	China (CN)	Full-length *env*, partial *env*	[30]
	MH170029.1	Vietnam (VN)	Full-length *env*, partial *env*, *gag*	[16]

	MH170030.1	Vietnam (VN)	Full-length *env*, partial *env*, *gag*	[16]
7	AY515274.1	Chile (CL)	Partial *env*	[21]
	AY515280.1	Chile (CL)	Partial *env*	[21]
	EU262555.2	Poland (PL)	Partial *env*	Direct submission by Kuzmak and Olech. (2010)
	HM563758.3	Ukraine (UA)	Partial *env*	Direct submission by Kuzmak and Olech. (2010)
	JN695879.1	Russia (RU)	Full-length *env*, partial *env*	Direct submission by Lomakina et al (2012)
	JN695880.1	Russia (RU)	Full-length *env*, partial *env*	Direct submission by Lomakina et al (2012)
	KF801457.1	Moldova (MD)	Full-length *env*, partial *env*	[31]
	KF801458.1	Moldova (MD)	Full-length *env*, partial *env*	[31]
	LC060801.1	Mongolia (MN)	Partial *env*	[12]
	S83530.1	Italy (IT)	Partial *env*	[32]
8	GU724606.1	Croatia (HR)	Partial *env*	[33]
	HM563764.3	Ukraine (UA)	Partial *env*	[22]
	JF713455.1	Russia (RU)	Partial *env*	Direct submission by Shaeva et al. (2011)
	JN990071.1	Croatia (HR)	Partial *env*	[33]
9	LC080659.1	Bolivia (BO)	Full-length *env*, partial *env*, *gag*	[13]
	LC080662.1	Bolivia (BO)	Full-length *env*, partial *env*, *gag*	[13]
	LC080663.1	Bolivia (BO)	Full-length *env*, partial *env*, *gag*	[13]
	LC080666.1	Bolivia (BO)	Full-length *env*, partial *env*, *gag*	[13]
	LC080667.1	Bolivia (BO)	Full-length *env*, partial *env*, *gag*	[13]
	LC080668.1	Bolivia (BO)	Full-length *env*, partial *env*	[13]
	LC080671.1	Bolivia (BO)	Full-length *env*, partial *env*, *gag*	[13]
10	KU233527.1	Thailand (TH)	Partial *env*	[34]
	KU233535.1	Thailand (TH)	Partial *env*	[34]
	KU233554.1	Thailand (TH)	Partial *env*	[34]
	LC154848.1	Myanmar (MM)	Full-length *env*, partial *env*, *gag*	[35]
	LC154849.1	Myanmar (MM)	Full-length *env*, partial *env*, *gag*	[35]
	MF580994.1	China (CN)	Full-length *env*, partial *env*, *gag*	[29]
	MF580995.1	China (CN)	Full-length *env*, partial *env*, *gag*	[29]

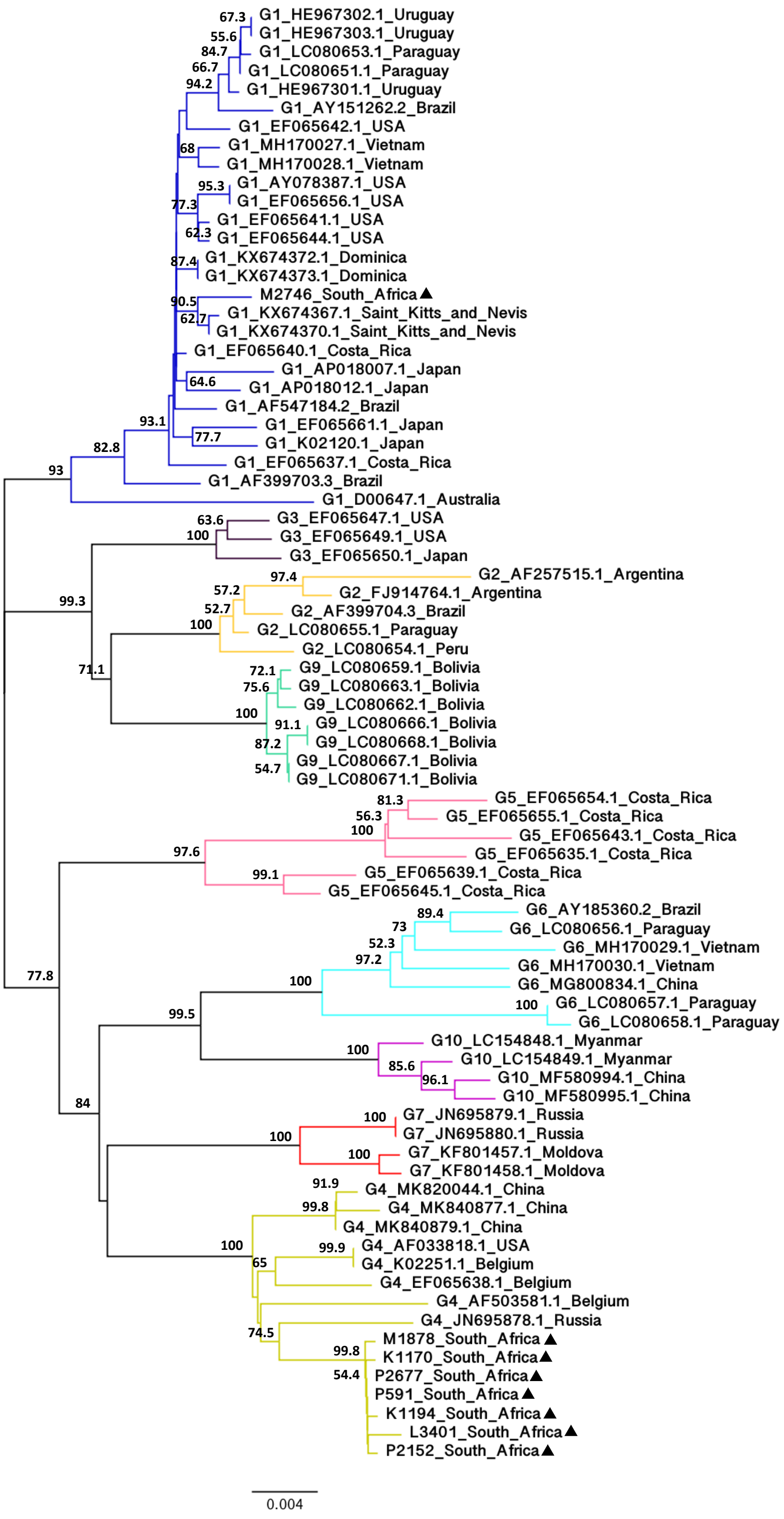

Figure S1. Neighbour-joining phylogenetic tree based on the alignment of BLV full-length *env* nucleotide sequences (1,548 bp) from South Africa and other geographic regions worldwide. The South African BLV isolates identified in this study are indicated by filled triangles (▲) followed by their sample ID. Other BLV strains are shown by genotype followed by GenBank accession number and country of origin. Numbers at the branches denote bootstrap support (1000 iterations). Bootstrap values of ≥50% are shown. The bar at the bottom of the figure denotes genetic distance.

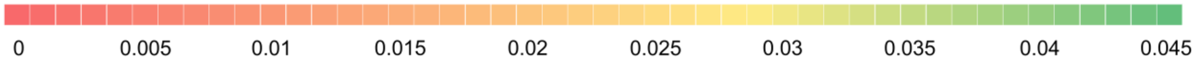

Group	Sequence	K1170 SA	K1194 SA	L34019 SA	M1878 SA	P2152 SA	P2677 SA	P591 SA	M2746 SA	AF399703.3 BR	D00647.1 AU	EF065640.1 CR	EF065642.1 US	HE967301.1 UY	K02120.1 JP	KX674367.1 KN	KX674370.1 KN	KX674372.1 DM	LC080653.1 PY	MH170027.1 VN	AF033818.1 US	AF503581.1 BE	JN695878.1 RU	K02251.1 BE	MK840877.1 CN
SA G4	K1170 SA																								
	K1194 SA	0.0013																							
	L3401 SA	0.0026	0.0026																						
	M1878 SA	0.0013	0.0013	0.0026																					
	P2152 SA	0.0013	0.0013	0.0026	0.0013																				
	P2677 SA	0.0006	0.0006	0.0019	0.0006	0.0006																			
	P591 SA	0.0006	0.0006	0.0019	0.0006	0.0006	0.0000																		
SA G1	M2746 SA	0.0387	0.0387	0.0402	0.0387	0.0387	0.0380	0.0380																	
G1	AF399703.3 BR	0.0336	0.0336	0.0351	0.0321	0.0336	0.0329	0.0329	0.0125																
	D00647.1 AU	0.0365	0.0365	0.0380	0.0365	0.0365	0.0358	0.0358	0.0256	0.0249															
	EF065640.1 CR	0.0343	0.0343	0.0358	0.0343	0.0343	0.0336	0.0336	0.0052	0.0085	0.0214														
	EF065642.1 US	0.0373	0.0373	0.0388	0.0373	0.0373	0.0366	0.0366	0.0079	0.0112	0.0242	0.0039													
	HE967301.1 UY	0.0373	0.0373	0.0388	0.0358	0.0373	0.0366	0.0366	0.0085	0.0105	0.0249	0.0046	0.0059												
	K02120.1 JP	0.0394	0.0393	0.0408	0.0394	0.0393	0.0386	0.0386	0.0098	0.0132	0.0248	0.0059	0.0085	0.0092											
	KX674367.1 KN	0.0366	0.0365	0.0380	0.0366	0.0365	0.0358	0.0358	0.0046	0.0105	0.0235	0.0032	0.0059	0.0066	0.0078										
	KX674370.1 KN	0.0358	0.0358	0.0373	0.0358	0.0358	0.0351	0.0351	0.0039	0.0099	0.0228	0.0026	0.0052	0.0059	0.0072	0.0006									
	KX674372.1 DM	0.0351	0.0351	0.0366	0.0351	0.0351	0.0344	0.0344	0.0059	0.0092	0.0221	0.0019	0.0046	0.0052	0.0065	0.0039	0.0032								
	LC080653.1 PY	0.0380	0.0380	0.0395	0.0365	0.0380	0.0373	0.0373	0.0092	0.0112	0.0256	0.0052	0.0065	0.0019	0.0098	0.0072	0.0065	0.0059							
	MH170027.1 VN	0.0366	0.0365	0.0380	0.0366	0.0365	0.0358	0.0358	0.0072	0.0105	0.0235	0.0032	0.0059	0.0052	0.0078	0.0052	0.0046	0.0039	0.0072						
G4	AF033818.1 US	0.0132	0.0132	0.0146	0.0132	0.0132	0.0126	0.0126	0.0372	0.0321	0.0344	0.0329	0.0358	0.0358	0.0379	0.0351	0.0343	0.0336	0.0365	0.0351					
	AF503581.1 BE	0.0160	0.0173	0.0187	0.0174	0.0173	0.0167	0.0167	0.0417	0.0359	0.0396	0.0373	0.0403	0.0403	0.0423	0.0395	0.0388	0.0381	0.0410	0.0395	0.0166				
	JN695878.1 RU	0.0139	0.0139	0.0153	0.0139	0.0139	0.0132	0.0132	0.0410	0.0358	0.0372	0.0365	0.0395	0.0395	0.0416	0.0388	0.0381	0.0373	0.0403	0.0388	0.0153	0.0194			
	K02251.1 BE	0.0132	0.0132	0.0146	0.0132	0.0132	0.0126	0.0126	0.0372	0.0321	0.0344	0.0329	0.0358	0.0358	0.0379	0.0351	0.0343	0.0336	0.0365	0.0351	0.0000	0.0166	0.0153		
	MK840877.1 CN	0.0145	0.0145	0.0159	0.0145	0.0145	0.0139	0.0139	0.0386	0.0320	0.0371	0.0342	0.0371	0.0371	0.0392	0.0364	0.0357	0.0350	0.0379	0.0364	0.0146	0.0180	0.0166	0.0146	
	MK840879.1 CN	0.0119	0.0119	0.0132	0.0119	0.0119	0.0112	0.0112	0.0357	0.0292	0.0343	0.0314	0.0343	0.0343	0.0364	0.0336	0.0329	0.0321	0.0350	0.0336	0.0119	0.0160	0.0139	0.0119	0.0026
		SA G4							SA G1	G1											G4				

Figure S2. Pairwise evolutionary distance of BLV full-length *env* nucleotide sequences (1,548 bp) between G1 and G4 isolates from South Africa and other geographic regions worldwide. The values indicate the number of base substitutions per site between sequences. The country of origin is indicated by 2-letter country codes (See Table S2). SA, South Africa.

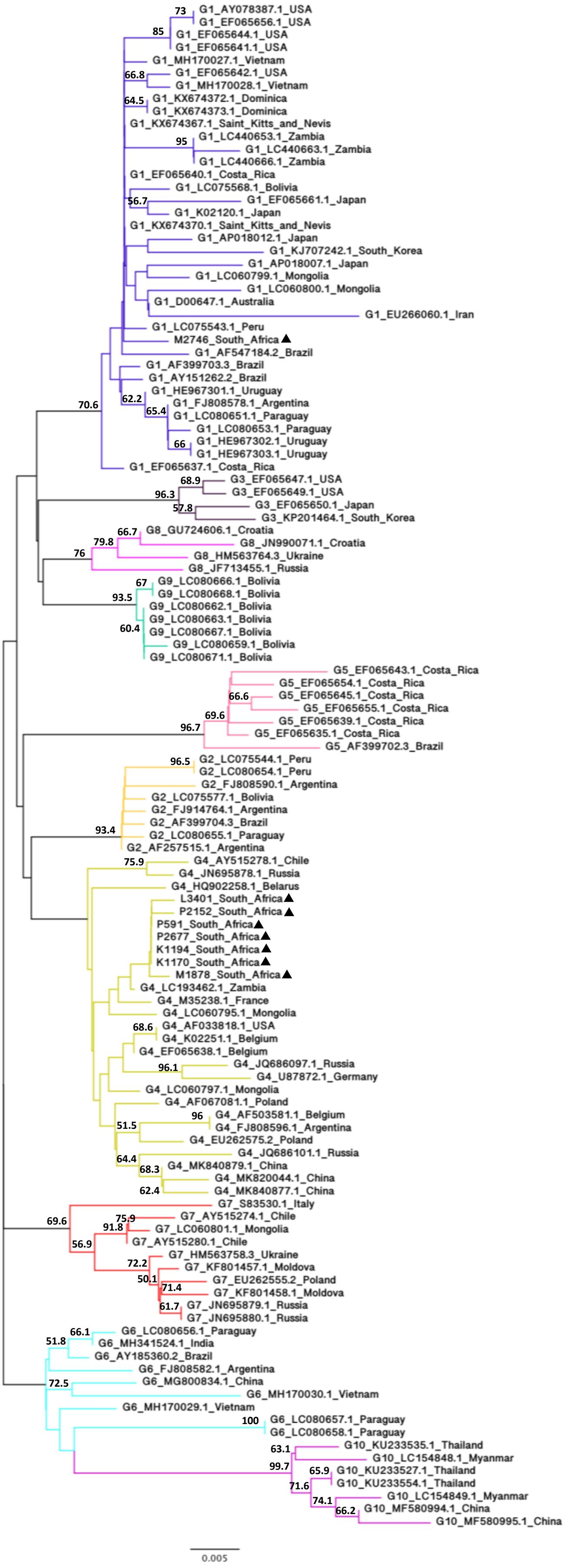

Figure S3. Neighbour-joining phylogenetic tree based on the alignment of BLV partial *env* nucleotide sequences (444 bp) from South Africa and other geographic regions worldwide. The South African BLV strains identified in this study are indicated by filled triangles (▲) followed by their sample ID. Other strains are indicated by their genotype, GenBank accession number and country of origin. Numbers at the branches denote bootstrap support (1000 iterations). Bootstrap values of ≥50% are shown. The bar at the bottom of the figure denotes genetic distance.

0 0.005 0.01 0.015 0.02 0.025 0.03 0.035 0.04 0.045 0.05 0.055 0.06

		K1170 SA	K1194 SA	L3401 SA	M1878 SA	P2152 SA	P2677 SA	P591 SA	M2746 SA	AP018007.1 JP	EU266060.1 IR	FJ808578.1 AR	HE967302.1 UY	KJ707242.1 KR	KX674367.1 KN	KX674372.1 DM	LC075543.1 PE	LC075568.1 BO	LC080653.1 PY	LC440653.1 ZM	LC440663.1 ZM	LC440666.1 ZM	AF067081.1 PL	AY515278.1 CL	EU262575.2 PL	FJ808596.1 AR	HQ902258.1 BY	JQ686101.1 RU	LC060795.1 MN	LC193462.1 ZM	M35238.1 FR
SA G4	K1170 SA																														
	K1194 SA	0.0000																													
	L3401 SA	0.0023	0.0023																												
	M1878 SA	0.0023	0.0023	0.0045																											
	P2152 SA	0.0023	0.0023	0.0045	0.0045																										
	P2677 SA	0.0000	0.0000	0.0023	0.0023	0.0023																									
	P591 SA	0.0000	0.0000	0.0023	0.0023	0.0023	0.0000																								
SA G1	M2746 SA	0.0310	0.0310	0.0335	0.0335	0.0334	0.0310	0.0310																							
G1	AP018007.1 JP	0.0387	0.0387	0.0413	0.0413	0.0411	0.0387	0.0387	0.0162																						
	EU266060.1 IR	0.0491	0.0491	0.0518	0.0518	0.0516	0.0491	0.0491	0.0282	0.0358																					
	FJ808578.1 AR	0.0310	0.0310	0.0335	0.0284	0.0334	0.0310	0.0310	0.0114	0.0186	0.0258																				
	HE967302.1 UY	0.0284	0.0284	0.0310	0.0260	0.0308	0.0284	0.0284	0.0138	0.0210	0.0282	0.0023																			
	KJ707242.1 KR	0.0384	0.0384	0.0410	0.0410	0.0408	0.0384	0.0384	0.0185	0.0257	0.0381	0.0209	0.0233																		
	KX674367.1 KN	0.0261	0.0261	0.0286	0.0286	0.0284	0.0261	0.0261	0.0045	0.0115	0.0234	0.0068	0.0091	0.0138																	
	KX674372.1 DM	0.0286	0.0286	0.0311	0.0311	0.0310	0.0286	0.0286	0.0068	0.0138	0.0259	0.0091	0.0115	0.0161	0.0023																
	LC075543.1 PE	0.0286	0.0286	0.0311	0.0311	0.0310	0.0286	0.0286	0.0068	0.0138	0.0259	0.0091	0.0115	0.0161	0.0023	0.0045															
	LC075568.1 BO	0.0311	0.0311	0.0337	0.0337	0.0335	0.0311	0.0311	0.0091	0.0162	0.0283	0.0115	0.0138	0.0185	0.0045	0.0068	0.0068														
	LC080653.1 PY	0.0335	0.0335	0.0361	0.0310	0.0359	0.0335	0.0335	0.0138	0.0210	0.0282	0.0023	0.0045	0.0233	0.0091	0.0115	0.0115	0.0138													
	LC440653.1 ZM	0.0335	0.0335	0.0361	0.0361	0.0359	0.0335	0.0335	0.0114	0.0186	0.0307	0.0138	0.0162	0.0209	0.0068	0.0091	0.0091	0.0115	0.0162												
	LC440663.1 ZM	0.0385	0.0385	0.0411	0.0411	0.0410	0.0385	0.0385	0.0161	0.0233	0.0357	0.0185	0.0209	0.0257	0.0114	0.0138	0.0138	0.0162	0.0209	0.0045											
	LC440666.1 ZM	0.0335	0.0335	0.0361	0.0361	0.0359	0.0335	0.0335	0.0114	0.0186	0.0307	0.0138	0.0162	0.0209	0.0068	0.0091	0.0091	0.0115	0.0162	0.0000	0.0045										
G4	AF067081.1 PL	0.0092	0.0092	0.0115	0.0115	0.0115	0.0092	0.0092	0.0310	0.0387	0.0491	0.0310	0.0284	0.0384	0.0261	0.0236	0.0286	0.0311	0.0335	0.0335	0.0385	0.0335									
	AY515278.1 CL	0.0187	0.0187	0.0211	0.0211	0.0210	0.0187	0.0187	0.0310	0.0361	0.0491	0.0310	0.0284	0.0410	0.0261	0.0286	0.0236	0.0311	0.0335	0.0335	0.0385	0.0335	0.0187								
	EU262575.2 PL	0.0115	0.0115	0.0138	0.0138	0.0092	0.0115	0.0115	0.0334	0.0411	0.0516	0.0334	0.0308	0.0408	0.0284	0.0310	0.0310	0.0335	0.0359	0.0334	0.0384	0.0334	0.0115	0.0210							
	FJ808596.1 AR	0.0139	0.0139	0.0163	0.0163	0.0162	0.0139	0.0139	0.0361	0.0439	0.0545	0.0361	0.0335	0.0436	0.0311	0.0337	0.0337	0.0362	0.0387	0.0387	0.0437	0.0387	0.0139	0.0236	0.0115						
	HQ902258.1 BY	0.0139	0.0139	0.0163	0.0163	0.0162	0.0139	0.0139	0.0310	0.0387	0.0491	0.0310	0.0284	0.0410	0.0261	0.0286	0.0286	0.0311	0.0335	0.0335	0.0385	0.0335	0.0139	0.0187	0.0162	0.0187					
	JQ686101.1 RU	0.0162	0.0162	0.0186	0.0186	0.0185	0.0162	0.0162	0.0384	0.0462	0.0568	0.0384	0.0358	0.0459	0.0334	0.0359	0.0359	0.0385	0.0410	0.0410	0.0461	0.0410	0.0162	0.0259	0.0185	0.0210	0.0210				
	LC060795.1 MN	0.0092	0.0092	0.0115	0.0115	0.0115	0.0092	0.0092	0.0310	0.0387	0.0491	0.0310	0.0284	0.0410	0.0261	0.0286	0.0286	0.0311	0.0335	0.0335	0.0385	0.0335	0.0139	0.0187	0.0162	0.0187	0.0139	0.0210			
	LC193462.1 ZM	0.0023	0.0023	0.0045	0.0045	0.0045	0.0023	0.0023	0.0284	0.0361	0.0464	0.0284	0.0260	0.0384	0.0236	0.0261	0.0261	0.0286	0.0310	0.0310	0.0359	0.0310	0.0115	0.0163	0.0138	0.0163	0.0115	0.0186	0.0068		
	M35238.1 FR	0.0045	0.0045	0.0068	0.0068	0.0068	0.0045	0.0045	0.0310	0.0387	0.0491	0.0310	0.0284	0.0384	0.0261	0.0286	0.0286	0.0261	0.0335	0.0335	0.0385	0.0335	0.0092	0.0187	0.0115	0.0139	0.0139	0.0162	0.0092	0.0068	
	U87872.1 DE	0.0210	0.0210	0.0235	0.0235	0.0234	0.0210	0.0210	0.0385	0.0464	0.0570	0.0385	0.0359	0.0434	0.0335	0.0361	0.0361	0.0387	0.0411	0.0359	0.0410	0.0359	0.0210	0.0260	0.0234	0.0260	0.0260	0.0282	0.0260	0.0235	0.0210
		SA G4							SA G1	G1													G4								

Figure S4. Pairwise evolutionary distance of BLV partial *env* nucleotide sequences (444 bp) between G1 and G4 isolates from South Africa and other geographic regions worldwide. The values indicate the number of base substitutions per site between sequences. The country of origin is indicated by 2-letter country codes (See Table S2). SA, South Africa.

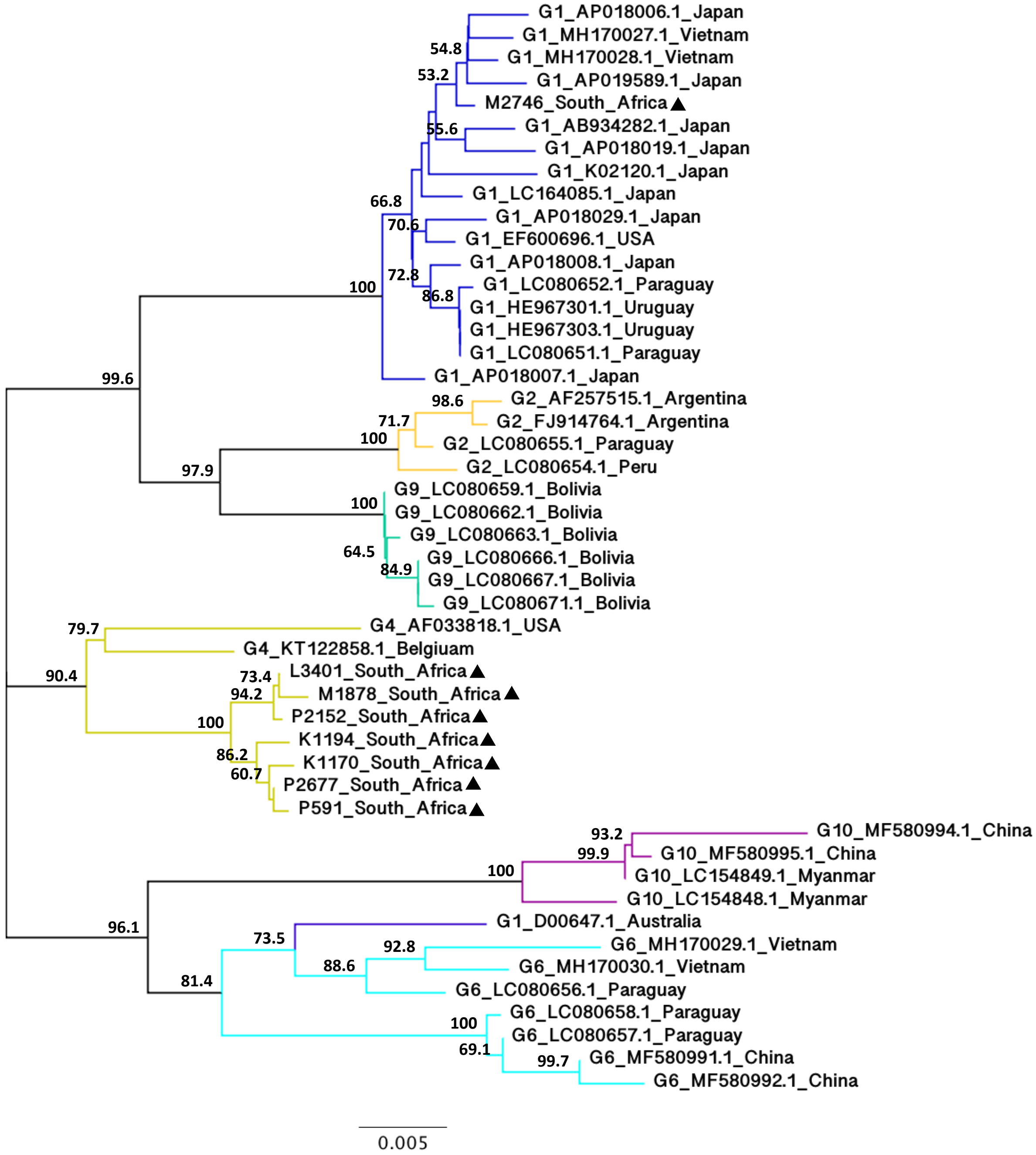

Figure S5. Neighbour-joining phylogenetic tree based on the alignment of BLV full-length *gag* nucleotide sequences (1,182 bp) from South Africa and other geographic regions worldwide. The South African BLV strains identified in this study are indicated by filled triangles (▲) followed by their sample ID. Other strains are indicated by their genotype, GenBank accession number and country of origin. Numbers at the branches denote bootstrap support (1000 iterations). Bootstrap values of ≥50% are shown. The bar at the bottom of the figure denotes genetic distance.

0 0.005 0.01 0.015 0.02 0.025 0.03 0.035 0.04 0.045 0.05 0.055 0.06 0.065 0.07 0.075 0.08

Group	Isolate	K1170 SA	K1194 SA	L3401 SA	M1878 SA	P2152 SA	P2677 SA	P591 SA	M2746 SA	AB934282.1 JP	AP018006.1 JP	AP018007.1 JP	AP019589.1 JP	D00647.1 AU	EF600696.1 US	HE967301.1 UY	K02120.1 JP	LC080652.1 PY	LC164085.1 JP	MH170028.1 VN	AF257515.1 AR	FJ914764.1 AR	LC080654.1 PE	LC080655.1 PY	AF033818.1 US	KT122858.1 BE	LC080656.1 PY	LC080658.1 PY	MF580992.1 CN	MH170029.1 VN	LC080662.1 BO	LC080663.1 BO	LC080667.1 BO	LC080671.1 BO	LC154848.1 MM	LC154849.1 MM	MF580994.1 CN
SA G4	K1170 SA																																				
	K1194 SA	0.0043																																			
	L3401 SA	0.0052	0.0060																																		
	M1878 SA	0.0069	0.0078	0.0017																																	
	P2152 SA	0.0060	0.0069	0.0009	0.0026																																
	P2677 SA	0.0017	0.0026	0.0052	0.0069	0.0060																															
	P591 SA	0.0026	0.0034	0.0060	0.0078	0.0069	0.0009																														
SA G1	M2746 SA	0.0424	0.0414	0.0414	0.0434	0.0404	0.0404	0.0413																													
G1	AB934282.1 JP	0.0445	0.0435	0.0435	0.0435	0.0435	0.0425	0.0435	0.0078																												
	AP018006.1 JP	0.0475	0.0464	0.0464	0.0485	0.0464	0.0454	0.0464	0.0052	0.0114																											
	AP018007.1 JP	0.0404	0.0393	0.0393	0.0393	0.0393	0.0384	0.0393	0.0078	0.0069	0.0114																										
	AP019589.1 JP	0.0475	0.0465	0.0464	0.0485	0.0464	0.0455	0.0464	0.0052	0.0114	0.0070	0.0114																									
	D00647.1 AU	0.0405	0.0396	0.0416	0.0435	0.0416	0.0386	0.0395	0.0558	0.0571	0.0601	0.0527	0.0601																								
	EF600696.1 US	0.0425	0.0415	0.0415	0.0435	0.0415	0.0405	0.0414	0.0060	0.0069	0.0096	0.0069	0.0078	0.0529																							
	HE967301.1 UY	0.0425	0.0415	0.0414	0.0435	0.0414	0.0405	0.0414	0.0060	0.0069	0.0096	0.0069	0.0096	0.0550	0.0052																						
	K02120.1 JP	0.0473	0.0483	0.0462	0.0462	0.0462	0.0473	0.0483	0.0087	0.0113	0.0123	0.0113	0.0114	0.0621	0.0104	0.0113																					
	LC080652.1 PY	0.0435	0.0425	0.0424	0.0445	0.0425	0.0415	0.0424	0.0069	0.0078	0.0087	0.0078	0.0105	0.0561	0.0061	0.0009	0.0122																				
	LC164085.1 JP	0.0404	0.0414	0.0394	0.0414	0.0394	0.0404	0.0414	0.0052	0.0078	0.0087	0.0078	0.0087	0.0549	0.0061	0.0060	0.0087	0.0069																			
	MH170028.1 VN	0.0454	0.0443	0.0443	0.0464	0.0443	0.0434	0.0443	0.0034	0.0096	0.0052	0.0096	0.0052	0.0580	0.0078	0.0078	0.0105	0.0087	0.0069																		
G2	AF257515.1 AR	0.0485	0.0474	0.0475	0.0495	0.0475	0.0464	0.0474	0.0404	0.0406	0.0444	0.0375	0.0443	0.0570	0.0396	0.0376	0.0463	0.0385	0.0395	0.0405																	
	FJ914764.1 AR	0.0475	0.0465	0.0465	0.0485	0.0465	0.0455	0.0464	0.0395	0.0396	0.0434	0.0366	0.0434	0.0582	0.0386	0.0366	0.0453	0.0376	0.0386	0.0396	0.0026																
	LC080654.1 PE	0.0445	0.0435	0.0435	0.0455	0.0435	0.0425	0.0435	0.0383	0.0385	0.0424	0.0355	0.0424	0.0529	0.0375	0.0356	0.0443	0.0366	0.0375	0.0384	0.0087	0.0078															
	LC080655.1 PY	0.0455	0.0445	0.0445	0.0465	0.0445	0.0435	0.0444	0.0355	0.0356	0.0394	0.0345	0.0394	0.0561	0.0346	0.0327	0.0413	0.0336	0.0346	0.0355	0.0061	0.0052	0.0061														
G4	AF033818.1 US	0.0269	0.0279	0.0279	0.0279	0.0288	0.0270	0.0279	0.0485	0.0486	0.0515	0.0443	0.0515	0.0425	0.0465	0.0485	0.0472	0.0496	0.0444	0.0494	0.0506	0.0496	0.0465	0.0475													
	KT122858.1 BE	0.0185	0.0195	0.0194	0.0213	0.0204	0.0185	0.0194	0.0394	0.0415	0.0443	0.0374	0.0443	0.0376	0.0395	0.0395	0.0442	0.0405	0.0375	0.0423	0.0435	0.0425	0.0395	0.0405	0.0222												
G6	LC080656.1 PY	0.0415	0.0425	0.0425	0.0445	0.0425	0.0415	0.0424	0.0525	0.0539	0.0568	0.0516	0.0569	0.0223	0.0497	0.0517	0.0545	0.0528	0.0496	0.0547	0.0548	0.0538	0.0507	0.0518	0.0385	0.0365											
	LC080658.1 PY	0.0457	0.0446	0.0467	0.0467	0.0467	0.0456	0.0466	0.0548	0.0562	0.0592	0.0538	0.0592	0.0358	0.0540	0.0540	0.0568	0.0551	0.0519	0.0570	0.0582	0.0572	0.0541	0.0551	0.0457	0.0407	0.0251										
	MF580992.1 CN	0.0535	0.0504	0.0546	0.0546	0.0546	0.0535	0.0544	0.0639	0.0653	0.0684	0.0619	0.0684	0.0434	0.0631	0.0632	0.0660	0.0642	0.0609	0.0662	0.0663	0.0653	0.0621	0.0642	0.0536	0.0485	0.0335	0.0095									
	MH170029.1 VN	0.0507	0.0518	0.0517	0.0517	0.0517	0.0508	0.0517	0.0643	0.0613	0.0689	0.0590	0.0678	0.0270	0.0593	0.0635	0.0641	0.0646	0.0612	0.0666	0.0601	0.0602	0.0570	0.0603	0.0435	0.0455	0.0168	0.0339	0.0414								
G9	LC080662.1 BO	0.0393	0.0403	0.0383	0.0403	0.0383	0.0394	0.0403	0.0334	0.0335	0.0373	0.0306	0.0373	0.0475	0.0326	0.0326	0.0372	0.0335	0.0306	0.0335	0.0260	0.0251	0.0222	0.0231	0.0413	0.0344	0.0444	0.0476	0.0555	0.0527							
	LC080663.1 BO	0.0404	0.0414	0.0393	0.0413	0.0393	0.0404	0.0413	0.0344	0.0345	0.0383	0.0315	0.0383	0.0486	0.0335	0.0335	0.0382	0.0345	0.0316	0.0344	0.0269	0.0260	0.0232	0.0241	0.0424	0.0354	0.0455	0.0487	0.0566	0.0537	0.0009						
	LC080667.1 BO	0.0413	0.0423	0.0403	0.0423	0.0403	0.0414	0.0423	0.0353	0.0355	0.0393	0.0325	0.0393	0.0496	0.0345	0.0345	0.0392	0.0355	0.0325	0.0354	0.0279	0.0270	0.0241	0.0250	0.0433	0.0364	0.0465	0.0497	0.0576	0.0548	0.0017	0.0026					
	LC080671.1 BO	0.0423	0.0433	0.0413	0.0433	0.0413	0.0423	0.0433	0.0363	0.0365	0.0403	0.0335	0.0403	0.0506	0.0355	0.0355	0.0402	0.0365	0.0335	0.0364	0.0288	0.0279	0.0250	0.0260	0.0443	0.0374	0.0475	0.0507	0.0587	0.0558	0.0026	0.0034	0.0009				
G10	LC154848.1 MM	0.0540	0.0551	0.0550	0.0572	0.0550	0.0541	0.0550	0.0621	0.0647	0.0667	0.0589	0.0656	0.0427	0.0602	0.0624	0.0644	0.0635	0.0601	0.0622	0.0601	0.0612	0.0603	0.0614	0.0539	0.0486	0.0449	0.0492	0.0569	0.0493	0.0547	0.0558	0.0568	0.0578			
	LC154849.1 MM	0.0548	0.0559	0.0558	0.0580	0.0558	0.0549	0.0559	0.0608	0.0633	0.0653	0.0577	0.0643	0.0446	0.0590	0.0611	0.0631	0.0622	0.0589	0.0631	0.0611	0.0623	0.0602	0.0624	0.0548	0.0495	0.0457	0.0500	0.0578	0.0501	0.0557	0.0568	0.0578	0.0589	0.0114		
	MF580994.1 CN	0.0657	0.0668	0.0667	0.0690	0.0667	0.0657	0.0667	0.0719	0.0745	0.0765	0.0687	0.0754	0.0552	0.0700	0.0722	0.0742	0.0733	0.0699	0.0742	0.0722	0.0733	0.0712	0.0735	0.0656	0.0602	0.0563	0.0607	0.0687	0.0608	0.0666	0.0677	0.0688	0.0698	0.0211	0.0095	
	MF580995.1 CN	0.0567	0.0578	0.0578	0.0600	0.0578	0.0568	0.0578	0.0628	0.0653	0.0673	0.0596	0.0662	0.0464	0.0609	0.0630	0.0650	0.0641	0.0608	0.0650	0.0631	0.0642	0.0622	0.0643	0.0567	0.0514	0.0476	0.0519	0.0597	0.0520	0.0576	0.0587	0.0598	0.0608	0.0131	0.0017	0.0113
		SA G4							SA G1	G1											G2				G4		G6				G9				G10		

Figure S6. Pairwise evolutionary distance of BLV full-length *gag* nucleotide sequences (1,182 bp) from the South African isolates and 29 isolates from other geographic regions worldwide. The number of base substitutions per site from between sequences are shown. The country of origin is indicated by 2-letter country codes (See Table S2). SA, South Africa.

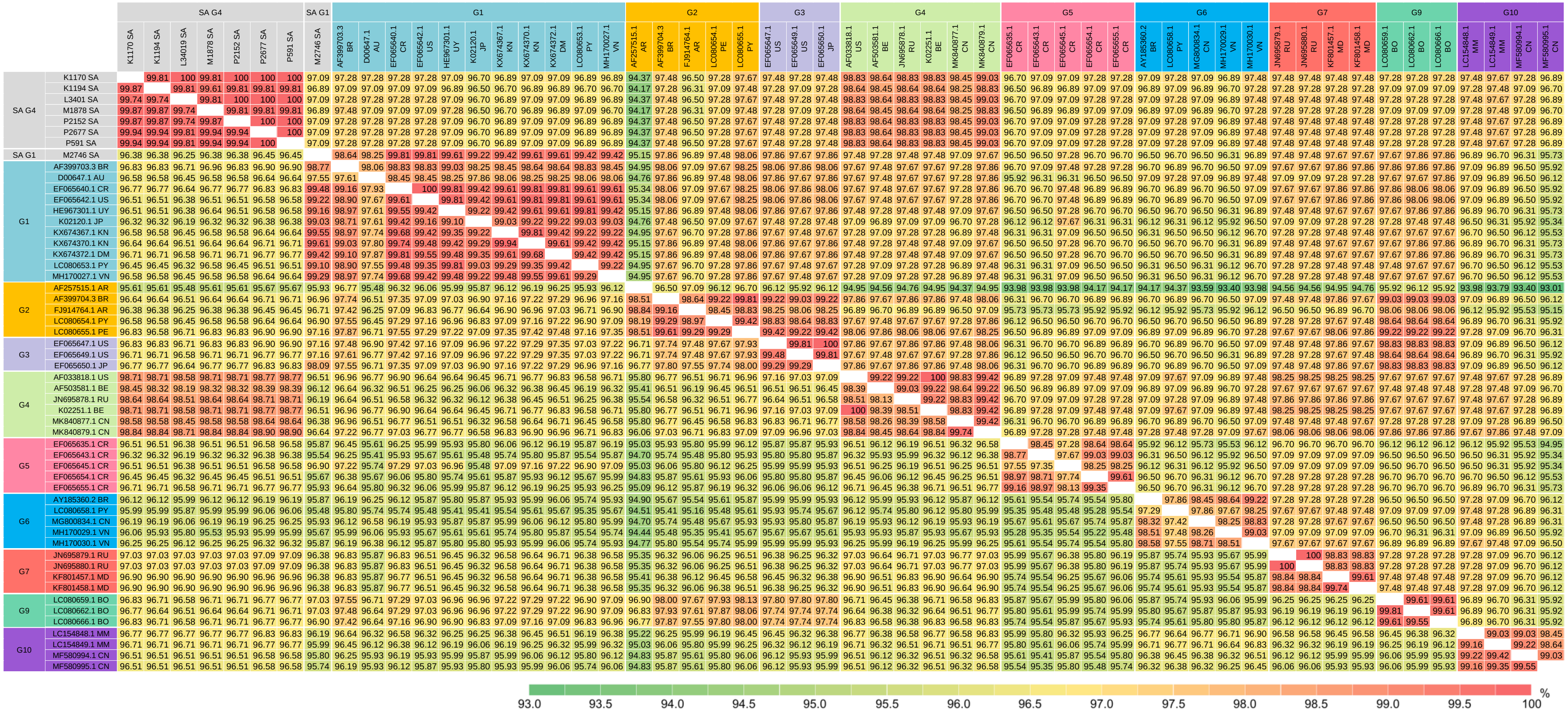

Figure S7. Pairwise percent identity of BLV full-length Env nucleotide (1,548 bp) and amino acid sequences (515 amino acids) between the South African isolates and 46 selected isolates from other geographic regions worldwide. Lowe matrix shows percent identity of nucleotide sequences and the upper matrix shows percent identity of amino acid sequences. The country of origin is indicated by 2-letter country codes (See Table S2). SA, South Africa.

		SA G4							SA G1	G1											G2					G3			G4						G5					G6					G7				G9			G10			
		K1170 SA	K1194 SA	L34019 SA	M1878 SA	P2152 SA	P2677 SA	P591 SA	M2746 SA	AF399703.3 BR	D00647.1 AU	EF065640.1 CR	EF065642.1 US	HE967301.1 UY	K02120.1 JP	KX674367.1 KN	KX674370.1 KN	KX674372.1 DM	LC080653.1 PY	MH170027.1 VN	AF257515.1 AR	AF399704.3 BR	FJ914764.1 AR	LC080654.1 PE	LC080655.1 PY	EF065647.1 US	EF065649.1 US	EF065650.1 JP	AF033818.1 US	AF503581.1 BE	JN695878.1 RU	K02251.1 BE	MK840877.1 CN	MK840879.1 CN	EF065635.1 CR	EF065643.1 CR	EF065645.1 CR	EF065654.1 CR	EF065655.1 CR	AY185360.2 BR	LC080658.1 PY	MG800834.1 CN	MH170029.1 VN	MH170030.1 VN	JN695879.1 RU	JN695880.1 RU	KF801457.1 MD	KF801458.1 MD	LC080659.1 BO	LC080662.1 BO	LC080666.1 BO	LC154848.1 MM	LC154849.1 MM	MF580994.1 CN	MF580995.1 CN
SA G4	K1170 SA		1	0	1	0	0	0	15	14	14	14	14	15	17	16	15	15	16	16	29	13	18	14	12	13	14	13	6	7	6	6	8	5	17	15	15	14	14	15	14	15	16	13	13	13	13	13	14	14	14	13	12	14	16
	K1194 SA	2		1	2	1	1	1	16	15	15	15	15	16	18	17	16	16	17	17	30	14	19	15	13	14	15	14	7	8	7	7	9	6	18	16	16	15	15	16	15	16	17	14	14	14	14	14	15	15	15	14	13	15	17
	L3401 SA	4	4		1	0	0	0	15	14	14	14	14	15	17	16	15	15	16	16	29	13	18	14	12	13	14	13	6	7	6	6	8	5	17	15	15	14	14	15	14	15	16	13	13	13	13	13	14	14	14	13	12	14	16
	M1878 SA	2	2	4		1	1	1	16	13	15	15	15	14	18	17	16	16	15	17	30	14	19	15	13	14	15	14	7	8	7	7	9	6	18	16	16	15	15	16	15	16	17	14	14	14	14	14	15	15	15	14	13	15	17
	P2152 SA	2	2	4	2		0	0	15	14	14	14	14	15	17	16	15	15	16	16	29	13	18	14	12	13	14	13	6	7	6	6	8	5	17	15	15	14	14	15	14	15	16	13	13	13	13	13	14	14	14	13	12	14	16
	P2677 SA	1	1	3	1	1		0	15	14	14	14	14	15	17	16	15	15	16	16	29	13	18	14	12	13	14	13	6	7	6	6	8	5	17	15	15	14	14	15	14	15	16	13	13	13	13	13	14	14	14	13	12	14	16
	P591 SA	1	1	3	1	1	0		15	14	14	14	14	15	17	16	15	15	16	16	29	13	18	14	12	13	14	13	6	7	6	6	8	5	17	15	15	14	14	15	14	15	16	13	13	13	13	13	14	14	14	13	12	14	16
SA G1	M2746 SA	56	56	58	56	56	55	55		7	9	1	1	2	4	3	2	2	3	3	25	11	16	13	10	11	12	11	13	14	13	13	15	12	18	18	14	17	17	18	17	18	19	16	13	13	12	12	12	11	11	16	17	19	22
G1	AF399703.3 BR	49	49	51	47	49	48	48	19		10	6	6	5	9	8	7	7	6	8	26	10	15	12	9	10	11	10	12	13	12	12	14	11	17	15	13	14	14	17	16	17	18	15	12	12	11	11	11	11	11	15	16	18	21
	D00647.1 AU	53	53	55	53	53	52	52	38	37		8	8	9	11	10	9	9	10	10	27	11	16	13	10	11	12	11	12	13	12	12	14	11	21	19	19	18	18	15	14	15	16	13	15	15	14	14	12	12	11	15	16	18	20
	EF065640.1 CR	50	50	52	50	50	49	49	8	13	32		0	1	3	2	1	1	2	2	24	10	15	12	9	10	11	10	12	13	12	12	14	11	17	17	13	16	16	17	16	17	18	15	12	12	11	11	11	10	10	15	16	18	21
	EF065642.1 US	54	54	56	54	54	53	53	12	17	36	6		1	3	2	1	1	2	2	24	10	15	12	9	10	11	10	12	13	12	12	14	11	17	17	13	16	16	17	16	17	18	15	12	12	11	11	11	10	10	15	16	18	21
	HE967301.1 UY	54	54	56	52	54	53	53	13	16	37	7	9		4	3	2	2	1	3	25	11	16	13	10	11	12	11	13	14	13	13	15	12	18	18	14	17	17	18	17	18	19	16	13	13	12	12	12	11	11	16	17	19	22
	K02120.1 JP	57	57	59	57	57	56	56	15	20	37	9	13	14		5	4	4	5	5	27	13	18	15	12	13	14	13	15	16	15	15	17	14	20	20	12	19	19	20	19	20	21	18	15	15	14	14	14	13	13	18	19	21	24
	KX674367.1 KN	53	53	55	53	53	52	52	7	16	35	5	9	10	12		1	3	4	4	26	12	17	14	11	12	13	12	14	15	14	14	16	13	19	19	15	18	18	19	18	18	20	17	14	14	13	13	12	12	12	17	18	20	23
	KX674370.1 KN	52	52	54	52	52	51	51	6	15	34	4	8	9	11	1		2	3	3	25	11	16	13	10	11	12	11	13	14	13	13	15	12	18	18	14	17	17	18	17	17	19	16	13	13	12	12	12	11	11	16	17	19	22
	KX674372.1 DM	51	51	53	51	51	50	50	9	14	33	3	7	8	10	6	5		3	3	25	11	16	13	10	11	12	11	13	14	13	13	15	12	18	18	14	17	17	18	17	18	19	16	13	13	12	12	12	11	11	16	17	19	22
	LC080653.1 PY	55	55	57	53	55	54	54	14	17	38	8	10	3	15	11	10	9		4	26	12	17	14	11	12	13	12	14	15	14	14	16	13	19	19	15	18	18	19	18	19	20	17	14	14	13	13	13	12	12	17	18	20	23
	MH170027.1 VN	53	53	55	53	53	52	52	11	16	35	5	9	8	12	8	7	6	11		26	12	17	14	11	12	13	12	14	15	14	14	16	13	19	19	15	18	18	19	18	19	20	17	14	14	13	13	12	12	12	17	18	20	23
G2	AF257515.1 AR	68	68	70	68	68	67	67	63	50	70	57	61	62	64	60	59	58	63	60		18	15	20	17	20	21	20	26	28	27	26	29	26	31	31	31	30	30	30	29	33	34	31	28	28	26	27	21	20	21	31	32	34	36
	AF399704.3 BR	52	52	54	52	52	51	51	47	35	54	41	45	46	48	44	43	42	47	44	23		7	4	1	4	5	4	11	12	11	11	13	10	19	17	17	16	16	17	16	17	18	15	13	13	11	12	5	5	5	15	16	18	20
	FJ914764.1 AR	56	56	58	56	56	55	55	51	40	58	45	49	50	52	48	47	46	51	48	18	13		8	6	9	10	9	16	17	16	16	18	15	22	22	22	21	21	20	21	22	21	20	18	18	16	17	10	10	10	20	21	23	25
	LC080654.1 PY	53	53	55	53	53	52	52	48	38	55	42	44	47	49	45	44	43	48	45	28	11	16		3	6	7	6	12	13	12	12	14	11	20	18	18	17	17	18	17	18	18	16	14	14	12	13	7	7	7	16	17	19	21
	LC080655.1 PE	49	53	51	49	49	48	48	44	33	51	38	42	43	45	41	40	39	44	41	23	6	11	11		3	4	3	10	11	10	10	12	9	18	16	16	15	15	16	15	16	17	14	12	12	10	11	4	4	4	14	15	17	19
G3	EF065647.1 US	49	49	51	49	49	48	48	44	39	48	40	44	45	47	43	42	41	46	43	51	35	39	36	32		1	0	11	12	11	11	13	10	19	17	17	16	16	17	16	17	18	15	13	13	11	12	6	6	6	15	16	18	20
	EF065649.1 US	51	51	53	51	51	50	50	44	37	50	40	44	45	47	43	42	41	46	43	51	35	39	36	32	8		1	12	13	12	12	14	11	20	18	18	17	17	18	17	18	19	16	14	14	12	13	7	7	7	16	17	19	21
	EF065650.1 JP	50	50	52	50	50	49	49	30	38	51	41	45	46	48	44	43	42	47	44	50	34	38	35	31	11	11		11	12	11	11	13	10	19	17	17	16	16	17	16	17	18	15	13	13	11	12	6	6	6	15	16	18	20
G4	AF033818.1 US	20	20	22	20	20	19	19	54	47	50	48	52	52	55	51	50	49	53	51	65	50	54	51	47	44	46	45		4	4	0	6	3	16	14	15	13	13	15	12	15	16	13	9	9	9	9	12	12	12	13	12	14	16
	AF503581.1 BE	24	26	28	26	26	25	25	60	52	57	54	58	58	61	57	56	55	59	57	71	54	59	55	54	54	54	55	25		5	4	7	4	18	16	16	15	15	16	15	16	17	14	12	12	12	12	13	13	13	14	13	15	17
	JN695878.1 RU	21	21	23	21	21	20	20	59	52	54	53	57	57	60	56	55	54	58	56	69	53	57	54	50	52	54	53	23	29		4	6	3	17	15	15	14	14	15	14	15	16	13	11	11	11	11	12	12	12	13	12	14	16
	K02251.1 BE	20	20	22	20	20	19	19	54	47	50	48	52	52	55	51	50	49	53	51	65	50	54	51	47	44	46	45	0	25	23		6	3	16	14	15	13	13	15	12	15	16	13	9	9	9	9	12	12	12	13	12	14	16
	MK840877.1 CN	22	22	24	22	22	21	21	56	47	54	50	54	54	57	53	52	51	55	53	65	50	55	53	49	49	51	50	22	27	25	22		3	19	17	17	16	16	17	16	17	18	15	13	13	13	12	14	14	14	15	14	16	18
	MK840879.1 CN	18	18	20	18	18	17	17	52	43	50	46	50	50	53	49	48	47	51	49	61	46	51	49	45	45	47	46	18	24	21	18	4		16	14	14	13	13	14	13	14	15	12	10	10	10	10	11	11	11	12	11	13	15
G5	EF065635.1 CR	54	54	56	54	54	53	53	64	55	68	58	62	63	65	61	60	59	64	59	77	63	65	62	60	64	64	63	54	60	59	54	57	53		8	14	7	7	21	20	22	23	20	17	17	17	17	20	20	20	20	21	23	26
	EF065643.1 CR	57	57	59	57	57	56	56	69	58	71	63	67	68	70	66	65	64	69	64	82	66	70	65	63	65	65	64	57	63	62	57	60	56	19		12	5	5	19	18	20	21	18	15	15	15	15	18	18	18	18	19	21	24
	EF065645.1 CR	54	54	56	54	54	53	53	48	43	66	42	46	47	70	45	44	43	48	45	77	61	65	60	58	62	62	63	54	58	59	54	58	54	38	41		9	9	20	19	20	21	18	15	15	15	15	18	17	18	18	19	21	24
	EF065654.1 CR	55	55	57	55	55	54	54	67	56	67	61	65	66	68	64	63	60	67	62	80	64	68	63	61	65	65	64	55	61	60	55	58	54	16	20	35		2	18	17	19	20	17	14	14	14	14	17	17	17	17	18	20	23
	EF065655.1 CR	51	51	53	51	51	50	50	63	52	65	57	61	62	64	60	59	58	63	58	76	60	64	59	57	61	61	60	51	55	56	51	54	50	13	16	29	10		18	17	19	20	17	14	14	14	14	17	17	17	16	17	19	22
G6	AY185360.2 BR	60	60	62	60	60	59	59	64	59	58	60	64	65	64	63	62	61	66	63	79	67	69	68	64	62	62	63	60	62	63	60	64	60	68	69	66	69	65		11	8	7	4	14	14	14	14	18	18	18	14	15	17	20
	LC080658.1 PY	62	62	64	62	62	61	61	70	65	66	66	70	71	71	69	68	67	72	67	85	71	75	70	68	63	66	67	60	66	65	60	65	62	72	70	70	73	69	42		11	12	9	12	12	13	13	15	15	15	13	14	16	19
	MG800834.1 CN	59	59	61	59	59	58	58	63	60	53	59	63	64	64	62	61	60	65	62	82	66	70	67	63	63	65	64	59	63	60	59	63	59	67	68	67	66	64	26	40		9	6	14	14	12	12	18	18	18	13	14	16	19
	MH170029.1 VN	61	63	65	69	63	62	62	67	62	61	63	67	68	68	66	65	64	69	66	86	70	72	71	67	67	67	68	63	63	64	63	67	63	73	72	69	74	70	23	39	27		5	15	15	15	15	17	19	19	15	16	18	21
	MH170030.1 VN	58	58	60	58	58	57	57	64	59	56	60	64	65	65	63	62	61	66	63	81	65	69	66	62	62	62	63	58	62	59	58	62	58	68	69	66	69	65	22	38	20	23		12	12	12	12	16	16	16	12	13	15	18
G7	JN695879.1 RU	46	46	46	46	46	45	45	56	49	64	49	54	55	57	53	52	51	56	53	72	57	61	58	54	56	58	57	46	52	51	46	50	46	62	67	56	65	59	64	66	63	67	62		0	6	6	14	14	14	14	15	17	20
	JN695880.1 RU	46	46	46	46	46	45	45	56	49	64	49	54	55	57	53	52	51	56	53	72	57	61	58	54	56	58	57	46	52	51	46	50	46	62	67	56	65	59	64	66	63	67	62	0		6	6	14	14	14	14	15	17	20
	KF801457.1 MD	48	48	48	48	48	47	47	56	49	64	50	54	55	57	53	52	51	56	53	71	56	60	55	53	55	57	56	48	54	49	48	52	48	66	69	58	67	61	66	68	63	69	64	18	18		2	13	13	13	14	15	17	20
	KF801458.1 MD	48	48	48	48	48	47	47	56	49	64	50	54	55	57	53	52	51	56	53	72	57	61	56	54	56	58	57	48	54	49	48	52	48	66	69	58	67	61	66	68	63	69	64	18	18	4		13	13	13	14	15	17	20
G9	LC080659.1 BO	49	51	53	51	51	50	50	46	38	51	42	46	47	47	43	42	43	48	45	48	31	36	32	29	34	34	34	51	55	56	51	53	49	64	67	62	65	61	64	66	63	61	62	58	58	58	58		2	2	16	17	19	21
	LC080662.1 BO	50	52	54	52	52	51	51	46	39	52	42	46	47	47	43	42	43	48	45	49	32	37	33	30	35	35	35	52	56	57	52	54	50	65	68	62	66	62	65	67	64	64	63	59	59	59	59	3		2	16	17	19	21
	LC080666.1 BO	49	51	53	51	51	50	50	48	40	52	44	48	48	49	45	44	45	49	47	50	33	38	34	31	35	35	35	49	53	56	49	53	49	66	69	64	67	63	66	68	65	65	64	60	60	60	60	6	7		16	17	19	21
G10	LC154848.1 MM	50	50	50	50	50	49	49	59	52	57	53	57	58	58	56	55	54	59	56	74	58	62	59	55	55	57	56	50	56	53	50	53	49	62	65	57	63	58	50	52	50	51	48	53	53	55	53	55	56	57		5	5	8
	LC154849.1 MM	51	51	51	51	51	50	50	62	55	60	56	60	59	61	59	58	57	62	57	77	61	65	62	58	57	60	59	51	57	54	51	54	50	65	68	61	66	62	51	50	51	52	49	57	57	59	59	58	59	60	13		4	7
	MF580994.1 CN	54	54	54	54	54	53	53	65	58	63	59	63	62	64	62	61	60	65	60	80	64	68	65	61	60	63	62	54	60	57	54	57	53	68	71	64	69	65	56	55	56	57	54	60	60	62	62	61	62	63	12	9		5
	MF580995.1 CN	54	54	54	54	54	53	53	66	59	63	60	64	63	65	63	62	61	66	61	80	64	68	65	61	60	63	62	54	60	57	54	57	53	69	72	65	70	66	57	56	57	58	55	61	61	63	63	61	62	63	13	10	7	

0 5 10 15 20 25 30 35 40 45 50 55 60 65 70 75 80 85

Figure S8. Pairwise comparison of BLV full-length Env nucleotide (1,548 bp) and amino acid (515 amino acids) differences between the South African isolates and 46 selected isolates from other geographic regions worldwide. Lower matrix shows the number of nucleotide differences and the upper matrix shows the number of amino acid differences. The country of origin is indicated by 2-letter country codes (See Table S2). SA, South Africa.

		SA G4							SA G1	G1											G4								
		K1170 SA	K1194 SA	L3401 SA	M1878 SA	P2152 SA	P2677 SA	P591 SA	M2746 SA	AP018007.1 JP	EU266060.1 IR	FJ808578.1 AR	HE967302.1 UY	KJ707242.1 KR	KX674367.1 KN	KX674372.1 DM	LC060800.1 MN	LC075543.1 PE	LC075568.1 BO	LC080653.1 PY	AF067081.1 PL	AY515278.1 CL	EU262575.2 PL	FJ808596.1 AR	HQ902258.1 BY	JQ686101.1 RU	LC060795.1 MN	M35238.1 FR	U87872.1 DE
SA G4	K1170 SA		100	100	99.32	100	100	100	97.30	95.95	94.59	96.62	97.30	96.62	97.97	97.97	95.27	97.97	97.97	96.62	98.65	97.30	97.97	97.97	97.97	96.62	99.32	99.32	97.97
	K1194 SA	100		100	99.32	100	100	100	97.30	95.95	94.59	96.62	97.30	96.62	97.97	97.97	95.27	97.97	97.97	96.62	98.65	97.30	97.97	97.97	97.97	96.62	99.32	99.32	97.97
	L3401 SA	99.77	99.77		99.32	100	100	100	97.30	95.95	94.59	96.62	97.30	96.62	97.97	97.97	95.27	97.97	97.97	96.62	98.65	97.30	97.97	97.97	97.97	96.62	99.32	99.32	97.97
	M1878 SA	99.77	99.77	99.55		99.32	99.32	99.32	96.62	95.27	93.92	97.30	97.97	95.95	97.30	97.30	94.59	97.30	97.30	97.30	97.97	96.62	97.30	97.30	97.30	95.95	98.65	98.65	97.30
	P2152 SA	99.77	99.77	99.55	99.55		100	100	97.30	95.95	94.59	96.62	97.30	96.62	97.97	97.97	95.27	97.97	97.97	96.62	98.65	97.30	97.97	97.97	97.97	96.62	99.32	99.32	97.97
	P2677 SA	100	100	99.77	99.77	99.77		100	97.30	95.95	94.59	96.62	97.30	96.62	97.97	97.97	95.27	97.97	97.97	96.62	98.65	97.30	97.97	97.97	97.97	96.62	99.32	99.32	97.97
	P591 SA	100	100	99.77	99.77	99.77	100		97.30	95.95	94.59	96.62	97.30	96.62	97.97	97.97	95.27	97.97	97.97	96.62	98.65	97.30	97.97	97.97	97.97	96.62	99.32	99.32	97.97
SA G1	M2746 SA	97.07	97.07	96.85	96.85	96.85	97.07	97.07		97.30	95.95	97.97	97.30	97.97	99.32	99.32	96.62	99.32	99.32	97.97	98.65	97.30	97.97	97.97	97.97	96.62	97.97	97.97	97.97
G1	AP018007.1 JP	96.40	96.40	96.17	96.17	96.17	96.40	96.40	98.42		94.59	96.62	95.95	96.62	97.97	97.97	95.27	97.97	97.97	96.62	97.30	96.62	96.62	97.30	96.62	95.27	96.62	96.62	96.62
	EU266060.1 IR	95.50	95.50	95.27	95.27	95.27	95.50	95.50	97.30	96.62		96.62	95.95	95.27	96.62	96.62	93.92	96.62	96.62	96.62	95.95	94.59	95.27	95.27	95.27	93.92	95.27	95.27	95.27
	FJ808578.1 AR	97.07	97.07	96.85	97.30	96.85	97.07	97.07	98.87	98.20	97.52		99.32	97.30	98.65	98.65	95.95	98.65	98.65	100	97.97	96.62	97.30	97.30	97.30	95.95	97.30	97.30	97.30
	HE967302.1 UY	97.30	97.30	97.07	97.52	97.07	97.30	97.30	98.65	97.97	97.30	99.77		96.62	97.97	97.97	95.27	97.97	97.97	99.32	98.65	97.30	97.97	97.97	97.97	96.62	97.97	97.97	97.97
	KJ707242.1 KR	96.40	96.40	96.17	96.17	96.17	96.40	96.40	98.20	97.52	96.40	97.97	97.75		98.65	98.65	95.95	98.65	98.65	97.30	97.97	96.62	97.30	97.30	97.30	95.95	97.30	97.30	97.30
	KX674367.1 KN	97.52	97.52	97.30	97.30	97.30	97.52	97.52	99.55	98.87	97.75	99.32	99.10	98.65		100	97.30	100	100	98.65	99.32	97.97	98.65	98.65	98.65	97.30	98.65	98.65	98.65
	KX674372.1 DM	97.30	97.30	97.07	97.07	97.07	97.30	97.30	99.32	98.65	97.52	99.10	98.87	98.42	99.77		97.30	100	100	98.65	99.32	97.97	98.65	98.65	98.65	97.30	98.65	98.65	98.65
	LC060800.1 MN	96.40	96.40	96.17	96.17	96.17	96.40	96.40	98.42	97.75	97.07	98.20	97.97	97.52	98.87	98.65		97.30	97.30	95.95	96.62	95.27	95.95	95.95	95.95	95.27	95.95	95.95	95.95
	LC075543.1 PE	97.30	97.30	97.07	97.07	97.07	97.30	97.30	99.32	98.65	97.52	99.10	98.87	98.42	99.77	99.55	98.65		100	98.65	99.32	97.97	98.65	98.65	98.65	97.30	98.65	98.65	98.65
	LC075568.1 BO	97.07	97.07	96.85	96.85	96.85	97.07	97.07	99.10	98.42	97.30	98.87	98.65	98.20	99.55	99.32	98.42	99.32		98.65	99.32	97.97	98.65	98.65	98.65	97.30	98.65	98.65	98.65
	LC080653.1 PY	96.85	96.85	96.62	97.07	96.62	96.85	96.85	98.65	97.97	97.30	99.77	99.55	97.75	99.10	98.87	97.97	98.87	98.65		97.97	96.62	97.30	97.30	97.30	95.95	97.30	97.30	97.30
G4	AF067081.1 PL	99.10	99.10	98.87	98.87	98.87	99.10	99.10	97.07	96.40	95.50	97.07	97.30	96.40	97.52	97.75	96.40	97.30	97.07	96.85		98.65	99.32	99.32	99.32	97.97	99.32	99.32	99.32
	AY515278.1 CL	98.20	98.20	97.97	97.97	97.97	98.20	98.20	97.07	96.62	95.50	97.07	97.30	96.17	97.52	97.30	96.40	97.75	97.07	96.85	98.20		97.97	97.97	97.97	96.62	97.97	97.97	97.97
	EU262575.2 PL	98.87	98.87	98.65	98.65	99.10	98.87	98.87	96.85	96.17	95.27	96.85	97.07	96.17	97.30	97.07	96.17	97.07	96.85	96.62	98.87	97.97		98.65	98.65	97.30	98.65	98.65	98.65
	FJ808596.1 AR	98.65	98.65	98.42	98.42	98.42	98.65	98.65	96.62	95.95	95.05	96.62	96.85	95.95	97.07	96.85	95.95	96.85	96.62	96.40	98.65	97.75	98.87		98.65	97.30	98.65	98.65	98.65
	HQ902258.1 BY	98.65	98.65	98.42	98.42	98.42	98.65	98.65	97.07	96.40	95.50	97.07	97.30	96.17	97.52	97.30	96.40	97.30	97.07	96.85	98.65	98.20	98.42	98.20		97.30	98.65	98.65	98.65
	JQ686101.1 RU	98.42	98.42	98.20	98.20	98.20	98.42	98.42	96.40	95.72	94.82	96.40	96.62	95.72	96.85	96.62	95.72	96.62	96.40	96.17	98.42	97.52	98.20	97.97	97.97		97.30	97.30	97.30
	LC060795.1 MN	99.10	99.10	98.87	98.87	98.87	99.10	99.10	97.07	96.40	95.50	97.07	97.30	96.17	97.52	97.30	96.40	97.30	97.07	96.85	98.65	98.20	98.42	98.20	98.65	97.97		100	98.65
	M35238.1 FR	99.55	99.55	99.32	99.32	99.32	99.55	99.55	97.07	96.40	95.50	97.07	97.30	96.40	97.52	97.30	96.40	97.30	97.52	96.85	99.10	98.20	98.87	98.65	98.65	98.42	99.10		98.65
	U87872.1 DE	97.97	97.97	97.75	97.75	97.75	97.97	97.97	96.40	95.72	94.82	96.40	96.62	95.95	96.85	96.62	95.72	96.62	96.40	96.17	97.97	97.52	97.75	97.52	97.52	97.30	97.52	97.97	

94.0 94.5 95.0 95.5 96.0 96.5 97.0 97.5 98.0 98.5 99.0 99.5 100 %

Figure S9. Pairwise percent identity of the BLV partial Env nucleotide (444 bp) and amino acid (148 amino acids) sequences between G1 and G4 isolates from South Africa and other geographic regions worldwide. Lower matrix shows percent identity of nucleotide sequences and the upper matrix shows percent identity of amino acid sequences. The country of origin is indicated by 2-letter country codes (See Table S2). SA, South Africa.

		SA G4							SA G1	G1											G4								
		K1170 SA	K1194 SA	L3401 SA	M1878 SA	P2152 SA	P2677 SA	P591 SA	M2746 SA	AP018007.1 JP	EU266060.1 IR	FJ808578.1 AR	HE967302.1 UY	KJ707242.1 KR	KX674367.1 KN	KX674372.1 DM	LC060800.1 MN	LC075543.1 PE	LC075568.1 BO	LC080653.1 PY	AF067081.1 PL	AY515278.1 CL	EU262575.2 PL	FJ808596.1 AR	HQ902258.1 BY	JQ686101.1 RU	LC060795.1 MN	M35238.1 FR	U87872.1 DE
SA G4	K1170 SA		0	0	1	0	0	0	4	6	8	5	4	5	3	3	7	3	3	5	2	4	3	3	3	5	1	1	3
	K1194 SA	0		0	1	0	0	0	4	6	8	5	4	5	3	3	7	3	3	5	2	4	3	3	3	5	1	1	3
	L3401 SA	1	1		1	0	0	0	4	6	8	5	4	5	3	3	7	3	3	5	2	4	3	3	3	5	1	1	3
	M1878 SA	1	1	2		1	1	1	5	7	9	4	3	6	4	4	8	4	4	4	3	5	4	4	4	6	2	2	4
	P2152 SA	1	1	2	2		0	0	4	6	8	5	4	5	3	3	7	3	3	5	2	4	3	3	3	5	1	1	3
	P2677 SA	0	0	1	1	1		0	4	6	8	5	4	5	3	3	7	3	3	5	2	4	3	3	3	5	1	1	3
	P591 SA	0	0	1	1	1	0		4	6	8	5	4	5	3	3	7	3	3	5	2	4	3	3	3	5	1	1	3
SA G1	M2746 SA	13	13	14	14	14	13	13		4	6	3	4	3	1	1	5	1	1	3	2	4	3	3	3	5	3	3	3
G1	AP018007.1 JP	16	16	17	17	17	16	16	7		8	5	6	5	3	3	7	3	3	5	4	5	5	4	5	7	5	5	5
	EU266060.1 IR	20	20	21	21	21	20	20	12	15		5	6	7	5	5	9	5	5	5	6	8	7	7	7	9	7	7	7
	FJ808578.1 AR	13	13	14	12	14	13	13	5	8	11		1	4	2	2	6	2	2	0	3	5	4	4	4	6	4	4	4
	HE967302.1 UY	12	12	13	11	13	12	12	6	9	12	1		5	3	3	7	3	3	1	2	4	3	3	3	5	3	3	3
	KJ707242.1 KR	16	16	17	17	17	16	16	8	11	16	9	10		2	2	6	2	2	4	3	5	4	4	4	6	4	4	4
	KX674367.1 KN	11	11	12	12	12	11	11	2	5	10	3	4	6		0	4	0	0	2	1	3	2	2	2	4	2	2	2
	KX674372.1 DM	12	12	13	13	13	12	12	3	6	11	4	5	7	1		4	0	0	2	1	3	2	2	2	4	2	2	2
	LC060800.1 MN	16	16	17	17	17	16	16	7	10	13	8	9	11	5	6		4	4	6	5	7	6	6	6	7	6	6	6
	LC075543.1 PE	12	12	13	13	13	12	12	3	6	11	4	5	7	1	2	6		0	2	1	3	2	2	2	4	2	2	2
	LC075568.1 BO	13	13	14	14	14	13	13	4	7	12	5	6	8	2	3	7	3		2	1	3	2	2	2	4	2	2	2
	LC080653.1 PY	14	14	15	13	15	14	14	6	9	12	1	2	10	4	5	9	5	6		3	5	4	4	4	6	4	4	4
G4	AF067081.1 PL	4	4	5	5	5	4	4	13	16	20	13	12	16	11	10	16	12	13	14		2	1	1	1	3	1	1	1
	AY515278.1 CL	8	8	9	9	9	8	8	13	15	20	13	12	17	11	12	16	10	13	14	8		3	3	3	5	3	3	3
	EU262575.2 PL	5	5	6	6	4	5	5	14	17	21	14	13	17	12	13	17	13	14	15	5	9		2	2	4	2	2	2
	FJ808596.1 AR	6	6	7	7	7	6	6	15	18	22	15	14	18	13	14	18	14	15	16	6	10	5		2	4	2	2	2
	HQ902258.1 BY	6	6	7	7	7	6	6	13	16	20	13	12	17	11	12	16	12	13	14	6	8	7	8		4	2	2	2
	JQ686101.1 RU	7	7	8	8	8	7	7	16	19	23	16	15	19	14	15	19	15	16	17	7	11	8	9	9		4	4	4
	LC060795.1 MN	4	4	5	5	5	4	4	13	16	20	13	12	17	11	12	16	12	13	14	6	8	7	8	6	9		0	2
	M35238.1 FR	2	2	3	3	3	2	2	13	16	20	13	12	16	11	12	16	12	11	14	4	8	5	6	6	7	4		2
	U87872.1 DE	9	9	10	10	10	9	9	16	19	23	16	15	18	14	15	19	15	16	17	9	11	10	11	11	12	11	9	

0 5 10 15 20 25

Figure S10. Pairwise comparison of BLV partial Env nucleotide (444 bp) and amino acid (148 amino acids) differences between G1 and G4 isolates from South Africa and other geographic regions worldwide. Lower matrix shows the number of nucleotide differences and the upper matrix shows the number of amino acid differences. The country of origin is indicated by 2-letter country codes (See Table S2). SA, South Africa.

Table S2. Pairwise comparison of BLV partial Env nucleotide and amino acid sequences between G1 South African and Zambian sequences.

	Comparison with South African G1 (M2746)	Percent identity	Differences
Zambian G1	LC440653.1	98.87% (98.65%)	5 (2)
	LC440654.1	98.87% (98.65%)	5 (2)
	LC440655.1	98.87% (98.65%)	5 (2)
	LC440656.1	98.87% (98.65%)	5 (2)
	LC440657.1	98.87% (98.65%)	5 (2)
	LC440658.1	98.87% (98.65%)	5 (2)
	LC440659.1	98.87% (98.65%)	5 (2)
	LC440660.1	98.87% (98.65%)	5 (2)
	LC440661.1	98.20% (96.62%)	8 (5)
	LC440662.1	98.87% (98.65%)	5 (2)
	LC440663.1	98.42% (97.30%)	7 (4)
	LC440664.1	98.87% (98.65%)	5 (2)
	LC440665.1	98.87% (98.65%)	5 (2)
	LC440666.1	98.87% (98.65%)	5 (2)

Percent identity of amino acid sequences and the number of amino acid differences are shown in brackets.

Table S3. Pairwise comparison of BLV partial Env nucleotide and amino acid sequences between G4 South African and Zambian sequences.

	Comparison with Zambian G4 (LC193462.1)	Percent identity	Differences
South African G4	K1170	99.77% (100%)	1 (0)
	K1194	99.77% (100%)	1 (0)
	L3401	99.55% (100%)	2 (0)
	M1878	99.55% (99.32%)	2 (1)
	P2152	99.55% (100%)	2 (0)
	P2677	99.77% (100%)	1 (0)
	P591	99.77% (100%)	1 (0)

Percent identity of amino acid sequences and the number of amino acid differences are shown in brackets.

		94	95	96	97	98	99	100	101	102	103	104	105	106	107	108	109	110	111	112	113	114	115	116	117	118	119	120	121	122	123	124	125	126	127	128	129	130
Epitopes and motifs			ND1																																			
		CD4+ T cell epitope																										G										
			F									Zinc binding site																										
Amino acid position		94	95	96	97	98	99	100	101	102	103	104	105	106	107	108	109	110	111	112	113	114	115	116	117	118	119	120	121	122	123	124	125	126	127	128	129	130
SA G1	M2746 SA	P	Q	G	R	R	R	F	G	A	R	A	M	V	T	Y	D	C	E	P	R	C	P	Y	V	G	A	D	R	F	D	C	P	H	W	D	N	A
G1	LC440653.1 ZM	•	•	•	•	•	•	•	•	•	•	•	•	•	•	•	•	•	•	•	•	•	•	•	•	•	•	•	•	•	•	•	•	•	•	•	•	•
G1	LC440654.1 ZM	•	•	•	•	•	•	•	•	•	•	•	•	•	•	•	•	•	•	•	•	•	•	•	•	•	•	•	•	•	•	•	•	•	•	•	•	•
G1	LC440655.1 ZM	•	•	•	•	•	•	•	•	•	•	•	•	•	•	•	•	•	•	•	•	•	•	•	•	•	•	•	•	•	•	•	•	•	•	•	•	•
G1	LC440656.1 ZM	•	•	•	•	•	•	•	•	•	•	•	•	•	•	•	•	•	•	•	•	•	•	•	•	•	•	•	•	•	•	•	•	•	•	•	•	•
G1	LC440657.1 ZM	•	•	•	•	•	•	•	•	•	•	•	•	•	•	•	•	•	•	•	•	•	•	•	•	•	•	•	•	•	•	•	•	•	•	•	•	•
G1	LC440658.1 ZM	•	•	•	•	•	•	•	•	•	•	•	•	•	•	•	•	•	•	•	•	•	•	•	•	•	•	•	•	•	•	•	•	•	•	•	•	•
G1	LC440659.1 ZM	•	•	•	•	•	•	•	•	•	•	•	•	•	•	•	•	•	•	•	•	•	•	•	•	•	•	•	•	•	•	•	•	•	•	•	•	•
G1	LC440660.1 ZM	•	•	•	•	•	•	•	•	•	•	•	•	•	•	•	•	•	•	•	•	•	•	•	•	•	•	•	•	•	•	•	•	•	•	•	•	•
G1	LC440661.1 ZM	•	•	•	•	•	•	•	•	•	•	•	•	•	•	•	•	•	•	•	•	•	•	•	•	•	•	•	•	•	•	•	•	G	•	•	•	•
G1	LC440662.1 ZM	•	•	•	•	•	•	•	•	•	•	•	•	•	•	•	•	•	•	•	•	•	•	•	•	•	•	•	•	•	•	•	•	•	•	•	•	•
G1	LC440663.1 ZM	•	•	•	•	•	•	•	•	•	•	•	•	•	•	•	•	•	•	•	•	•	•	•	•	•	•	•	•	•	N	•	•	•	•	•	•	•
G1	LC440664.1 ZM	•	•	•	•	•	•	•	•	•	•	•	•	•	•	•	•	•	•	•	•	•	•	•	•	•	•	•	•	•	•	•	•	•	•	•	•	•
G1	LC440665.1 ZM	•	•	•	•	•	•	•	•	•	•	•	•	•	•	•	•	•	•	•	•	•	•	•	•	•	•	•	•	•	•	•	•	•	•	•	•	•
G1	LC440666.1 ZM	•	•	•	•	•	•	•	•	•	•	•	•	•	•	•	•	•	•	•	•	•	•	•	•	•	•	•	•	•	•	•	•	•	•	•	•	•
G4	LC193462.1 ZM	P	Q	G	R	R	R	F	G	A	R	A	M	V	T	Y	D	C	E	P	R	C	P	Y	V	G	A	D	H	F	D	C	P	H	W	D	N	A
SA G4	K1170 SA	•	•	•	•	•	•	•	•	•	•	•	•	•	•	•	•	•	•	•	•	•	•	•	•	•	•	•	•	•	•	•	•	•	•	•	•	•
SA G4	K1194 SA	•	•	•	•	•	•	•	•	•	•	•	•	•	•	•	•	•	•	•	•	•	•	•	•	•	•	•	•	•	•	•	•	•	•	•	•	•
SA G4	L3401 SA	•	•	•	•	•	•	•	•	•	•	•	•	•	•	•	•	•	•	•	•	•	•	•	•	•	•	•	•	•	•	•	•	•	•	•	•	•
SA G4	M1878 SA	•	•	•	•	•	•	•	•	•	•	•	•	•	•	•	•	•	•	•	•	•	•	•	•	•	•	•	•	•	•	•	•	•	•	•	•	•
SA G4	P2152 SA	•	•	•	•	•	•	•	•	•	•	•	•	•	•	•	•	•	•	•	•	•	•	•	•	•	•	•	•	•	•	•	•	•	•	•	•	•
SA G4	P2677 SA	•	•	•	•	•	•	•	•	•	•	•	•	•	•	•	•	•	•	•	•	•	•	•	•	•	•	•	•	•	•	•	•	•	•	•	•	•
SA G4	P591 SA	•	•	•	•	•	•	•	•	•	•	•	•	•	•	•	•	•	•	•	•	•	•	•	•	•	•	•	•	•	•	•	•	•	•	•	•	•

		131	132	133	134	135	136	137	138	139	140	141	142	143	144	145	146	147	148	149	150	151	152	153	154	155	156	157	158	159	160	161	162	163	164	165	166	167
Epitopes and motifs		ND2																							CD8+ T cell epitope													
Amino acid position		131	132	133	134	135	136	137	138	139	140	141	142	143	144	145	146	147	148	149	150	151	152	153	154	155	156	157	158	159	160	161	162	163	164	165	166	167
SA G1	M2746 SA	S	Q	A	D	Q	G	S	F	Y	V	N	H	Q	I	L	F	L	H	L	K	Q	C	Q	G	I	F	T	L	T	W	E	I	W	G	Y	D	P
G1	LC440653.1 ZM	•	•	•	•	•	•	•	•	•	•	•	•	•	•	•	•	•	•	•	•	•	•	H	•	•	•	•	•	•	•	•	•	•	•	•	•	•
G1	LC440654.1 ZM	•	•	•	•	•	•	•	•	•	•	•	•	•	•	•	•	•	•	•	•	•	•	H	•	•	•	•	•	•	•	•	•	•	•	•	•	•
G1	LC440655.1 ZM	•	•	•	•	•	•	•	•	•	•	•	•	•	•	•	•	•	•	•	•	•	•	H	•	•	•	•	•	•	•	•	•	•	•	•	•	•
G1	LC440656.1 ZM	•	•	•	•	•	•	•	•	•	•	•	•	•	•	•	•	•	•	•	•	•	•	H	•	•	•	•	•	•	•	•	•	•	•	•	•	•
G1	LC440657.1 ZM	•	•	•	•	•	•	•	•	•	•	•	•	•	•	•	•	•	•	•	•	•	•	H	•	•	•	•	•	•	•	•	•	•	•	•	•	•
G1	LC440658.1 ZM	•	•	•	•	•	•	•	•	•	•	•	•	•	•	•	•	•	•	•	•	•	•	H	•	•	•	•	•	•	•	•	•	•	•	•	•	•
G1	LC440659.1 ZM	•	•	•	•	•	•	•	•	•	•	•	•	•	•	•	•	•	•	•	•	•	•	H	•	•	•	•	•	•	•	•	•	•	•	•	•	•
G1	LC440660.1 ZM	•	•	•	•	•	•	•	•	•	•	•	•	•	•	•	•	•	•	•	•	•	•	H	•	•	•	•	•	•	•	•	•	•	•	•	•	•
G1	LC440661.1 ZM	•	•	•	•	•	•	•	•	•	•	•	•	•	•	•	•	•	•	•	•	•	•	H	•	•	•	•	•	•	•	•	T	•	•	•	•	•
G1	LC440662.1 ZM	•	•	•	•	•	•	•	•	•	•	•	•	•	•	•	•	•	•	•	•	•	•	H	•	•	•	•	•	•	•	•	•	•	•	•	•	•
G1	LC440663.1 ZM	•	•	•	•	•	•	•	•	•	•	•	•	•	•	•	•	•	•	•	•	•	•	H	•	•	•	•	•	•	•	•	•	•	•	•	•	•
G1	LC440664.1 ZM	•	•	•	•	•	•	•	•	•	•	•	•	•	•	•	•	•	•	•	•	•	•	H	•	•	•	•	•	•	•	•	•	•	•	•	•	•
G1	LC440665.1 ZM	•	•	•	•	•	•	•	•	•	•	•	•	•	•	•	•	•	•	•	•	•	•	H	•	•	•	•	•	•	•	•	•	•	•	•	•	•
G1	LC440666.1 ZM	•	•	•	•	•	•	•	•	•	•	•	•	•	•	•	•	•	•	•	•	•	•	H	•	•	•	•	•	•	•	•	•	•	•	•	•	•
G4	LC193462.1 ZM	S	R	A	D	Q	G	S	F	Y	V	N	H	Q	T	L	F	L	H	L	K	Q	C	H	G	I	F	T	L	T	W	E	I	W	G	Y	D	P
SA G4	K1170 SA	•	•	•	•	•	•	•	•	•	•	•	•	•	•	•	•	•	•	•	•	•	•	•	•	•	•	•	•	•	•	•	•	•	•	•	•	•
SA G4	K1194 SA	•	•	•	•	•	•	•	•	•	•	•	•	•	•	•	•	•	•	•	•	•	•	•	•	•	•	•	•	•	•	•	•	•	•	•	•	•
SA G4	L3401 SA	•	•	•	•	•	•	•	•	•	•	•	•	•	•	•	•	•	•	•	•	•	•	•	•	•	•	•	•	•	•	•	•	•	•	•	•	•
SA G4	M1878 SA	•	•	•	N	•	•	•	•	•	•	•	•	•	•	•	•	•	•	•	•	•	•	•	•	•	•	•	•	•	•	•	•	•	•	•	•	•
SA G4	P2152 SA	•	•	•	•	•	•	•	•	•	•	•	•	•	•	•	•	•	•	•	•	•	•	•	•	•	•	•	•	•	•	•	•	•	•	•	•	•
SA G4	P2677 SA	•	•	•	•	•	•	•	•	•	•	•	•	•	•	•	•	•	•	•	•	•	•	•	•	•	•	•	•	•	•	•	•	•	•	•	•	•
SA G4	P591 SA	•	•	•	•	•	•	•	•	•	•	•	•	•	•	•	•	•	•	•	•	•	•	•	•	•	•	•	•	•	•	•	•	•	•	•	•	•

Figure S11. Comparison of 148 amino acid region in the Env gp51 protein between the G1 and G4 South African and Zambian isolates. G1 South African isolate M2746 was used as a consensus sequence for the comparison with other G1 isolates and G4 Zambian isolate LC193462.1 for the comparison with other G4 isolates. A dot indicates homology with the consensus sequence. SA, South Africa; ZM, Zambia. Colour bars above the sequence alignment show positions of epitopes and motifs including neutralising domains (ND1, ND2 and ND3), conformational epitopes (F and G), a linear epitope (E), CD8^{+} T cell epitope, CD4^{+} T cell epitope, a position of disulphide bond formation and a zinc binding site.

Epitopes and motifs									E																													
		CD8+ T cell epitope																																				
Amino acid position		168	169	170	171	172	173	174	175	176	177	178	179	180	181	182	183	184	185	186	187	188	189	190	191	192	193	194	195	196	197	198	199	200	201	202	203	204
SA G1	M2746 SA	L	I	T	F	S	L	H	K	I	P	D	P	P	Q	P	D	F	P	Q	L	N	S	D	W	V	P	S	V	R	S	W	A	L	L	L	N	Q
G1	LC440653.1 ZM	•	•	•	•	•	•	•	•	•	•	•	•	•	•	•	•	•	•	•	•	•	•	•	•	•	•	•	•	•	•	•	•	•	•	•	•	•
	LC440654.1 ZM	•	•	•	•	•	•	•	•	•	•	•	•	•	•	•	•	•	•	•	•	•	•	•	•	•	•	•	•	•	•	•	•	•	•	•	•	•
	LC440655.1 ZM	•	•	•	•	•	•	•	•	•	•	•	•	•	•	•	•	•	•	•	•	•	•	•	•	•	•	•	•	•	•	•	•	•	•	•	•	•
	LC440656.1 ZM	•	•	•	•	•	•	•	•	•	•	•	•	•	•	•	•	•	•	•	•	•	•	•	•	•	•	•	•	•	•	•	•	•	•	•	•	•
	LC440657.1 ZM	•	•	•	•	•	•	•	•	•	•	•	•	•	•	•	•	•	•	•	•	•	•	•	•	•	•	•	•	•	•	•	•	•	•	•	•	•
	LC440658.1 ZM	•	•	•	•	•	•	•	•	•	•	•	•	•	•	•	•	•	•	•	•	•	•	•	•	•	•	•	•	•	•	•	•	•	•	•	•	•
	LC440659.1 ZM	•	•	•	•	•	•	•	•	•	•	•	•	•	•	•	•	•	•	•	•	•	•	•	•	•	•	•	•	•	•	•	•	•	•	•	•	•
	LC440660.1 ZM	•	•	•	•	•	•	•	•	•	•	•	•	•	•	•	•	•	•	•	•	•	•	•	•	•	•	•	•	•	•	•	•	•	•	•	•	•
	LC440661.1 ZM	•	•	•	•	•	•	•	•	•	•	•	•	•	•	•	•	•	•	•	•	•	•	•	•	•	•	•	•	•	•	R	•	•	•	•	•	•
	LC440662.1 ZM	•	•	•	•	•	•	•	•	•	•	•	•	•	•	•	•	•	•	•	•	•	•	•	•	•	•	•	•	•	•	•	•	•	•	•	•	•
	LC440663.1 ZM	•	•	•	•	•	•	•	•	•	•	•	•	•	•	•	•	•	•	•	•	Y	•	•	•	•	•	•	•	•	•	•	•	•	•	•	•	•
	LC440664.1 ZM	•	•	•	•	•	•	•	•	•	•	•	•	•	•	•	•	•	•	•	•	•	•	•	•	•	•	•	•	•	•	•	•	•	•	•	•	•
	LC440665.1 ZM	•	•	•	•	•	•	•	•	•	•	•	•	•	•	•	•	•	•	•	•	•	•	•	•	•	•	•	•	•	•	•	•	•	•	•	•	•
	LC440666.1 ZM	•	•	•	•	•	•	•	•	•	•	•	•	•	•	•	•	•	•	•	•	•	•	•	•	•	•	•	•	•	•	•	•	•	•	•	•	•
G4	LC193462.1 ZM	L	I	T	F	S	L	H	K	I	P	D	P	P	Q	P	D	F	P	Q	L	N	S	D	W	V	P	S	V	R	S	W	A	L	L	L	N	Q
SA G4	K1170 SA	•	•	•	•	•	•	•	•	•	•	•	•	•	•	•	•	•	•	•	•	•	•	•	•	•	•	•	•	•	•	•	•	•	•	•	•	•
	K1194 SA	•	•	•	•	•	•	•	•	•	•	•	•	•	•	•	•	•	•	•	•	•	•	•	•	•	•	•	•	•	•	•	•	•	•	•	•	•
	L3401 SA	•	•	•	•	•	•	•	•	•	•	•	•	•	•	•	•	•	•	•	•	•	•	•	•	•	•	•	•	•	•	•	•	•	•	•	•	•
	M1878 SA	•	•	•	•	•	•	•	•	•	•	•	•	•	•	•	•	•	•	•	•	•	•	•	•	•	•	•	•	•	•	•	•	•	•	•	•	•
	P2152 SA	•	•	•	•	•	•	•	•	•	•	•	•	•	•	•	•	•	•	•	•	•	•	•	•	•	•	•	•	•	•	•	•	•	•	•	•	•
	P2677 SA	•	•	•	•	•	•	•	•	•	•	•	•	•	•	•	•	•	•	•	•	•	•	•	•	•	•	•	•	•	•	•	•	•	•	•	•	•
	P591 SA	•	•	•	•	•	•	•	•	•	•	•	•	•	•	•	•	•	•	•	•	•	•	•	•	•	•	•	•	•	•	•	•	•	•	•	•	•

Epitopes and motifs							ND3																															
															Zinc binding site																							
Amino acid position		205	206	207	208	209	210	211	212	213	214	215	216	217	218	219	220	221	222	223	224	225	226	227	228	229	230	231	232	233	234	235	236	237	238	239	240	241
SA G1	M2746 SA	T	A	R	A	F	P	D	C	A	I	C	W	E	P	S	P	P	W	A	P	E	I	L	V	Y	N	K	T	I	S	S	S	G	P	G	L	A
G1	LC440653.1 ZM	•	•	•	•	•	•	•	•	•	•	•	•	•	•	•	•	•	•	•	•	•	•	•	•	•	•	•	•	F	•	•	•	•	•	•	•	•
	LC440654.1 ZM	•	•	•	•	•	•	•	•	•	•	•	•	•	•	•	•	•	•	•	•	•	•	•	•	•	•	•	•	F	•	•	•	•	•	•	•	•
	LC440655.1 ZM	•	•	•	•	•	•	•	•	•	•	•	•	•	•	•	•	•	•	•	•	•	•	•	•	•	•	•	•	F	•	•	•	•	•	•	•	•
	LC440656.1 ZM	•	•	•	•	•	•	•	•	•	•	•	•	•	•	•	•	•	•	•	•	•	•	•	•	•	•	•	•	F	•	•	•	•	•	•	•	•
	LC440657.1 ZM	•	•	•	•	•	•	•	•	•	•	•	•	•	•	•	•	•	•	•	•	•	•	•	•	•	•	•	•	F	•	•	•	•	•	•	•	•
	LC440658.1 ZM	•	•	•	•	•	•	•	•	•	•	•	•	•	•	•	•	•	•	•	•	•	•	•	•	•	•	•	•	F	•	•	•	•	•	•	•	•
	LC440659.1 ZM	•	•	•	•	•	•	•	•	•	•	•	•	•	•	•	•	•	•	•	•	•	•	•	•	•	•	•	•	F	•	•	•	•	•	•	•	•
	LC440660.1 ZM	•	•	•	•	•	•	•	•	•	•	•	•	•	•	•	•	•	•	•	•	•	•	•	•	•	•	•	•	F	•	•	•	•	•	•	•	•
	LC440661.1 ZM	•	•	•	•	•	•	•	•	•	•	•	•	•	•	•	•	•	•	•	•	•	•	•	•	•	•	•	•	F	•	•	•	•	•	•	•	•
	LC440662.1 ZM	•	•	•	•	•	•	•	•	•	•	•	•	•	•	•	•	•	•	•	•	•	•	•	•	•	•	•	•	F	•	•	•	•	•	•	•	•
	LC440663.1 ZM	•	•	•	•	•	•	•	•	•	•	•	•	•	•	•	•	•	•	•	•	•	•	•	•	•	•	•	•	F	•	•	•	•	•	•	•	•
	LC440664.1 ZM	•	•	•	•	•	•	•	•	•	•	•	•	•	•	•	•	•	•	•	•	•	•	•	•	•	•	•	•	F	•	•	•	•	•	•	•	•
	LC440665.1 ZM	•	•	•	•	•	•	•	•	•	•	•	•	•	•	•	•	•	•	•	•	•	•	•	•	•	•	•	•	F	•	•	•	•	•	•	•	•
	LC440666.1 ZM	•	•	•	•	•	•	•	•	•	•	•	•	•	•	•	•	•	•	•	•	•	•	•	•	•	•	•	•	F	•	•	•	•	•	•	•	•
G4	LC193462.1 ZM	T	A	R	A	F	P	D	C	A	I	C	W	E	P	S	P	P	W	A	P	E	I	L	V	Y	N	K	T	I	S	S	S	G	P	G	L	A
SA G4	K1170 SA	•	•	•	•	•	•	•	•	•	•	•	•	•	•	•	•	•	•	•	•	•	•	•	•	•	•	•	•	•	•	•	•	•	•	•	•	•
	K1194 SA	•	•	•	•	•	•	•	•	•	•	•	•	•	•	•	•	•	•	•	•	•	•	•	•	•	•	•	•	•	•	•	•	•	•	•	•	•
	L3401 SA	•	•	•	•	•	•	•	•	•	•	•	•	•	•	•	•	•	•	•	•	•	•	•	•	•	•	•	•	•	•	•	•	•	•	•	•	•
	M1878 SA	•	•	•	•	•	•	•	•	•	•	•	•	•	•	•	•	•	•	•	•	•	•	•	•	•	•	•	•	•	•	•	•	•	•	•	•	•
	P2152 SA	•	•	•	•	•	•	•	•	•	•	•	•	•	•	•	•	•	•	•	•	•	•	•	•	•	•	•	•	•	•	•	•	•	•	•	•	•
	P2677 SA	•	•	•	•	•	•	•	•	•	•	•	•	•	•	•	•	•	•	•	•	•	•	•	•	•	•	•	•	•	•	•	•	•	•	•	•	•
	P591 SA	•	•	•	•	•	•	•	•	•	•	•	•	•	•	•	•	•	•	•	•	•	•	•	•	•	•	•	•	•	•	•	•	•	•	•	•	•

Figure S11 *– Continued.*

		SA G4							SA G1	G1											G2				G4		G6				G9				G10			
		K1170 SA	K1194 SA	L3401 SA	M1878 SA	P2152 SA	P2677 SA	P591 SA	M2746 SA	AB934282.1 JP	AP018006.1 JP	AP018007.1 JP	AP019589.1 JP	D00647.1 AU	EF600696.1 US	HE967301.1 UY	K02120.1 JP	LC080652.1 PY	LC164085.1 JP	MH170028.1 VN	AF257515.1 AR	FJ914764.1 AR	LC080654.1 PE	LC080655.1 PY	AF033818.1 US	KT122858.1 BE	LC080656.1 PY	LC080658.1 PY	MF580992.1 CN	MH170029.1 VN	LC080662.1 BO	LC080663.1 BO	LC080667.1 BO	LC080671.1 BO	LC154848.1 MM	LC154849.1 MM	MF580994.1 CN	MF580995.1 CN
SA G4	K1170 SA		99.75	99.49	99.24	99.49	100	99.75	97.71	97.71	96.95	97.47	97.46	94.15	97.71	97.71	82.87	97.46	98.22	97.46	96.18	97.20	97.96	97.96	98.47	98.47	98.47	98.22	96.18	98.22	97.96	97.71	97.96	97.96	96.95	97.46	94.66	97.20
	K1194 SA	99.58		99.49	99.24	99.49	99.75	99.49	97.46	97.46	96.69	97.20	97.20	93.89	97.46	97.46	82.62	97.20	97.96	97.20	95.93	96.95	97.71	97.71	98.22	98.22	98.22	97.96	96.44	97.96	97.71	97.46	97.71	97.71	96.69	97.20	94.40	96.95
	L3401 SA	99.49	99.41		99.75	100	99.49	99.24	97.71	97.71	96.95	97.46	97.46	93.64	97.71	97.71	82.87	97.46	98.22	97.46	96.18	97.20	97.96	97.96	97.96	97.96	97.96	97.71	95.93	97.71	97.96	97.71	97.96	97.96	96.44	96.95	94.15	96.96
	M1878 SA	99.32	99.24	99.83		99.75	99.24	98.98	97.46	97.96	96.96	97.71	97.20	93.38	97.46	97.46	83.12	97.20	97.96	97.20	95.93	96.95	97.71	97.71	98.22	97.71	97.71	97.46	95.67	97.96	97.71	97.46	97.71	97.71	96.18	96.69	94.15	96.44
	P2152 SA	99.41	99.32	99.92	99.75		99.49	99.24	97.71	97.71	96.95	97.46	97.46	93.64	97.71	97.71	82.87	97.46	98.22	97.46	96.18	97.20	97.96	97.96	97.96	97.96	97.96	97.71	95.93	97.71	97.96	97.71	97.96	97.96	96.44	96.95	94.15	96.96
	P2677 SA	99.83	99.75	99.49	99.32	99.41		99.75	97.71	97.71	96.95	97.46	97.46	94.15	97.71	97.71	82.87	97.46	98.22	97.46	96.18	97.20	97.96	97.96	98.47	98.47	98.47	98.22	96.18	98.22	97.96	97.71	97.96	97.96	96.95	97.46	94.66	97.20
	P591 SA	99.75	99.66	99.41	99.24	99.32	99.92		97.46	97.46	96.69	97.20	97.20	93.89	97.46	97.46	82.62	97.20	97.96	97.20	95.93	96.95	97.71	97.71	98.22	98.22	98.22	97.96	95.93	97.96	97.71	97.46	97.71	97.71	96.69	97.20	94.40	96.95
SA G1	M2746 SA	96.11	96.19	96.19	96.02	96.28	96.28	96.19		99.49	99.24	99.24	99.75	93.64	99.24	99.49	84.89	99.24	99.49	99.75	96.95	97.96	98.73	98.73	97.96	97.71	98.22	98.22	96.18	97.96	98.73	98.47	98.73	98.73	97.20	97.71	94.91	97.46
G1	AB934282.1 JP	95.94	96.02	96.02	96.02	96.02	96.11	96.02	99.24		98.73	99.75	99.24	93.64	99.24	99.49	84.89	99.24	99.49	99.24	96.95	97.96	98.73	98.73	98.47	97.71	98.22	98.22	96.18	98.47	98.73	98.47	98.73	98.73	97.20	97.71	95.17	97.46
	AP018006.1 JP	95.60	95.69	95.69	95.52	95.69	95.77	95.69	99.41	98.82		98.47	98.98	92.88	98.47	98.73	84.38	98.98	98.73	98.98	96.18	97.20	97.96	97.96	97.20	96.95	97.46	97.46	95.67	97.20	97.96	97.71	97.96	97.96	96.44	96.95	94.15	96.96
	AP018007.1 JP	96.28	96.36	96.36	96.36	96.36	96.45	96.36	99.24	99.32	98.82		98.98	93.83	98.98	99.24	84.63	98.98	99.24	98.98	96.96	97.71	98.47	98.47	98.22	97.46	97.96	97.71	95.93	98.22	98.47	98.22	98.47	98.47	97.20	97.71	95.17	97.46
	AP019589.1 JP	95.69	95.77	95.77	95.60	95.77	95.85	95.77	99.49	98.90	99.24	98.90		93.38	99.49	99.24	84.89	98.98	99.24	99.49	96.69	97.71	98.47	98.47	97.71	97.46	97.96	97.96	95.93	97.71	98.47	98.22	98.47	98.47	96.95	97.46	94.66	97.20
	D00647.1 AU	95.69	95.78	95.61	95.44	95.61	95.86	95.78	94.43	94.34	94.00	94.68	94.09		93.64	93.64	86.40	93.38	93.89	93.38	93.38	92.88	93.64	93.64	94.40	94.15	94.66	94.40	92.37	94.15	93.64	93.38	93.64	93.64	92.88	93.38	91.60	93.13
	EF600696.1 US	96.11	96.19	96.19	96.02	96.19	96.28	96.19	99.41	99.32	98.98	99.32	99.24	94.68		99.24	84.38	98.98	99.49	98.98	96.95	97.96	98.73	98.73	98.22	97.71	98.22	97.96	96.18	97.96	98.73	98.47	98.73	98.73	97.47	97.96	95.17	97.71
	HE967301.1 UY	96.02	96.11	96.11	95.94	96.11	96.19	96.11	99.32	99.24	98.90	99.24	98.98	94.51	99.41		84.38	99.75	99.49	99.24	96.95	97.96	98.73	98.73	97.96	97.71	98.22	97.96	96.18	97.96	98.73	98.47	98.73	98.73	97.20	97.71	94.91	97.46
	K02120.1 JP	94.77	94.69	94.86	94.86	94.86	94.77	94.69	98.23	97.98	97.89	97.98	97.98	93.33	98.06	97.89		84.38	84.38	84.63	83.88	82.87	83.63	83.63	83.38	82.62	83.12	83.12	81.36	83.38	83.63	83.38	83.63	83.63	82.12	82.62	81.11	82.37
	LC080652.1 PY	96.02	96.11	96.11	95.94	96.11	96.19	96.11	99.32	99.24	99.07	99.24	98.98	94.43	99.41	99.83	97.89		99.24	98.98	96.69	97.71	98.47	98.47	97.71	97.46	97.96	97.71	95.93	97.71	98.47	98.22	98.47	98.47	96.95	97.46	94.66	97.20
	LC164085.1 JP	96.28	96.19	96.36	96.19	96.36	96.28	96.19	99.49	99.24	99.07	99.24	99.15	94.51	99.41	99.32	98.23	99.32		99.24	97.46	98.47	99.24	99.24	98.22	97.71	98.47	98.22	96.44	98.22	99.24	98.98	99.24	99.24	97.46	97.96	95.17	97.71
	MH170028.1 VN	95.85	95.94	95.94	95.77	95.94	96.02	95.94	99.66	99.07	99.41	99.07	99.49	94.26	99.24	99.15	98.06	99.15	99.32		97.20	98.22	98.98	98.98	97.71	97.46	97.96	97.96	95.93	97.71	98.98	98.73	98.98	98.98	97.46	97.46	94.66	97.20
G2	AF257515.1 AR	95.35	95.44	95.44	95.27	95.44	95.52	95.44	96.03	96.03	95.60	96.28	95.69	94.18	96.11	96.20	94.61	96.20	96.11	96.03		98.47	98.22	97.69	96.18	96.18	96.95	96.69	94.91	96.69	98.22	97.96	98.22	98.22	96.44	96.44	94.15	96.18
	FJ914764.1 AR	95.60	95.69	95.69	95.52	95.69	95.77	95.69	96.28	96.28	95.85	96.53	95.94	94.26	96.36	96.45	94.86	96.45	96.36	96.28	99.58		99.24	98.98	97.20	97.20	97.96	97.71	95.93	97.71	99.24	98.98	99.24	99.24	97.46	97.46	94.66	97.20
	LC080654.1 PE	95.85	95.94	95.94	95.77	95.94	96.02	95.94	96.36	96.36	96.36	96.62	96.02	94.68	96.45	96.53	94.94	96.53	96.45	96.36	98.99	99.24		99.75	97.96	97.96	98.22	97.96	96.18	97.96	100	99.75	100	100	97.71	97.71	94.91	97.46
	LC080655.1 PY	95.77	95.85	95.85	95.69	95.85	95.94	95.85	96.62	96.62	96.19	96.70	96.28	94.43	96.70	96.79	95.19	96.79	96.70	96.62	99.24	99.49	99.41		97.96	97.96	98.22	97.96	96.18	97.96	99.75	99.49	99.75	99.75	97.46	97.46	94.66	97.20
G4	AF033818.1 US	97.46	97.38	97.38	97.38	97.29	97.46	97.38	95.60	95.60	95.26	95.94	95.35	95.52	95.77	95.52	94.77	95.52	95.94	95.52	95.18	95.43	95.69	95.60		98.47	98.73	98.47	96.44	98.98	97.96	97.71	97.96	97.96	97.46	97.96	95.42	97.71
	KT122858.1 BE	98.14	98.05	98.05	97.88	97.97	98.14	98.05	96.28	96.11	95.77	96.45	95.85	95.95	96.28	96.19	94.94	96.19	96.45	96.02	95.77	96.02	96.28	96.19	97.80		98.47	98.22	96.18	98.22	97.96	97.71	97.96	97.96	96.95	97.46	94.66	97.20
G6	LC080656.1 PY	96.19	96.11	96.11	95.94	96.11	96.19	96.11	95.26	95.18	94.84	95.35	94.92	97.30	95.52	95.26	94.18	95.26	95.52	95.09	94.84	95.09	95.35	95.26	96.45	96.53		99.75	97.71	99.49	98.22	97.96	98.22	98.22	98.22	98.73	95.93	98.47
	LC080658.1 PY	95.85	95.94	95.77	95.77	95.77	95.85	95.77	95.09	95.01	94.67	95.18	94.75	96.11	95.18	95.09	94.01	95.09	95.35	94.92	94.59	94.84	95.09	95.01	95.85	96.19	97.63		97.46	99.24	97.96	97.71	97.96	97.96	97.96	98.47	95.67	98.22
	MF580992.1 CN	95.18	95.43	95.09	95.09	95.09	95.18	95.09	94.33	94.25	93.91	94.50	93.99	95.44	94.42	94.33	93.25	94.33	94.59	94.16	93.91	94.16	94.42	94.25	95.18	95.52	96.87	99.07		97.20	96.18	95.93	96.18	96.18	96.44	96.44	93.64	96.18
	MH170029.1 VN	95.43	95.35	95.35	95.35	95.35	95.43	95.35	94.33	94.59	93.91	94.75	94.08	96.88	94.75	94.33	93.42	94.33	94.59	94.16	94.42	94.59	94.84	94.59	96.02	95.77	98.39	96.87	96.19		97.96	97.71	97.96	97.96	97.96	98.47	95.93	98.22
G9	LC080662.1 BO	96.36	96.28	96.45	96.28	96.45	96.36	96.28	96.87	96.87	96.45	97.12	96.53	95.10	96.95	96.87	95.62	96.87	97.12	96.87	97.30	97.55	97.80	97.72	96.19	96.70	95.94	95.69	95.01	95.26		99.75	100	100	97.71	97.71	94.91	97.46
	LC080663.1 BO	96.28	96.19	96.36	96.19	96.36	96.28	96.19	96.79	96.79	96.36	97.04	96.45	95.02	96.87	96.79	95.53	96.79	97.04	96.79	97.21	97.46	97.72	97.63	96.11	96.62	95.85	95.60	94.92	95.18	99.92		99.75	99.75	97.46	97.46	94.66	97.20
	LC080667.1 BO	96.19	96.11	96.28	96.11	96.28	96.19	96.11	96.70	96.70	96.28	96.95	96.36	94.93	96.79	96.70	95.45	96.70	96.95	96.70	97.13	97.38	97.63	97.55	96.02	96.53	95.77	95.52	94.84	95.09	99.83	99.75		100	97.71	97.71	94.91	97.46
	LC080671.1 BO	96.11	96.02	96.19	96.02	96.19	96.11	96.02	96.62	96.62	96.19	96.87	96.28	94.85	96.70	96.62	95.36	96.62	96.87	96.62	97.04	97.29	97.55	97.46	95.94	96.45	95.69	95.43	94.75	95.01	99.75	99.66	99.92		97.71	97.71	94.91	97.46
G10	LC154848.1 MM	95.18	95.09	95.09	94.92	95.09	95.18	95.09	94.50	94.33	94.08	94.75	94.25	95.52	94.67	94.42	93.42	94.42	94.67	94.50	94.42	94.50	94.59	94.50	95.18	95.52	95.94	95.60	94.92	95.60	95.09	95.01	94.92	94.84		99.49	96.69	99.24
	LC154849.1 MM	95.09	95.01	95.01	94.84	95.01	95.09	95.01	94.59	94.42	94.16	94.84	94.33	95.35	94.75	94.50	93.51	94.50	94.75	94.42	94.34	94.42	94.59	94.42	95.09	95.43	95.85	95.52	94.84	95.52	95.01	94.92	94.84	94.75	98.90		97.20	99.75
	MF580994.1 CN	94.16	94.08	94.08	93.91	94.08	94.16	94.08	93.65	93.49	93.23	93.91	93.40	94.43	93.82	93.57	92.58	93.57	93.82	93.49	93.41	93.49	93.65	93.49	94.16	94.50	94.92	94.59	93.91	94.59	94.08	93.99	93.91	93.82	97.97	99.07		96.95
	MF580995.1 CN	94.92	94.84	94.84	94.67	94.84	94.92	94.84	94.42	94.25	93.99	94.67	94.16	95.19	94.59	94.33	93.34	94.33	94.59	94.25	94.17	94.25	94.42	94.25	94.92	95.26	95.69	95.35	94.67	95.35	94.84	94.75	94.67	94.59	98.73	99.83	98.90	

81.0 82.0 83.0 84.0 85.0 86.0 87.0 88.0 89.0 90.0 91.0 92.0 93.0 94.0 95.0 96.0 97.0 98.0 99.0 100 %

Figure S12. Pairwise percent identity of BLV full-length Gag nucleotide (1,182 bp) and amino acid (393 amino acids) sequences between the South African isolates and 29 selected isolates from other geographic regions worldwide. Lower matrix shows percent identity of nucleotide sequences and the upper matrix shows percent identity of amino acid sequences. The country of origin is indicated by 2-letter country codes (See Table S2). SA, South Africa.

		SA G4							SA G1	G1											G2				G4		G6				G9				G10			
		K1170 SA	K1194 SA	L3401 SA	M1878 SA	P2152 SA	P2677 SA	P591 SA	M2746 SA	AB934282.1 JP	AP018006.1 JP	AP018007.1 JP	AP019589.1 JP	D00647.1 AU	EF600696.1 US	HE967301.1 UY	K02120.1 JP	LC080652.1 PY	LC164085.1 JP	MH170028.1 VN	AF257515.1 AR	FJ914764.1 AR	LC080654.1 PE	LC080655.1 PY	AF033818.1 US	KT122858.1 BE	LC080656.1 PY	LC080658.1 PY	MF580992.1 CN	MH170029.1 VN	LC080662.1 BO	LC080663.1 BO	LC080667.1 BO	LC080671.1 BO	LC154848.1 MM	LC154849.1 MM	MF580994.1 CN	MF580995.1 CN
SA G4	K1170 SA		1	2	3	2	0	1	9	9	12	10	10	23	9	9	67	10	7	10	15	11	8	8	6	6	6	7	15	7	8	9	8	8	12	10	21	11
	K1194 SA	5		2	3	2	1	2	10	10	13	11	11	24	10	10	68	11	8	11	16	12	9	9	7	7	7	8	14	8	9	10	9	9	13	11	22	12
	L3401 SA	6	7		1	0	2	3	9	9	12	10	10	25	9	9	67	10	7	10	15	11	8	8	8	8	8	9	16	9	8	9	8	8	14	12	23	12
	M1878 SA	8	9	2		1	3	4	10	8	12	9	11	26	10	10	66	11	8	11	16	12	9	9	7	9	9	10	17	8	9	10	9	9	15	13	23	14
	P2152 SA	7	8	1	3		2	3	9	9	12	10	10	25	9	9	67	10	7	10	15	11	8	8	8	8	8	9	16	9	8	9	8	8	14	12	23	12
	P2677 SA	2	3	6	8	7		1	9	9	12	10	10	23	9	9	67	10	7	10	15	11	8	8	6	6	6	7	15	7	8	9	8	8	12	10	21	11
	P591 SA	3	4	7	9	8	1		10	10	13	11	11	24	10	10	68	11	8	11	16	12	9	9	7	7	7	8	16	8	9	10	9	9	13	11	22	12
SA G1	M2746 SA	46	45	45	47	44	44	45		2	3	3	1	25	3	2	59	3	2	1	12	8	5	5	8	9	7	7	15	8	5	6	5	5	11	9	20	10
G1	AB934282.1 JP	48	47	47	47	47	46	47	9		5	1	3	25	3	2	59	3	2	3	12	8	5	5	6	9	7	7	15	6	5	6	5	5	11	9	19	10
	AP018006.1 JP	52	51	51	53	51	50	51	7	14		6	4	28	6	5	61	4	5	4	15	11	8	8	11	12	10	10	17	11	8	9	8	8	14	12	23	12
	AP018007.1 JP	44	43	43	43	43	42	43	9	8	14		4	24	4	3	60	4	3	4	12	9	6	6	7	10	8	9	16	7	6	7	6	6	11	9	19	10
	AP019589.1 JP	51	50	50	52	50	49	50	6	13	9	13		26	2	3	59	4	3	2	13	9	6	6	9	10	8	8	16	9	6	7	6	6	12	10	21	11
	D00647.1 AU	51	50	52	54	52	49	50	66	67	71	63	70		25	25	53	26	24	26	26	28	25	25	22	23	21	22	30	23	25	26	25	25	28	26	33	27
	EF600696.1 US	46	45	45	47	45	44	45	7	8	12	8	9	63		3	61	4	2	4	12	8	5	5	7	9	7	8	15	8	5	6	5	5	10	8	19	9
	HE967301.1 UY	47	46	46	48	46	45	46	8	9	13	9	12	65	7		61	1	2	3	12	8	5	5	8	9	7	8	15	8	5	6	5	5	11	9	20	10
	K02120.1 JP	62	63	61	61	61	62	63	21	24	25	24	24	79	23	25		61	61	60	63	67	64	64	65	68	66	66	73	65	64	65	64	64	70	68	74	69
	LC080652.1 PY	47	46	46	48	46	45	46	8	9	11	9	12	66	7	2	25		3	4	13	9	6	6	9	10	8	9	16	9	6	7	6	6	12	10	21	11
	LC164085.1 JP	44	45	43	45	43	44	45	6	9	11	9	10	65	7	8	21	8		3	10	6	3	3	7	7	6	7	14	7	3	4	3	3	10	8	19	9
	MH170028.1 VN	49	48	48	50	48	47	48	4	11	7	11	6	68	9	10	23	10	8		11	7	4	4	9	10	8	8	16	9	4	5	4	4	10	10	21	11
G2	AF257515.1 AR	55	54	54	56	54	53	54	47	47	52	44	51	69	46	45	64	45	46	47		6	7	9	15	15	12	13	20	13	7	8	7	7	14	14	23	15
	FJ914764.1 AR	52	51	51	53	51	50	51	44	44	49	41	48	68	43	42	61	42	43	44	5		3	4	11	11	8	9	16	9	3	4	3	3	10	10	21	11
	LC080654.1 PE	49	48	48	50	48	47	48	43	43	48	40	47	63	42	41	60	41	42	43	12	9		1	8	8	7	8	15	8	0	1	0	0	9	9	20	10
	LC080655.1 PY	50	49	49	51	49	48	49	40	40	45	39	44	66	39	38	57	38	39	40	9	6	7		8	8	7	8	15	8	1	2	1	1	10	10	21	11
G4	AF033818.1 US	30	31	31	31	32	30	31	52	52	56	48	55	53	50	53	62	53	48	53	57	54	51	52		6	5	6	14	4	8	9	8	8	10	8	18	9
	KT122858.1 BE	22	23	23	25	24	22	23	44	46	50	42	49	48	44	45	60	45	42	47	50	47	44	45	26		6	7	15	7	8	9	8	8	12	10	21	11
G6	LC080656.1 PY	45	46	46	48	46	45	46	56	57	61	55	60	32	53	56	69	56	53	58	61	58	55	56	42	41		1	9	2	7	8	7	7	7	5	16	6
	LC080658.1 PY	49	48	50	50	50	49	50	58	59	63	57	62	46	57	58	71	58	55	60	64	61	58	59	49	45	28		10	3	8	9	8	8	8	6	17	7
	MF580992.1 CN	57	54	58	58	58	57	58	67	68	72	65	71	54	66	67	80	67	64	69	72	69	66	68	57	53	37	11		11	15	16	15	15	14	14	25	15
	MH170029.1 VN	54	55	55	55	55	54	55	67	64	72	62	70	37	62	67	78	67	64	69	66	64	61	64	47	50	19	37	45		8	9	8	8	8	6	16	7
G9	LC080662.1 BO	43	44	42	44	42	43	44	37	37	42	34	41	58	36	37	52	37	34	37	32	29	26	27	45	39	48	51	59	56		1	0	0	9	9	20	10
	LC080663.1 BO	44	45	43	45	43	44	45	38	38	43	35	42	59	37	38	53	38	35	38	33	30	27	28	46	40	49	52	60	57	1		1	1	10	10	21	11
	LC080667.1 BO	45	46	44	46	44	45	46	39	39	44	36	43	60	38	39	54	39	36	39	34	31	28	29	47	41	50	53	61	58	2	3		0	9	9	20	10
	LC080671.1 BO	46	47	45	47	45	46	47	40	40	45	37	44	61	39	40	55	40	37	40	35	32	29	30	48	42	51	54	62	59	3	4	1		9	9	20	10
G10	LC154848.1 MM	57	58	58	60	58	57	58	65	67	70	62	68	53	63	66	78	66	63	65	66	65	64	65	57	53	48	52	60	52	58	59	60	61		2	13	3
	LC154849.1 MM	58	59	59	61	59	58	59	64	66	69	61	67	55	62	65	77	65	62	66	67	66	64	66	58	54	49	53	61	53	59	60	61	62	13		11	1
	MF580994.1 CN	69	70	70	72	70	69	70	75	77	80	72	78	66	73	76	88	76	73	77	78	77	75	77	69	65	60	64	72	64	70	71	72	73	24	11		12
	MF580995.1 CN	60	61	61	63	61	60	61	66	68	71	63	69	57	64	67	79	67	64	68	69	68	66	68	60	56	51	55	63	55	61	62	63	64	15	2	13	

0 5 10 15 20 25 30 35 40 45 50 55 60 65 70 75 80 85 90

Figure S13. Pairwise comparison of BLV full-length Gag nucleotide (1,182 bp) and amino acid (393 amino acids) differences between the South African isolates and 29 selected isolates from other geographic regions worldwide. Lower matrix shows the number of nucleotide differences and the upper matrix shows the number of amino acid differences. The country of origin is indicated by 2-letter country codes (See Table S2). SA, South Africa.

Table S4. Mean nucleotide and amino acid distances in the BLV full-length Env sequences within (intragenotype) and between (intergenotype) BLV strains from South Africa and other geographic regions worldwide.

	SA G1	SA G4	G1	G2	G3	G4	G5	G6	G7	G9	G10
SA G1	N/A	0.0315±0.0080	0.0063±0.0022	0.0309±0.0064	0.0245±0.0066	0.0279±0.0068	0.0346±0.0073	0.0388±0.0072	0.0279±0.0064	0.0225±0.0063	0.0434±0.0075
SA G4	0.0387±0.0054	0.0013±0.0050<br>0.0011 ±0.0008	0.0330±0.0073	0.0361±0.0075	0.0292±0.0072	0.0141±0.0042	0.0332±0.0070	0.0319±0.0061	0.0280±0.0064	0.0288±0.0073	0.0362±0.0067
G1	0.0082±0.0018	0.0369±0.0051	0.0069±0.0007<br>0.0079 ±0.0013	0.0327±0.0061	0.0261±0.0062	0.0293±0.0064	0.0359±0.0070	0.0404±0.0068	0.0294±0.0060	0.0243±0.0059	0.0450±0.0071
G2	0.0346±0.0047	0.0383±0.0050	0.0328±0.041	0.0105±0.0017<br>0.0204±0.0046	0.0193±0.0044	0.0326±0.0064	0.0445±0.0077	0.0454±0.0072	0.0357±0.0067	0.0188±0.0045	0.0494±0.0075
G3	0.0304±0.0044	0.0342±0.0048	0.0302±0.0041	0.0255±0.0038	0.0049±0.0011<br>0.0031±0.0017	0.0256±0.0062	0.0375±0.0075	0.0387±0.0071	0.0291±0.0067	0.0138±0.0047	0.0432±0.0073
G4	0.0383±0.0049	0.0138±0.0022	0.0365±0.0044	0.0374±0.0045	0.0335±0.0043	0.0129±0.0017<br>0.0097±0.0026	0.0331±0.0068	0.0323±0.0057	0.0234±0.0059	0.0255±0.0061	0.0372±0.0062
G5	0.0423±0.0052	0.0377±0.0049	0.0397±0.0048	0.0462±0.0055	0.0441±0.0053	0.0389±0.0045	0.0162±0.0023<br>0.0164±0.0038	0.0443±0.0072	0.0345±0.0066	0.0381±0.0074	0.0487±0.0077
G6	0.0470±0.0051	0.0408±0.0047	0.0456±0.0047	0.0496±0.0050	0.0447±0.0048	0.0421±0.0043	0.0492±0.0048	0.0266±0.0030<br>0.0248±0.0042	0.0315±0.0056	0.0373±0.0069	0.0309±0.0050
G7	0.0394±0.0054	0.0324±0.0051	0.0377±0.0050	0.0423±0.0055	0.0397±0.0052	0.0341±0.0049	0.0445±0.0054	0.0454±0.049	0.0082±0.0018<br>0.0085±0.0030	0.0291±0.0067	0.0353±0.0061
G9	0.0313±0.0046	0.0347±0.0052	0.0312±0.0043	0.0239±0.0036	0.0224±0.0035	0.0356±0.0047	0.0448±0.0055	0.0446±0.0050	0.0410±0.0056	0.0016±0.0006<br>0.0014±0.0009	0.0408±0.0070
G10	0.0490±0.0053	0.0436±0.0049	0.0479±0.0049	0.0513±0.0052	0.0457±0.0049	0.0448±0.0046	0.0519±0.0049	0.0288 ±0.0033	0.0486±0.0052	0.0469±0.0052	0.0273±0.0033<br>0.0320 ±0.0054

Genetic distances in each column are shown with standard errors. The lower matrix shows intergenotype nucleotide distance and the upper matrix shows intergenotype amino acid distance. Diagonal columns (highlighted in green) show intragenotype nucleotide (top) and amino acid (bottom) distances. Mean intergenotype nucleotide and amino acid distances between G1 and G4 South African isolates and the G1 and G4 global isolates are highlighted in yellow. N/A, not applicable.

References

1. Camargos, M.F.; Stancek, D.; Rocha, M.A.; Lessa, L.M.; Reis, J.K.; Leite, R.C. Partial sequencing of *env* gene of bovine leukaemia virus from Brazilian samples and phylogenetic analysis. *Journal of veterinary medicine. B, Infectious diseases and veterinary public health* **2002**, *49*, 325-331.
2. Camargos, M.F.; Pereda, A.; Stancek, D.; Rocha, M.A.; dos Reis, J.K.; Greiser-Wilke, I.; Leite, R.C. Molecular characterization of the *env* gene from Brazilian field isolates of Bovine leukemia virus. *Virus Genes* **2007**, *34*, 343-350, doi:10.1007/s11262-006-0011-x.
3. Murakami, H.; Uchiyama, J.; Suzuki, C.; Nikaido, S.; Shibuya, K.; Sato, R.; Maeda, Y.; Tomioka, M.; Takeshima, S.N.; Kato, H., et al. Variations in the viral genome and biological properties of bovine leukemia virus wild-type strains. *Virus Res* **2018**, *253*, 103-111, doi:10.1016/j.virusres.2018.06.005.
4. Johnston, E.R.; Albritton, L.M.; Radke, K. Envelope Proteins Containing Single Amino Acid Substitutions Support a Structural Model of the Receptor-Binding Domain of Bovine Leukemia Virus Surface Protein. *Journal of Virology* **2002**, *76*, 10861-10872, doi:10.1128/jvi.76.21.10861-10872.2002.
5. Coulston, J.; Naif, H.; Brandon, R.; Kumar, S.; Khan, S.; Daniel, R.C.; Lavin, M.F. Molecular cloning and sequencing of an Australian isolate of proviral bovine leukaemia virus DNA: comparison with other isolates. *J Gen Virol* **1990**, *71* 1737-1746, doi:10.1099/0022-1317-71-8-1737.
6. Zhao, X.; Buehring, G.C. Natural genetic variations in bovine leukemia virus envelope gene: possible effects of selection and escape. *Virology* **2007**, *366*, 150-165, doi:10.1016/j.virol.2007.03.058.
7. Derse, D.; Diniak, A.J.; Casey, J.W.; Deininger, P.L. Nucleotide sequence and structure of integrated bovine leukemia virus long terminal repeats. *Virology* **1985**, *141*, 162-166.
8. Hemmatzadeh, F. Sequencing and phylogenetic analysis of gp51 gene of bovine leukaemia virus in Iranian isolates. *Vet Res Commun* **2007**, *31*, 783-789, doi:10.1007/s11259-007-0012-9.
9. Rodriguez, S.M.; Golemba, M.D.; Campos, R.H.; Trono, K.; Jones, L.R. Bovine leukemia virus can be classified into seven genotypes: evidence for the existence of two novel clades. *J Gen Virol* **2009**, *90*, 2788-2797, doi:10.1099/vir.0.011791-0.
10. Sagata, N.; Yasunaga, T.; Tsuzuku-Kawamura, J.; Ohishi, K.; Ogawa, Y.; Ikawa, Y. Complete nucleotide sequence of the genome of bovine leukemia virus: its evolutionary relationship to other retroviruses. *Proc Natl Acad Sci U S A* **1985**, *82*, 677-681.
11. Yang, Y.; Kelly, P.J.; Bai, J.; Zhang, R.; Wang, C. First molecular characterization of bovine leukemia virus infections in the caribbean. *PLoS One* **2016**, *11*, e0168379, doi:10.1371/journal.pone.0168379.
12. Ochirkhuu, N.; Konnai, S.; Odbileg, R.; Nishimori, A.; Okagawa, T.; Murata, S.; Ohashi, K. Detection of bovine leukemia virus and identification of its genotype in Mongolian cattle. *Arch Virol* **2016**, *161*, 985-991, doi:10.1007/s00705-015-2676-8.
13. Polat, M.; Takeshima, S.N.; Hosomichi, K.; Kim, J.; Miyasaka, T.; Yamada, K.; Arainga, M.; Murakami, T.; Matsumoto, Y.; de la Barra Diaz, V., et al. A new genotype of bovine leukemia virus in South America identified by NGS-based whole genome sequencing and molecular evolutionary genetic analysis. *Retrovirology* **2016**, *13*, 4, doi:10.1186/s12977-016-0239-z.
14. Murakami, H.; Uchiyama, J.; Nikaido, S.; Sato, R.; Sakaguchi, M.; Tsukamoto, K. Inefficient viral replication of bovine leukemia virus induced by spontaneous deletion mutation in the G4 gene. *J Gen Virol* **2016**, *97*, 2753-2762, doi:10.1099/jgv.0.000583.
15. Phiri., M.M.; Kaimoyo., E.; Changula., K.; Silwamba., I.; Chambaro., H.M.; Kapila., P.; Kajihara., M.; Simuunza., M.; Muma., J.B.; Pandey., G.S., et al. Molecular detection and characterization of genotype 1 bovine leukemia virus from beef cattle in the traditional sector in Zambia. *Archives of virology* **2019**, *164*, 2531–2536, doi:https://doi.org/10.1007/s00705-019-04350-6.
16. Dao, T.D.; Bui, V.N.; Omatsu, T.; Katayama, Y.; Mizutani, T.; Ogawa, H.; Imai, K. Application of the SureSelect target enrichment system for next-generation sequencing to obtain the

complete genome sequence of bovine leukemia virus. *Arch Virol* **2018**, *163*, 3155-3159, doi:10.1007/s00705-018-3957-9.

17. Dube, S.; Dolcini, G.; Abbott, L.; Mehta, S.; Dube, D.; Gutierrez, S.; Ceriani, C.; Esteban, E.; Ferrer, J.; Poiesz, B. The complete genomic sequence of a BLV strain from a Holstein cow from Argentina. *Virology* **2000**, *277*, 379-386, doi:10.1006/viro.2000.0622.
18. Dube, S.; Abbott, L.; Dube, D.K.; Dolcini, G.; Gutierrez, S.; Ceriani, C.; Juliarena, M.; Ferrer, J.; Perzova, R.; Poiesz, B.J. The complete genomic sequence of an in vivo low replicating BLV strain. *Virol J* **2009**, *6*, 120, doi:10.1186/1743-422X-6-120.
19. Lee, E.; Kim, E.J.; Joung, H.K.; Kim, B.H.; Song, J.Y.; Cho, I.S.; Lee, K.K.; Shin, Y.K. Sequencing and phylogenetic analysis of the gp51 gene from Korean bovine leukemia virus isolates. *Virol J* **2015**, *12*, 64, doi:10.1186/s12985-015-0286-4.
20. Willems, L.; Gatot, J.S.; Mammerickx, M.; Portetelle, D.; Burny, A.; Kerkhofs, P.; Kettmann, R. The YXXL signalling motifs of the bovine leukemia virus transmembrane protein are required for in vivo infection and maintenance of high viral loads. *J Virol* **1995**, *69*, 4137-4141.
21. Felmer, R.; Munoz, G.; Zuniga, J.; Recabal, M. Molecular analysis of a 444 bp fragment of the bovine leukaemia virus gp51 *env* gene reveals a high frequency of non-silent point mutations and suggests the presence of two subgroups of BLV in Chile. *Veterinary microbiology* **2005**, *108*, 39-47, doi:10.1016/j.vetmic.2005.04.005.
22. Rola-Luszczak, M.; Pluta, A.; Olech, M.; Donnik, I.; Petropavlovskiy, M.; Gerilovych, A.; Vinogradova, I.; Choudhury, B.; Kuzmak, J. The molecular characterization of bovine leukaemia virus isolates from Eastern Europe and Siberia and its impact on phylogeny. *PLoS One* **2013**, *8*, e58705, doi:10.1371/journal.pone.0058705.
23. Rice, N.R.; Stephens, R.M.; Couez, D.; Deschamps, J.; Kettmann, R.; Burny, A.; Gilden, R.V. The nucleotide sequence of the *env* gene and post-*env* region of bovine leukemia virus. *Virology* **1984**, *138*, 82-93, doi:https://doi.org/10.1016/0042-6822(84)90149-1.
24. Pandey, G.S.; Simulundu, E.; Mwiinga, D.; Samui, K.L.; Mweene, A.S.; Kajihara, M.; Mangani, A.; Mwenda, R.; Ndebe, J.; Konnai, S., et al. Clinical and subclinical bovine leukemia virus infection in a dairy cattle herd in Zambia. *Archives of virology* **2017**, *162*, 1051-1056, doi:10.1007/s00705-016-3205-0.
25. Mamoun, R.Z.; Morisson, M.; Rebeyrotte, N.; Busetta, B.; Couez, D.; Kettmann, R.; Hospital, M.; Guillemain, B. Sequence variability of bovine leukemia virus *env* gene and its relevance to the structure and antigenicity of the glycoproteins. *J Virol* **1990**, *64*, 4180-4188.
26. Yang, Y.; Chen, L.; Dong, M.; Huang, W.; Hao, X.; Peng, Y.; Gong, Z.; Qin, A.; Shang, S.; Yang, Z. Molecular characterization of bovine leukemia virus reveals existence of genotype 4 in Chinese dairy cattle. *Virol J* **2019**, *16*, 108, doi:10.1186/s12985-019-1207-8.
27. Fechner, H.; Blankenstein, P.; Looman, A.C.; Elwert, J.; Geue, L.; Albrecht, C.; Kurg, A.; Beier, D.; Marquardt, O.; Ebner, D. Provirus variants of the bovine leukemia virus and their relation to the serological status of naturally infected cattle. *Virology* **1997**, *237*, 261-269, doi:10.1006/viro.1997.8784.
28. Gautam, S.; Mishra, N.; Kalaiyarasu, S.; Jhade, S.K.; Sood, R. Molecular characterization of bovine leukaemia virus (BLV) strains reveals existence of genotype 6 in cattle in India with evidence of a new subgenotype. *Transboundary and emerging diseases* **2018**, *65*, 1968-1978, doi:10.1111/tbed.12979.
29. Wang, M.; Wang, Y.; Baloch, A.R.; Pan, Y.; Xu, F.; Tian, L.; Zeng, Q. Molecular epidemiology and characterization of bovine leukemia virus in domestic yaks (Bos grunniens) on the Qinghai-Tibet Plateau, China. *Archives of virology* **2018**, *163*, 659-670, doi:10.1007/s00705-017-3658-9.
30. Yu., C.; Wang., X.; Zhou., Y.; Wang., Y.; Zhang., X.; Zheng., Y. Genotyping bovine leukemia virus in dairy cattle of Heilongjiang, northeastern China. *BMC Vet Res* **2019**, *15*, doi:https://doi.org/10.1186/s12917-019-1863-3.

31. Pluta, A.; Rola-Luszczak, M.; Kubis, P.; Balov, S.; Moskalik, R.; Choudhury, B.; Kuzmak, J. Molecular characterization of bovine leukemia virus from Moldovan dairy cattle. *Archives of virology* **2017**, *162*, 1563-1576, doi:10.1007/s00705-017-3241-4.
32. Molteni, E.; Agresti, A.; Meneveri, R.; Marozzi, A.; Malcovati, M.; Bonizzi, L.; Poli, G.; Ginelli, E. Molecular characterization of a variant of proviral bovine leukaemia virus (BLV). *Zentralblatt fur Veterinarmedizin. Reihe B. Journal of veterinary medicine. Series B* **1996**, *43*, 201-211.
33. Balic, D.; Lojkic, I.; Periskic, M.; Bedekovic, T.; Jungic, A.; Lemo, N.; Roic, B.; Cac, Z.; Barbic, L.; Madic, J. Identification of a new genotype of bovine leukemia virus. *Archives of virology* **2012**, *157*, 1281-1290, doi:10.1007/s00705-012-1300-4.
34. Lee, E.; Kim, E.J.; Ratthanophart, J.; Vitoonpong, R.; Kim, B.H.; Cho, I.S.; Song, J.Y.; Lee, K.K.; Shin, Y.K. Molecular epidemiological and serological studies of bovine leukemia virus (BLV) infection in Thailand cattle. *Infect Genet Evol* **2016**, *41*, 245-254, doi:10.1016/j.meegid.2016.04.010.
35. Polat, M.; Moe, H.H.; Shimogiri, T.; Moe, K.K.; Takeshima, S.N.; Aida, Y. The molecular epidemiological study of bovine leukemia virus infection in Myanmar cattle. *Archives of virology* **2017**, *162*, 425-437, doi:10.1007/s00705-016-3118-y.
